# Testing for differences in polygenic scores in the presence of confounding

**DOI:** 10.1101/2023.03.12.532301

**Published:** 2023-03-23

**Authors:** Jennifer Blanc, Jeremy J. Berg

**Affiliations:** 1 Department of Human Genetics, University of Chicago, Chicago, IL, USA

## Abstract

Polygenic scores have become an important tool in human genetics, enabling the prediction of individual phenotypes from their genotypes. Understanding how the pattern of differences in polygenic score predictions across individuals intersects with variation in ancestry can provide insights into the evolutionary forces acting on the trait in question, and is important for understanding health disparities. However, because most polygenic scores are computed using effect estimates from population samples, they are susceptible to confounding by both genetic and environmental effects that are correlated with ancestry. The extent to which this confounding drives patterns in the distribution of polygenic scores depends on patterns of population structure in both the original estimation panel and in the prediction/test panel. Here, we use theory from population and statistical genetics, together with simulations, to study the procedure of testing for an association between polygenic scores and axes of ancestry variation in the presence of confounding. We use a simple model of genetic relatedness to describe how confounding in the estimation panel biases the distribution of polygenic scores in a way that depends on the degree of overlap in population structure between panels. We then show how this confounding can bias tests for associations between polygenic scores and important axes of ancestry variation in the test panel. We then use the understanding gained from this analysis to develop a simple method that leverages the patterns of genetic similarity between the two panels to guard against these biases, and show that this method can provide better protection against confounding than the standard PCA-based approach.

## 1 Introduction

The calculation of polygenic scores [1] has become a routine procedure in many areas of human genetics. The promise of polygenic scores is that they provide a means for phenotypic prediction from genotype data alone. By measuring the association between a genetic variant and phenotype in a genome wide association study (GWAS), we obtain an estimate of its effect on the phenotype, averaged over the environments experienced by the individuals in that panel. These effect estimates can be combined into polygenic scores in a distinct prediction panel by taking a sum of the genotypes of individuals in that panel, weighted by the estimated effects. Under the relatively strict assumption that variation in the phenotype is not correlated with variation in ancestry the GWAS panel and that the prediction panel individuals experience a similar distribution of environments to the GWAS panel individuals, these scores can be viewed as an estimate of each individual’s expected phenotype, given their genotypes at the included sites. If this assumption is met, polygenic scores would seem to provide a means of partially separating genetic and environmental effects on a given phenotype.

However, this promise of polygenic scores is also one of their main pitfalls. The effects of individual variants are typically estimated from population samples in which the environments individuals experience vary as a function of their social, cultural, economic and political contexts. Differences in these factors are often correlated with differences in ancestry within population samples, and these ancestry-environment correlations induce systematic biases in the estimated effects of individual variants. Similar biases can also arise if genetic effects on the phenotype vary as a function of ancestry within the GWAS panel. Ancestry stratification is a long recognized problem in the GWAS study design [2], and many steps have been taken to guard against its effects. These include bias avoidance approaches, like the sampling of GWAS panels that are relatively homogeneous with respect to ancestry, to statistical bias correction approaches, like the inclusion of genetic principal components as covariates [3], linear mixed models [4, 5], and LD score regression [6]. While these approaches have largely been successful in minimizing the number of false positive single variant associations [7], effect size estimates can still exhibit slight stratification biases without significantly altering the false discovery rates for individual associations, and these biases can be compounded when aggregating across loci, ultimately leading to confounded predictions in which the ancestry associated effects are mistaken for direct genetic effects.

Separation of direct genetic effects from correlations between ancestry and environment or genetic background is important to all applications of polygenic scores. Empirically, polygenic scores exhibit geographic clustering even in relatively homogeneous samples and after strict control for population stratification [8, 9, 10, 11]. It is natural to ask if these observed differences reflect a real difference in the average genetic effect on the trait. From a population biology perspective, these patterns may be signals of natural selection or phenotype biased migration. Medically, it is interesting to know if polygenic score differences or gradients represent real underlying gradients in the average genetic effect [12], whether those gradients are caused by neutral evolutionary mechanisms or not. However, observed patterns of polygenic scores may also be driven by residual bias in effect size estimates, and stratification biases remain a persistent issue.

This issue has been particularly apparent in the detection of directional selection acting on complex traits. Polygenic scores are an ideal tool for this task, as studying the distribution of scores among individuals who differ in ancestry allows us to aggregate the small changes in allele frequency induced by selection on a polygenic trait into a detectable signal [13, 14, 15, 16]. Several research groups have developed and applied methods to detect these signals [17, 18, 19, 20, 21, 22, 23, 24]. However, these efforts have been met with challenges, as several papers reported signals of recent directional selection on height in Europe using effects obtained from GWAS meta-analyses [25, 26, 17, 18, 27, 28, 29, 20, 30, 31, 19], only for these signals to weaken substantially or disappear entirely when re-evaluated using effects estimated in the larger and more genetically homogeneous UK Biobank [32, 33, 22]. Further analysis suggested that much of the original signal could be attributed to spurious correlations between effect size estimates and patterns of frequency variation, presumably induced by uncorrected ancestry stratification in the original GWAS [32, 33]. Though more recent work using approaches that should be robust to this confounding have provided support for some of the original signals (see below) [34, 35], ancestry stratification remains a concern.

Recently, in the context of selection tests, Chen et al [34] proposed a strategy to mitigate the impact of stratification by carefully choosing the GWAS panel so that even if residual stratification biases in effect size estimates exist, they will be unlikely to confound the test (see also [36, 37] for examples of this approach). They reasoned that because polygenic selection tests ask whether polygenic scores are associated with a particular axis of population structure in a given test panel, and because the bias induced by stratification in effect sizes depends on patterns of population structure in the GWAS panel [27], then one should be able to guard against bias in polygenic selection tests by choosing GWAS and test panels where the patterns of population structure within the two panels are not expected to overlap.

However, this approach comes at a cost of reduced power: polygenic scores are generally less accurate when the effect sizes used to compute them are ported to genetically divergent samples [38, 39, 40, 41, 42]. Less accurate polygenic scores are then less able to detect a given polygenic selection event, all else equal. These decays in polygenic score accuracy also pose a significant challenge to their use in medicine, as scores that are predictive for some and not for others may exacerbate health inequities [43]. Thus, realizing the potential of polygenic scores in both basic science and medical applications will require the use of large and genetically diverse GWAS panels. Successfully deploying polygenic scores developed from these diverse panels will require that we have a precise understanding of how bias is produced in polygenic score predictions, and the development of precise methods to protect against this bias.

In this paper, we develop a general model for bias in polygenic score predictions and propose a simple method to correct for bias in tests for an association between polygenic scores and axes of ancestry variation. We model a joint sample of a GWAS and test panel in which we estimate effects in the former, then compute polygenic scores and test for association with an arbitrary axis of ancestry variation measured in the latter. We first characterize the bias in the distribution of the test panel polygenic scores as a function of the cross panel genetic similarity matrix, relying on simple and standard models for the distribution of phenotypes and genotypes across the two panels. We then show how the bias in the association between polygenic scores and a specific axis of ancestry variation in the test panel depends on a linear combination of the entries of the cross-panel genetic similarity matrix, where the test axis, measured in the test panel, specifies the combination. We then show that including this particular linear combination of genetic similarities as a covariate in the GWAS is sufficient to protect against bias in the association between polygenic scores and the chosen axis of ancestry variation in the test panel. We provide theoretical arguments linking this approach to the standard PCA approach of including top genetic principal components of the GWAS panel, and we show using simulations that our approach works when the PCA approach works, but also in cases where it fails. We then discuss several ways that this approach can provide clarity in polygenic score analyses.

## 2 Model

### 2.1 Genotypes and phenotypes

We assume that individuals in the GWAS panel are phenotyped and that the trait includes a contribution from a large number of causal variants, which make additive genetic contributions, as well as an independent environmental effect. The vector of mean-centered phenotypes for the *M* individuals in the GWAS panel can then be written

(1) $$y=\overset{P}{\underset{\ell }{\sum }}\beta_{\ell }G_{\ell }+e\;=u+e$$

where $u=\sum_{\ell }^{P}\beta_{\ell }G_{\ell }$ is the combined genetic effect of all P causal variants, and *e* represents the combination of all environmental effects.

In our model, the environmental effect on each individual is an independent Normally distributed random variable with variance 1 − *h*^2^. However, the expected environmental effect for each individual can differ in some arbitrary but unknown way across individuals, reflecting underlying differences in their socio-environmental context. We write the distribution of environmental effects as *e* ~ *MVN*(*c*, (1 − *h*^2^)**I**), where *c* is the vector of expected environmental effects.

Randomness in the genetic effect arises from segregation of genotypes within the panel, with a covariance structure given by relatedness matrix **F**_*GG*_. Again, however, the expected genetic effect can vary across individuals, reflecting the action of non-neutral processes such as natural selection, assortative mating, phenotype biased migration, or phenotype dependent ascertainment. We therefore write the distribution of genetic effects as *u* ~ *MVN*(*v*, *h*^2^**F**_*GG*_), where *v* is the vector of expected genetic effects across GWAS panel individuals given their ancestry and the set of non-neutral forces that have acted throughout the evolutionary history of the population. The sum *v* + *c* therefore gives the vector of individuals’ expected phenotypes, given their ancestry and socio-environmental contexts. We assume that these are not observed.

The genotypes at individual sites covary across panels due to shared ancestry. We assume that each variant was present at some frequency *λ*_*ℓ*_ in a set of shared ancestors common to both panels, and model the pattern of variation around the expected genotype (2*λ*_*ℓ*_) due to variation in relatedness. We write *X*_*ℓ*_ for the vector of centered genotypes measured in *N* test panel individuals, and *G*_*ℓ*_ for the vector of centered genotypes in the *M* GWAS panel individuals. The pattern of covariance at site *ℓ*, both within and across panels, is

(2) $$Var\left(\left[\begin{array}{c} X_{\ell }\\ G_{\ell } \end{array}\right]\right)=4\lambda_{\ell }\left(1-\lambda_{\ell }\right)F$$

where

(3) $$F=\left[\begin{array}{ll} F_{XX} & F_{XG}\\ F_{GX} & F_{GG} \end{array}\right]$$

is the joint relatedness matrix for the two panels. The matrices **F**_*GG*_ and **F**_*XX*_ describe within panel relatedness, while $F_{GX}=F_{XG}^{⊤}$ contains the cross panel coefficients. We assume that individual sites are unlinked, so that the only covariance among sites is that arising from shared ancestry among individuals, i.e. population structure.

### 2.2 Polygenic score association tests

We want to test the hypothesis that the genetic component of the phenotype contributed by a subset of *S* sites included in a polygenic score is associated with some test vector of interest in the test panel. The test vector, *T*, might represent an eco-geographic variable of interest (e.g latitude [18] or an encoding of whether one lives in a particular geographic region or not [9, 44]), the fraction of an individual’s genome assigned to a particular “ancestry group”[17, 20], or one of the top genetic principal components of the test panel genotype matrix [21], and is measured in the test panel. The genetic component contributed by the *S* sites included in the polygenic score is

(4) $$Z=\overset{S}{\underset{\ell }{\sum }}\beta_{\ell }X_{\ell }.$$

and we consider the linear model

(5) Z=qT+ε

with *ε* is i.i.d. Normal across individuals. A more powerful test is available by modeling the correlation structure among individuals (which is given by **F**_*XX*_), but the simpler i.i.d. model is sufficient for our purposes. We assume that the test vector, *T*, is scaled to have a variance of one, so the slope is given by

(6) q=cov(Z,T),

and we want to test the null hypothesis that *q* = 0.

To test the hypothesis, we estimate marginal effects, ${\hat{\beta }}_{\ell }$ (see below), and compute a polygenic score

(7) $$\hat{Z}=\overset{S}{\underset{\ell }{\sum }}{\hat{\beta }}_{\ell }X_{\ell }\text{. }$$

As a test statistic we consider the empirical covariance between the test vector and the polygenic scores, i.e.

(8) $$\hat{q}=\frac{1}{N-1}{\hat{Z}}^{⊤}T.$$

Under the null model, $E[\hat{q}]=0$, reflecting the fact that genetic drift is directionless, and has variance $V_{A}^{PGS}\left(\frac{1}{N}+f_{TX}\right)$, where $V_{A}^{PGS}=4\sum_{\ell =1}^{S}{\hat{\beta }}_{\ell }^{2}\lambda_{\ell }\left(1-\lambda_{\ell }\right)$ is the additive genetic variance of the polygenic score, and *f*_*TX*_ = *T*^⊤^ (*F*_*XX*_ − **I**)*T* captures additional variance arising from relatedness among test panel individuals. We compare this null hypothesis to an alternative in which $\hat{q}$ has an increased variance, reflecting the possibility that the polygenic score may have either a positive or negative association with the test vector. We refer to this as a “polygenic score association test”. In practice, our focus will be on $E[\hat{q}]$, as deviations from zero in the expectation of $\hat{q}$ under the null will lead to an inflated false positive rate.

We can also reframe our test as a statement about the association between the effect estimates and a set of genotype contrasts, $r_{\ell }=X_{\ell }^{⊤}T$, which measure the association between the test vector and the genotypes at each site. Then, writing *r* and $\hat{\beta }$ for the vectors of genotype contrasts and effect estimates, we can equivalently write our test statistic as

(9) $$\hat{q}=\frac{1}{N-1}{\hat{\beta }}^{⊤}r,$$

which will help make theoretical connections between different interpretations of our results and our method of correcting for stratification bias.

## 3 Results

Now, given these simple modeling assumptions, we describe how the relationship between the GWAS and test panels impact the distribution of polygenic scores and the association between the polygenic scores and the test vector. We first consider the case where no attempt is made to correct for population structure, before introducing our correction procedure and then discussing the role of the standard PCA approach in the context of our method.

### 3.1 Estimating marginal effects

Conditional on the genetic and environmental effects, and genotypes at the focal site, the marginal effect size estimate is given by

(10) $${\hat{\beta }}_{\ell }|G_{\ell },u,e=\frac{y^{⊤}G_{\ell }}{G_{\ell }^{⊤}G_{\ell }}\;=\frac{u^{⊤}G_{\ell }}{G_{\ell }^{⊤}G_{\ell }}+\frac{e^{⊤}G_{\ell }}{G_{\ell }^{⊤}G_{\ell }}\;=\sigma_{\ell }+\epsilon_{\ell }.$$

where *σ*_*ℓ*_ reflects the association between the total genetic effect on the trait and the genotype at the focal site (including the effect of the focal allele if it is causal) and *ϵ*_*ℓ*_ reflects the association with the environmental effect.

Additionally, we can decompose the genetic effect into the causal contribution from the focal site and the contribution from the background, i.e. *u* = *β*_*ℓ*_*G*_*ℓ*_+*u*_−*ℓ*_, so that *σ*_*ℓ*_ can be further decomposed as *σ*_*ℓ*_ = *β*_*ℓ*_ + *σ*_−*ℓ*_ where *σ*_−*ℓ*_ is the association between the focal site and the background contribution from all other sites. Because drift is directionless, an individual variant is as likely to be positively associated with either the genetic background or the environmental effect as it is to be negatively associated. As a result, the expected residual contribution to the marginal effect is zero for both the genetic and environmental contributions, i.e. $E\left[\sigma_{-\ell }|u_{-\ell }\right]=0$ and $E\left[\epsilon_{\ell }|e\right]=0$, so that the expected marginal effect is equal to the allele’s causal effect: $E\left[{\hat{\beta }}_{\ell }|u_{-\ell },e\right]=\beta_{\ell }$.

### 3.2 Stratification bias induces ancestry associated bias in polygenic scores

The deviation of an allele’s estimated causal effect size from its expectation depends on how the genotypes in the GWAS panel (*G*_*ℓ*_) deviate from their neutral expectation. Because these deviations are correlated with the deviations of the test panel genotypes (*X*_*ℓ*_) due to shared ancestry, the estimated effect sizes can become correlated with the pattern of genotypic variation in the test panel for reasons that have nothing to do with the actual genetic effect of the variant. This leads to a bias in the expectation of the polygenic scores, and in turn a bias in polygenic score association tests. The expectation of the vector of polygenic scores in the test panel (*Z*) is

(11) $$E{\left[Z\right]}^{⊤}\approx \frac{S}{\left(M-1\right)}\left(v^{⊤}+c^{⊤}\right){\tilde{F}}_{GX}$$

(supplement section 6.1) where

(12) $${\tilde{F}}_{GX}=E\left[\frac{G_{\ell }X_{\ell }^{⊤}}{G_{\ell }^{⊤}G_{\ell }/(M-1)}\right],$$

is the standardized cross panel genetic similarity matrix, where both the GWAS and test panel genotypes have been standardized by the variance in the GWAS panel, rather than by the variance in the combined sample.

### 3.3 Bias in polygenic scores leads to biased polygenic score associations

The bias in the polygenic score association $\left(\hat{q}\right)$ follows straightforwardly from the bias in the polygenic scores. The expectation of $\hat{q}$ is

(13) $$E[\hat{q}]=\frac{1}{N-1}E{\left[Z\right]}^{⊤}T\;\approx \frac{S}{\left(N-1)(M-1\right)}\left(v^{⊤}+c^{⊤}\right){\tilde{F}}_{GX}T.$$

We therefore expect the polygenic score association test to be biased when the test vector (*T*) aligns with the vector of expected phenotypes (*v* + *c*) in a space defined by the cross panel genetic similarity matrix $\left({\tilde{F}}_{XG}\right)$.

This result therefore provides explicit theoretical justification for the procedure of choosing GWAS and test panels that do not overlap in population structure as a means to avoiding bias in polygenic score analyses [1, 34, 45], as $E[\hat{q}]=0$ if ${\tilde{F}}_{XG}=0$. Any polygenic score association test performed using a pair of GWAS and test panels that meet this non-overlapping population structure criterion will therefore be unbiased, no matter what specific test is performed, or how the expected phenotypes are distributed. Stratification in the GWAS may still bias individual effects, but these residual biases are indistinguishable from noise from the perspective of the polygenic score association test. This can lead to a reduction in the accuracy of the polygenic scores, but does not incur bias.

Alternatively, recalling the site-by-site interpretation of the polygenic score association (Eq (9)), we can also express the bias as

(14) $$E[\hat{q}]=\frac{S}{\left(N-1)(M-1\right)}E\left[{\hat{\beta }}^{⊤}r\right]\;\approx \frac{S}{\left(N-1)(M-1\right)}\left(v^{⊤}+c^{⊤}\right){\tilde{F}}_{Gr}$$

where

(15) $${\tilde{F}}_{Gr}=E\left[\frac{G_{\ell }r_{\ell }^{⊤}}{G_{\ell }^{⊤}G_{\ell }/(M-1)}\right]\;={\tilde{F}}_{GX}T,$$

is a vector whose length is equal to the number of individuals in the GWAS panel (*M*). For each individual *m* in the GWAS panel, ${\tilde{F}}_{Gr,m}$ contains a linear combination of the their genetic similarities to the test panel individuals, where the combination is specified by the test vector. Alternatively, but equivalently, we can interpret ${\tilde{F}}_{Gr,m}$ as the expected correlation across sites between the standardized genotypes of GWAS panel individual *m* and the genotype contrasts of the association test (*r*), standardized by the square root of the variance in the GWAS panel. Expressing the bias in this way leads to two key insights. First, the test statistic is biased when the vector of expected phenotypes (*v* + *c*) aligns with the vector of correlations between GWAS panel genotypes and test panel genotype contrasts $\left({\tilde{F}}_{Gr}\right)$. Second, for a specific hypothesis corresponding to a specific set of genotype contrasts (*r*), we need only to ensure that the linear combinations of genetic similarities all sum to zero (i.e. that ${\tilde{F}}_{Gr}$ is a vector of zeros), which is a narrower condition than the complete non-overlapping population structure criterion (i.e. ${\tilde{F}}_{XG}=0$). While it would be difficult to meet this criterion via sample construction without also meeting the broader non-overlapping population structure criterion, it can be achieved statistically, which is the topic we turn to next.

### 3.4 Controlling for stratification bias in polygenic association tests

#### 3.4.1 A simple fixed-effects procedure to remove stratification bias

Given the above results, how can we ensure that patterns we observe in the distribution of polygenic scores are not the result of stratification bias? As discussed above, a conservative solution is to prevent bias by choosing a GWAS panel that does not have any overlap in population structure with the test panel, but this is not ideal due to the well documented portability issues that plague polygenic scores [46, 47, 42]. Another obvious solution is to include the vectors of expected genetic and environmental effects, *v* and *c* respectively, as covariates in the GWAS. Doing so would remove all ancestry associated bias from the estimated effects, and thus ensure that any polygenic score association test carried out using these effects would be unbiased. However, *v* and *c* are typically not measureable, so this is generally not an option.

Our analysis suggests a different approach. Namely, if ${\tilde{F}}_{Gr}$ is known, then including it as a co-variate in the GWAS will remove bias in effect size estimates that is correlated with the genotype contrasts relevant to the test (*r*). In this case, the OLS estimated marginal effect at site *ℓ*, conditional on *G*_*ℓ*_, *u* and *e*, is

(16) $${\hat{\beta }}_{\ell }^{\prime }|G_{\ell },u,e=\frac{u^{⊤}PG_{\ell }}{G_{\ell }^{⊤}PG_{\ell }}+\frac{e^{⊤}PG_{\ell }}{G_{\ell }^{⊤}PG_{\ell }}\;=\sigma_{\ell }^{\prime }+\epsilon_{\ell }^{\prime }.$$

where $P=\left(I-\frac{1}{{\tilde{F}}_{Gr}}{\tilde{F}}_{Gr}{\tilde{F}}_{Gr}^{⊤}\right)$ removes variation along the axis specified by ${\tilde{F}}_{Gr}$ from the estimated effects, and renders them uncorrelated with the genotype contrasts, *r*, under the null. If there is confounding along other shared axes of ancestry variation, the polygenic scores may still be biased along other axes, as

(17) $$E{\left[Z\right]}^{⊤}\approx \frac{S}{\left(M-1\right)}\left(v^{⊤}+c^{⊤}\right){\tilde{F}}_{GX}^{⊥{\tilde{F}}_{Gr}}$$

where

(18) $${\tilde{F}}_{GX}^{⊥{\tilde{F}}_{Gr}}\approx P{\tilde{F}}_{GX}$$

captures cross panel relatedness along all axes of variation other than that specified by ${\tilde{F}}_{Gr}$. However, regressing out variation aligned with ${\tilde{F}}_{Gr}$ ensures that ${\tilde{F}}_{GX}^{⊥{\tilde{F}}_{Gr}}T=0$, and it follows that

(19) $$E[\hat{q}]\approx \frac{S}{\left(N-1)(M-1\right)}\left(v^{⊤}+c^{⊤}\right){\tilde{F}}_{GX}^{⊥{\tilde{F}}_{Gr}T}\;\approx 0.$$

#### 3.4.2 Implementing the bias correction

In practice, ${\tilde{F}}_{Gr}$ is not known, but can be estimated from genome-wide data as a linear combination of the GWAS panel genotypes (*G*_*ℓ*_), weighted by the genotype contrasts of the test (*r*_*ℓ*_), each standardized by the variance in the GWAS panel

(20) $${\hat{F}}_{Gr}=\frac{1}{L}\overset{L}{\underset{\ell =1}{\sum }}\frac{G_{\ell }r_{\ell }}{G_{\ell }^{⊤}G_{\ell }/(M-1)}.$$

We then estimate marginal effects in the GWAS under the model

(21) $$y=G_{\ell }\beta_{\ell }+\gamma {\hat{F}}_{Gr}+\varepsilon ,$$

and then ascertain SNPs for inclusion in the polygenic scores via standard methods. In our simulations (see below and Methods), we also re-estimate their effects in a joint model as

(22) $$y=G_{S}\beta_{S}+\gamma {\hat{F}}_{Gr}+\varepsilon$$

where the *S* subscript indicates that only the ascertained sites are included. We then use this set of ascertained sites with jointly re-estimated effect sizes to perform the association tests in our simulated examples below.

#### 3.4.3 Predicting GWAS panel genotypes

In the previous sections, we characterized our approach as an effort to estimate a specific linear combination of the entries of the cross panel genetic similarity matrix between GWAS and test panels. In this section, we give a different interpretation of ${\hat{F}}_{Gr}$ in terms of a linear prediction of the standardized GWAS panel genotypes (i.e. ${\tilde{G}}_{\ell }=\frac{G_{\ell }}{\sqrt{G_{\ell }^{⊤}G_{\ell }/(M-1)}}$) given the set of genotype contrasts, also standardized by the GWAS panel genotype variance (i.e. ${\tilde{r}}_{\ell }=\frac{r_{\ell }}{\sqrt{G_{\ell }^{⊤}G_{\ell }/(M-1)}}$). We consider the matrix-variate regression model

(23) $$\tilde{G}=\rho {\tilde{r}}^{⊤}+\varepsilon \text{. }$$

In this model, $\tilde{G}$ is an *M* × *L* matrix containing the standardized genotypes $\left({\tilde{G}}_{\ell }\right)$ as columns, *ρ* is a vector of length *M* containing one regression coefficient per individual in the GWAS panel, $\tilde{r}$ is the length L vector of standardized genotype contrasts, and *ε* is the *M* × *L* matrix of residuals. The OLS estimate of *ρ* is

(24) $$\hat{\rho }=\frac{1}{{\tilde{r}}^{⊤}\tilde{r}}\sum_{\ell }{\tilde{G}}_{\ell }{\tilde{r}}_{\ell }.$$

Then, for each site *ℓ*, a linear prediction of ${\tilde{G}}_{\ell }$ given $\;\tilde{r_{\ell }}$ is equal to the product of $\hat{\rho }$ and $\;\tilde{r_{\ell }}$:

(25) $$E\left[{\tilde{G}}_{\ell }|{\tilde{r}}_{\ell }\right]=\hat{\rho }{\tilde{r}}_{\ell }$$

The expression for our estimate of ${\hat{F}}_{Gr}$ in equation (20) differs from the expression for $\hat{\rho }$ by only a scalar (*L*^−1^ in equation (20) vs ${\left({\tilde{r}}^{⊤}\tilde{r}\right)}^{-1}$ here), so we can intuitively interpret our bias correction method as including $E\left[{\tilde{G}}_{\ell }|{\tilde{r}}_{\ell }\right]$ as a covariate when estimating the effect at site *ℓ*.

#### 3.4.4. Relationship to PCA

A standard approach to controlling for population stratification in polygenic scores is to include the top *K* principle components of the GWAS panel genotype matrix as covariates in the GWAS, for some suitably large value of *K*. How does our approach relate to the this standard approach? In general, ${\hat{F}}_{Gr}$ can be written as a linear combination of the PCs of the GWAS panel genotype matrix, where the weights of this linear combination are a function of the covariance between genotype contrasts *r* and the SNP loadings for each PC.

Specifically, we can write ${\hat{F}}_{Gr}$ as

(26) $${\hat{F}}_{Gr}=\underset{i}{\sum }\alpha_{i}U_{i}$$

where *U*_*i*_ is the *i*^*th*^ PC of the standardized GWAS panel genotype matrix, and the weights are given by

(27) $$\alpha_{i}=\lambda_{i}cov\left(V_{i},\tilde{r}\right)$$

where *λ*_*i*_ is the *i*^*th*^ singular value of the standardized GWAS panel genotype matrix, *V*_*i*_ is the vector of SNP loadings for the *i*^*th*^ PC, and $\tilde{r}$ contains the standardized genotype contrasts (i.e. ${\tilde{r}}_{\ell }=\frac{r_{\ell }}{\sqrt{G_{\ell }^{⊤}G_{\ell }/\sqrt{\left(M-1\right)}}}$). Estimating the marginal associations with ${\hat{F}}_{Gr}$ as a covariate can therefore be understood as fitting a model in which *all* PCs of the GWAS panel genotype matrix are included as covariates, but the relative magnitude of the contributions from different PCs are fixed, and we estimate only a single slope that scales the contributions from all of the PCs jointly, i.e.

(28) $$y=G_{\ell }\beta_{\ell }+\gamma \left(\underset{i}{\sum }\alpha_{i}U_{i}^{\tilde{G}}\right)+e$$

where the *α*_*i*_ are fixed according to equation (27).

As a corollary, if we perform a polygenic score association test using GWAS effect size estimates in which the top *K* PCs of the GWAS panel genotypes are included as covariates, the necessary and sufficient condition for the included PCs to protect against bias from unmeasured confounders in a particular polygenic score association test is that ${\hat{F}}_{Gr}$ falls within the span of those top *K* PCs, i.e. if *α*_*i*_ ≈ 0 for *i* > *K*. An alternative interpretation of the PC correction approach is that it operates on a hypothesis that the major axes of confounding in a given GWAS panel (i.e. *v* + *c* in our notation) can be captured by the included PCs. A given association test will also be unbiased if this condition is met, but it is more difficult to assess whether this is the case.

#### 3.4.5 Downward bias with true signal

Our procedure differs from standard approaches for computing polygenic scores in that it uses the test panel genotype data twice: once when controlling for stratification in the GWAS panel, and a second time when testing for an association between the polygenic scores and the test vector. Should we not then worry that our procedure will regress out real signal when it exists? While this does happen, the effect will be small so long as the number of SNPs used to compute the correction is large relative to the number included in the polygenic score.

To see why, consider that we can rewrite our covariate in equation (21) in terms of a sum of the contribution from our focal site and all other sites as

(29) $$y=G_{\ell }\beta_{\ell }+\gamma \left(\frac{L-1}{L}{\hat{F}}_{Gr,-\ell }+\frac{1}{L}{\tilde{G}}_{\ell }{\tilde{r}}_{\ell }\right)+e$$

where ${\hat{F}}_{Gr,-\ell }$ is the estimate of ${\tilde{F}}_{Gr}$ that one would obtain using all sites other than the focal one. Thus, because our covariate includes a contribution from the focal site itself, controlling for it induces a slight bias in the estimated effect size, the sign of which depends on the sign of ${\tilde{r}}_{\ell }$. If ${\tilde{r}}_{\ell }$ is positive, ${\hat{\beta }}_{\ell }$ has a slight negative bias, whereas if ${\tilde{r}}_{\ell }$ is negative, the bias will be positive. A similar effect occurs in the context of correcting for PCs of gene expression data [48], and is the motivation for the “leave-one-chromosome-out” approach to fitting linear mixed models [49]. Assuming that the variance of the ${\tilde{r}}_{\ell }$ across sites for those included in the score is the same as those used to compute the correction, the product $r_{\ell }{\hat{\beta }}_{\ell }$ will be biased toward 0 by a factor of approximately $\left(1-\frac{1}{L}\right)$ for each site, owing to the fact that the focal site contributes approximately $\frac{1}{L}$ of our estimate ${\hat{F}}_{Gr}$. Our test statistic is a sum over contributions of $r_{\ell }{\hat{\beta }}_{\ell }$ from *S* independent sites, so including ${\hat{F}}_{Gr}$ as a covariate when estimating effect sizes induces a downward bias of approximately $\left(1-\frac{S}{L}\right)$, i.e.

(30) $$E[\hat{q}|q]\approx q\left(1-\frac{S}{L}\right).$$

where *q* is the value of our test statistic we would expect to obtain if the causal effects were known. Our procedure is therefore expected to remove a small amount of signal alongside the stratification bias, but this effect will be minimal as long as the number of sites used to compute ${\hat{F}}_{Gr}$ far exceeds the number of sites included in the polygenic score. The key principle is that when there is overlapping structure between the GWAS and test panels, our ability to separate real signal from stratification bias depends on an assumption that the signal is sparse, but the confounding is dense. Notably, because the standard PCA approach protects against stratification bias in the test by capturing the same axis of cross panel variation (see above), it will induce the same downward bias.

Real populations exhibit linkage disequilibrium, so the appropriate value of *L* corresponds to an “effective” number of independent sites, rather than the actual total number of sites used to compute the correction. For human population samples imputed to common reference panels, the effective number of SNPs is typically on the order of at least half a million [50], suggesting that even for a polygenic score that included, for example, 10,000 SNPs, the downward bias should be no more than 2%. Further concern about downward biases in applications could be amerliorated via the “leave one chromosome out” scheme [49], in which an estimate of ${\hat{F}}_{Gr}$ for each of the 22 autosomes would be computed using only genotypes from the other 21.

### 3.5 Applications: Theory and Simulations

In this section, using both theory and simulations, we consider a number of concrete examples with varying degree of alignment between the axis of stratification and axis of population structure relevant to the polygenic association test, demonstrating how these biases play out in practice, and how our proposed correction reduces false positives to nominal levels.

#### 3.5.1 Toy Model

**Figure 1:**
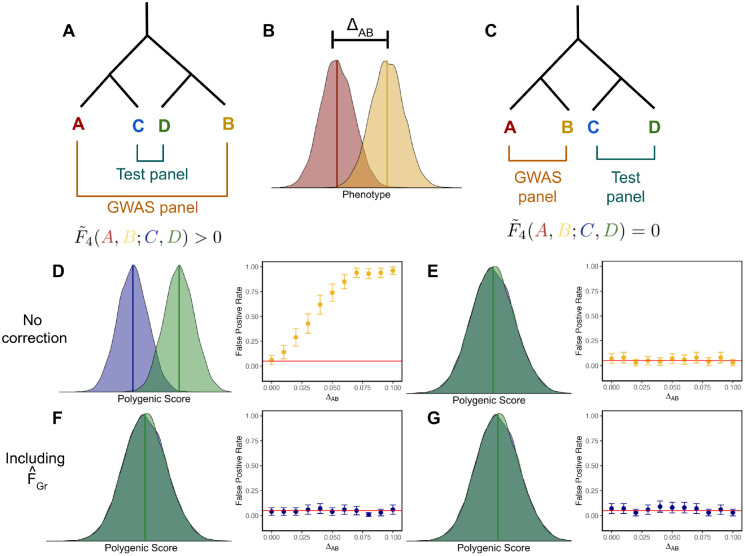
Schematic of two different panel configurations. The effect of stratification depends on the overlapping structure between the GWAS and test panels. (A) and (C) Two different topologies used to create the GWAS and test panels. (B) Stratification was modeled in the GWAS panel by drawing an individual’s phenotype *y* ~ *N*(0, 1) and adding Δ_*AB*_ if they originated from population B. (D) When there is overlapping structure between GWAS and test panels, there is an expected mean difference between polygenic scores in populations C and D. Additionally, the false positive rate for $\hat{q}$ increases with the magnitude of stratification in the GWAS. (E) However, when there is no overlapping structure between panels, there is expected difference in mean polygenic scores between C and D and the false positive rate remains at 5% regardless of the magnitude of stratification. (F) and (G) Including ${\hat{F}}_{Gr}$ as a covariate in the GWAS controls for stratification, keeping the false positive at nominal levels regardless of Δ_*AB*_ or the overlapping structure between GWAS and test panels. False positive rates were calculated as the number of simulations with *p* < 0.05 out of 100 simulations for each Δ_*AB*_.

We first consider a toy model with four populations (labelled A, B, C and D), which are related to one another by an evenly balanced population phylogeny (Figure 1) with no structure within each of the populations. The GWAS panel is composed of an equal mixture of individuals from populations A and B, and we test for a difference in mean polygenic score between populations C and D under two different topologies, one where A and C are sister to one another (Figure 1A), and another where A and B are sister (Figure 1C). For simplicity, we simulate a purely environmental phenotype (i.e. *h*^2^ = 0) with a difference in mean between populations A and B equal to Δ_*AB*_, so that $e_{i}=\frac{\Delta_{AB}}{2}+\varepsilon_{i}\;$ if individual *i* belongs to population A, and $e_{i}=-\frac{\Delta_{AB}}{2}+\varepsilon_{i}$ if they belong to population B, where *ε*_*i*_ ~ *N*(0, 1) (Figure 1B). Following from equation (10), the marginal effect size estimate for site *ℓ* is then

(31) $${\hat{\beta }}_{\ell }|G_{\ell },e=\frac{G_{\ell }^{⊤}e}{G_{\ell }^{⊤}G_{\ell }}\;=\frac{1}{2}\frac{\Delta_{AB}\left(p_{A,\ell }-p_{B,\ell }\right)}{G_{\ell }^{⊤}G_{\ell }/(M-1)}+\frac{G_{\ell }^{⊤}\varepsilon }{G_{\ell }^{⊤}G_{\ell }}$$

where *p*_*A,ℓ*_ and *p*_*B,ℓ*_ are the population allele frequencies at site *ℓ* (see also equation 2.3 in the supplement of [51]).

Then, using these these effect sizes to test for a difference between populations C and D. The expected value of $\hat{q}$ is

(32) $$E[\hat{q}]=\Delta_{AB}\overset{S}{\underset{\ell =1}{\sum }}E\left[\frac{\left(p_{A,\ell }-p_{B,\ell }\right)\left(p_{C,\ell }-p_{D,\ell }\right)}{G_{\ell }^{⊤}G_{\ell }/(M-1)}\right]\;=\Delta_{AB}S{\tilde{F}}_{4}(A,B;C,D)$$

where ${\tilde{F}}_{4}(A,B;C,D)$ is a version of Patterson’s *F*_4_ statistic, [52, 53] standardized by the genotypic variance in the GWAS panel, and measures the amount of genetic drift common to population A and B that is also shared by populations C and D. Writing the expectation of $\hat{q}$ in terms of this modified *F*_4_ statistic helps illustrate the role of cross panel population structure in driving stratification bias in polygenic scores for a simple example. The effect estimate at site *ℓ* is a linear function of *p*_*A,ℓ*_ − *p*_*B,ℓ*_, so the resulting selection test will be biased if *p*_*A,ℓ*_ − *p*_*B,ℓ*_ is correlated with *p*_*C,ℓ*_ −*p*_*D,ℓ*_. This is true for the demographic model in Figure 1A, where shared drift on the internal branch generates such a correlation, yielding a positive value for ${\tilde{F}}_{4}(A,B;C,D)$ and therefore a biased test statistic, but not for the model in Figure 1B, where there is no shared internal branch, ${\tilde{F}}_{4}(A,B;C,D)=0$, and the test statistic is unbiased.

To test this prediction, we simulated 100 replicates of four populations related by this topology. In each simulation, populations on the same side of the tree are separated by 100 generations of evolution, while populations on opposite sides are separated by 200 generations. The diploid population size is 10,000 for each population and in the ancestral populations throughout the simulation, so the *F*_*ST*_ between pairs of populations on the same side of the tree is approximately 0.005, and 0.01 for populations on opposite sites. In contrast to our theoretical model, in our simulations we include linkage over short scales by simulating 200 independent 1Mb linkage blocks per simulation replicate. We then simulated purely environmental phenotypes in the GWAS panel populations (A and B), with a difference in mean phenotype, as outlined above, conducted a GWAS to ascertain SNPs, and then used these ascertained SNPs to construct polygenic scores and test for a difference in mean between populations C and D. To assess whether the difference was significantly different from zero, we compare to an empirical null distribution constructed by permuting the signs of the of the effect estimates across sites (see Methods). The results are consistent with our theoretical expectations: the test is biased for the topology with ${\tilde{F}}_{4}(A,B;C,D)>0$ (Figure 1D), but unbiased when ${\tilde{F}}_{4}(A,B;C,D)=0$ (Figure 1E).

We then use all genome-wide SNPs with a frequency of greater than 5% in both the test and GWAS panel to estimate ${\hat{F}}_{Gr}$, as in equation (20). The resulting vector is, as expected, tightly correlated with population label for topology with ${\tilde{F}}_{4}(A,B;C,D)>0$, but reflects random noise for the case when ${\tilde{F}}_{4}(A,B;C,D)=0$ (Figure S2). When we re-run the polygenic score association test using effects estimated with ${\hat{F}}_{Gr}$ included as a covariate, the bias in $\hat{q}$ is removed (Figure S1), and false positive rates are restored to nominal levels for the confounded topology (Figure 1F). For the unconfounded topology, the test is already unbiased, so the correction has no effect (Figure 1G).

Next, we sought to confirm that including ${\hat{F}}_{Gr}$ does not regress out true signals of polygenic score divergence, consistent with our theoretical argument above. To do this, we modified our simulations by adding causal loci to make the trait heritable, with *h*^2^ = 0.3, and sampled the sign of the effect for these causal loci so as to generate a correlation between the effect and the frequency differences between populations C and D. We compare the true positive rate across simulations under three different schemes for obtaining effect sizes: 1) estimating effects with no bias correction, 2) including ${\hat{F}}_{Gr}$ as a covariate, or 3) including known population ID in the GWAS. Correcting for population ID is included as a gold standard for this simple model, but would be unrealistic for a real sample as real populations do not conform to idealized population models. For each scheme, we consider both the case where the identity of the causal variants are known but the effects are estimated vs ascertaining the most significant variant per LD block using p-values. We compare all of these schemes to association tests performed using the true causal variants with their true causal effect sizes.

**Figure 2:**
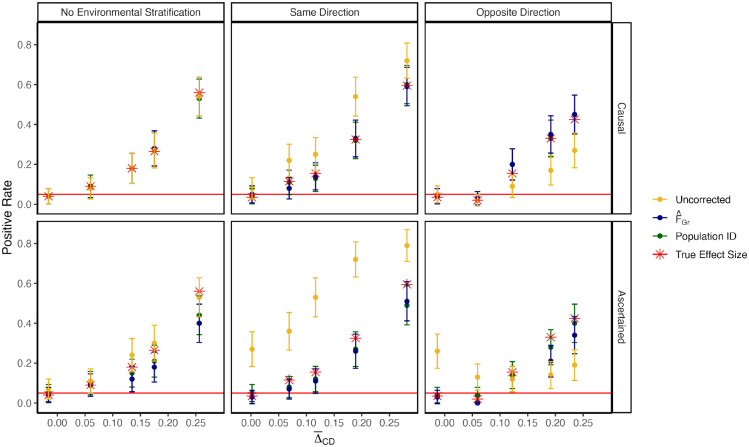
Including ${\hat{F}}_{Gr}$ as a covariate in the GWAS model does not regress out true signal. Here we generate a true difference in average polygenic scores by flipping the sign of a proportion of causal effect sizes to align with allele frequency contrasts, *p*_*D,ℓ*_ − *p*_*C,ℓ*_, in the test panel. ${\bar{\Delta }}_{CD}$ is the true difference in polygenic scores averaged across 100 replicates. The top row is the positive rate in our association test using estimated effect sizes at causal sites. Regardless of the direction of the stratification effect, our correction procedure perfectly captures the real signal (computed using the true effect sizes). The bottom row is the positive rate when polygenic scores were computed using sites ascertained on p-value. While there is some loss of power due to imperfect tagging, our approach maintains power to detect true signal.

When the identities of the causal sites are known and there is no environmental stratification, all three schemes for estimating effetcts perform similarly, and equally as well as when the causal effects themselves are known exactly (Figure 2, top left). As expected, when the environmental stratification is in the same direction as the true mean difference, the uncorrected effect sizes reject the null at a higher rate than when the causal effects are known, while our method and population ID perform as well as having the causal effects (Figure 2, top middle). The same is true when the environmental effect is in the opposite direction as the true signal, except the uncorrected effects have reduced power, as expected (Figure 2, top right). When the identities of the causal variants are unknown and associated sites must be ascertained, we observe largely similar patterns, with our method and the population ID method having slightly reduced power relative to when the identities of the causal variants are known (Figure 2, bottom row). Our simulation results support our theoretical argument that including ${\hat{F}}_{Gr}$ does not remove real signals when *S* ≪ *L*.

#### 3.5.2 Grid Simulations

To further explore our correction procedure in more complex scenarios, we conducted another set of coalescent simulations under a symmetric two-way migration model on a six-by-six lattice grid. We sampled an equal number of individuals per deme to comprise both the GWAS and test panels and then simulate several different distribution of purely environmental phenotypes across the GWAS panel individuals. We consider three different scenarios for the distribution of phenotypes: one where the mean varies with latitude, one where it increases only along the diagonal of the grid, and one in which only a single deme has an elevated mean, a scenario which is difficult to correct for with standard tools [54, 55]. For each scenario, we estimate effect sizes, ascertain associated sites, and test for an association between polygenic score and latitude, longitude, or membership in the single confounded deme, depending on the example.

**Figure 3:**
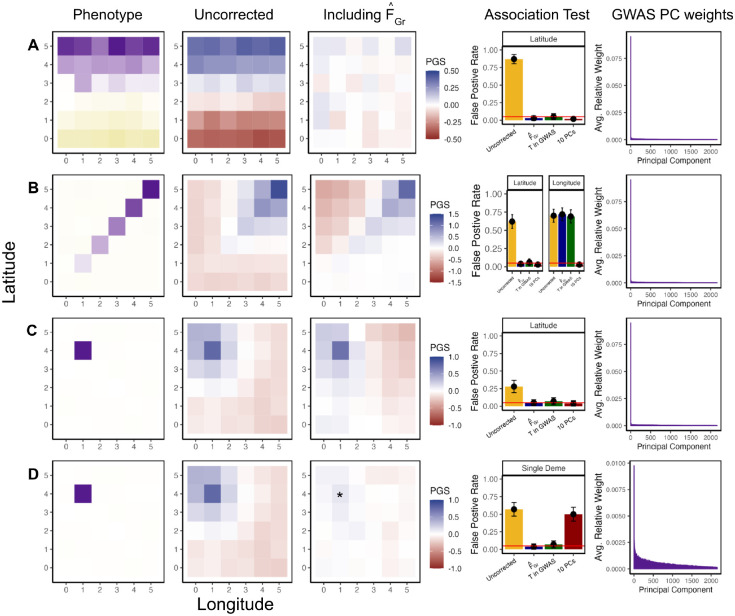
Including ${\hat{F}}_{Gr}$ as a covariate in the GWAS model controls for stratification bias specific to the association test of interest. GWAS and test panel individuals were simulated using stepping-stone model with continuous migration. In the GWAS panel the phenotype is non-heritable and stratified along either latitude (A), the diagonal (B), or in a single deme (C, D). When effect sizes are estimated in a GWAS with no correction for stratification, polygenic scores constructed in the test panel recapitulate the spatial distribution of the confounder (second column). Including ${\hat{F}}_{Gr}$ (latitude for A-C, belonging to * deme for D) in the GWAS model eliminates bias in polygenic scores along the test axis (third column) and the false positive rate for $\hat{q}$ cross 100 replicates remains at nominal levels (fourth column). We also compare our approach to including the top 10 PCs and including the test axis, as define in the GWAS panel (latitude for A-C, single deme for D), directly. Finally we plot the relative weight of all *M* − 1 GWAS panel PCs on ${\hat{F}}_{Gr}$, averaged across 100 replicates (fifth column). When the test axis is latitude (A-C), ${\hat{F}}_{Gr}$ loads mainly onto one of the first two PCs, while a single deme test (D) has weight on the full range of PCs.

For the first example, we simulated a latitudinal confounder such that *c*_*i*_ is a linear function of an individual’s position on the latitudinal axis (Figure 3A). When we do an uncorrected GWAS and use those effect sizes to build polygenic scores in the test panel, their spatial distribution reflects the distribution of the environmental confounder. Consequently, an association test using latitude as the test vector is biased. However, when we compute ${\hat{F}}_{Gr}$ and include it as a covariate in the GWAS model, effect sizes are unbiased with respect to the latitudinal genotype contrasts in the test panel and the resulting association test is unbiased.

In the second example, we simulate confounding along the diagonal, resulting in uncorrected polygenic scores that are systemically correlated with both latitude and longitude in the test panel and the association test is biased along both axes (Figure 3B). However, when we compute ${\hat{F}}_{Gr}$ using latitude as the test vector, the resulting effect sizes are only uncorrelated with latitudinal genotype contrasts and polygenic scores remain susceptible to bias along any other axis (e.g. longitude). This example highlights the targetted nature of this method, as using effect sizes from a GWAS including ${\hat{F}}_{Gr}$ does not remove all bias, but does makes the association test using those effect sizes for the pre-specified test vector unbiased.

In the first two examples, the environmental confounder reflected broad geographic structure and could therefore be easily captured by the first two principal components of the GWAS panel. Thus, while our approach was effective, the standard PCA method was as well. In our next example we simulated single deme confounder which induces a more complex spatial pattern in the uncorrected polygenic scores (Figure 3C). Taking latitude to be the test vector, ${\hat{F}}_{Gr}$ again protects the selection test from bias only along the latitudinal axis. In this case, while the confounder is not fully captured by the first 10 principle components (see Figure S5), including 10 PCs in the GWAS successfully controls for stratification because ${\hat{F}}_{Gr}$ falls within their span.

In our final example, we again simulated a single deme confounder, but this time took the test vector to be an indicator for whether the test panel individuals were sampled from that deme or not. In this case, the test vector cannot be captured by the top 10 PCs (or, indeed, a much larger numbers of PCs, see Figure S3), so this method fails, while our method succeeds at protecting the test from bias. Conceptually, ${\hat{F}}_{Gr}$ is more successful than top principal components in this case because it is able to capture more subtle structure in the GWAS panel. We also tested our approach for each of these for scenarios over a range of magnitudes for the confounding effect. In our simulations, ${\hat{F}}_{Gr}$ protects all association tests from confounding effects of up to two phenotypic standard deviations, whereas the top 10 principle components begin to fail in some scenarios for more modest strengths of the confounding effect (Figure S4).

## 4 Discussion

Understanding associations between polygenic scores and patterns of ancestry variation is important to many areas of human genetics. Because most well-powered GWAS are conducted on population samples in which the relationship between environment and ancestry is not well controlled, stratification bias remains a significant concern [32, 33, 42, 56]. Here, we characterize patterns of stratification bias in the distribution of polygenic scores as a function of the genetic similarity between GWAS and test panels, and show how test panel genotypes can be used to protect against stratification bias when testing hypotheses about the relationship between polygenic scores and ancestry. The advantage of this approach is that it can succeed in situations where PCA fails because the bias lies along an axis of variation in the GWAS panel which is not captured by the top PCs.

Another advantage of our approach has to do with the tradeoff between statistical power and robustness in polygenic selection analyses, as researchers motivated by the difficulties with stratification bias and height [32, 33] have tended to take conservative approaches meant to trade away statistical power in exchange for robustness to biases due to ancestry stratification. This includes the non-overlapping population structure study design [34], but also choices such as focusing only on genome wide significant hits, or throwing away all effect size information except for the sign. An advantage of the stronger guarantee against bias due to ancestry stratification provided by our method is thus that it may enable the use of less conservative methods for computing polygenic scores, which do not trade away so much statistical power.

The disadvantage of the approach is that it requires that the test panel genotype are available and the test axis specified before carrying out the GWAS. Thus, the benefits of our approach cannot immediately be extended to situations where the individual level data for both panels is not available. Tests for association between polygenic scores and axes of ancestry variation are closely related to bivariate LD score regression as applied to a combination of effect estimates for one trait and frequency/genotype contrasts from an independent dataset [57, 19, 32], an approach which does not require the individual level genotypes, but does require access to ancestry-matched LD scores. Previous work in the context of polygenic selection tests raised concerns about inflation of the slope in this approach due to background selection [32]. A more complete analysis of bivariate LD score regression applied in this context would be welcome.

We have focused on analyses of the relationship between polygenic scores and patterns of ancestry variation, but our results are relevant for polygenic prediction more generally. For example, our approach could be used to protect polygenic score predictions from bias associated with ancestry associated socio-cultural variables measured in a particular prediction panel, even if those variables are not measured the GWAS panel. A related idea is to control for axes of ancestry variation identified in a large external reference dataset that captures as broad of a swath of human genetic variation as possible [58].

Our results are also relevant for analyses that use GWAS summary statistics and coalescent methods to test for signals of directional polygenic selection on variants associated with a particular trait [19, 23, 59, 60]. One way of thinking about these coalescent approaches is to recognized that they use patterns of haplotype variation to estimate genotype contrasts between the sampled present day individuals and a set of unobserved ancestors, and then ask whether these estimated genotype contrasts correlate with effect size estimates for a trait of interest. Thus, if the relevant genotype contrasts are estimated using the coalescent methods before conducting the GWAS, then these estimated genotype contrasts could be used to immunize the coalescent analysis against stratification bias, similar to how we immunize specific polygenic score comparisons in this paper.

A related point is that for both the coalescent approaches as well as methods relying on direct comparison of polygenic scores, both the evolutionary hypothesis being tested and the degree of susceptibility to bias follow directly from the set of genotype contrasts used in the test. Some authors have suggested that certain coalescent methods of testing for polygenic selection are more robust to stratification bias than others [59, 60], but our results show that this cannot be true: two different methods that test the same evolutionary hypothesis using the same set of estimated effect sizes necessarily have the same susceptibility to stratification bias. Therefore, any observed differences in robustness to stratification bias among methods must come either from changing the evolutionary hypothesis being tested or from differences in ability to accurately estimate the relevant genotype contrasts (i.e. overall differences in statistical power).

We also note several limitations of our approach. First, our model assumes that all loci share the same underlying genetic relatedness matrix. For simple demographic histories like that of our toy model, this is unlikely to be an issue, but could be an issue for more complex demographic histories experienced by real human populations, as mutations of different ages experience the demographic history differently. One solution to this problem would be to bin variants by allele frequency or estimated age [61] and compute different corrections for different frequency/age bins similar to the suggestion by [55] to use principal components computed on rare variants to correct for stratification along axes of recent genetic divergence.

Another limitation is that in real human datasets with complex patterns of genetic variation and confounding, our approach alone will not be sufficient to mitigate all of the negative consequences of confounding. For example, while a GWAS conducted using only our covariate to control for stratification biases would result in effect size estimates that are uncorrelated with the target axis of population structure under the null, stratification along other axes would still function as additional noise in the effect size estimates. This would result in the ascertainment of tag SNPs that are less tightly coupled to their causal variants, and less accurate polygenic scores, reducing power to detect real signals. Therefore, control of population structure via inclusion of top PCs and/or linear mixed models, in addition to the inclusion of our projected test vector, is still desirable.

Finally, we note that the interpretation of results derived from applying our method will be limited by standard assumptions made in many polygenic score association tests and caution interpretation in light of these assumptions [62]. Notably, we rely on common tag SNPs, focus on the additive genetic component of a trait, and ignore the potential impact of interactions, either among genetic variants, or between genetic variants and the environment. Further, GWAS can also be impacted by other forms of genetic confounding beyond the simple associations between ancestry and genetic background that we consider here, including that arising from indirect effects, assortative mating, and stabilizing selection [63]. Finally, the interpretation of associations between polygeic scores and axes of ancestry variation remains challenging in general, as differences in polygenic scores do not necessarily translate into differences in phenotype [12], and associations between polygenic scores and patterns of ancestry variation may have counterintuitive evolutionary explanations [64, 41, 40]. Therefore, while our results provide a means to protect against stratification bias in polygenic score association tests, addressing a known problem in their implementation, continued care in the interpretation of these analyses is warranted.

## 5 Materials and Methods

### 5.1 Simulating genotypes

We used *msprime* [65] to simulate genotypes under 2 different models with 100 replicates per model. The first model, shown in Figure 1, has two population splits, 200 and 100 generations in past, for a total of 4 present day populations. We fix the population size for all present and past populations to 10,000 diploid individuals. Each replicate consists of 200 independent 1Mb chromosomes with per base mutation and recombination rates equal to 1 × 10^−8^. We then sample 20,000 individuals per population and create two configurations of GWAS and test panels based on the diagrams in Figure 1A and Figure 1C. We down sample the test panels to 2,000 individuals with an equal number from each of the two populations. Finally, we filter out variants with MAF < 0.05 in either panel from both panels. When the populations in the GWAS and test panel are non-sister (i.e Figure 1A) the average *F*_*ST*_ [66] was 0.01, whereas in the configuration in Figure 1C the average *F*_*ST*_ was 0.05.

The second model, modified from [67], is a 6 × 6 stepping stone model where structure extends infinitely far back with a symmetric migration rate of *m* = 0.01. As above, we fix the effective population size to 10,000 diploid individuals and simulate 200 chromosomes per individual. To reduce computational burden, we scale down to 10kb chromosomes with a per base mutation and recombination rates equal to 1 × 10^−7^. We then sample 20 individuals per deme to create a test panel (*N* = 720) and 60 individuals per deme to create the GWAS panel (*M* = 2160). Again we filter out SNPs with MAF < 0.05 in either panel.

#### 5.1.1 Simulating phenotypes

To study the effect of environmental stratification on association tests, we first simulated nongenetic phenotypes for an individual *i* in the GWAS panel as *y*_*i*_ ~ *N*(0, 1). In our discrete 4 population model (Figure 1) we then generate a phenotypic difference between populations by adding Δ_*AB*_ to *y*_*i*_ for individuals in population B. In our grid simulations we generate a phenotypic gradient by adding $\frac{j\Delta }{J}$, to individuals in *J* specific grid squares depending on the desired stratification pattern (see Figure 3 and Figure S4) where Δ varies from 0 to 2 standard deviations.

To study the effect of controlling for stratification in cases where there is a true signal of association between polygenic scores and the test vector (Figure 2), we used our 4 population demographic model and followed the protocol outlined in [67] to simulate a neutral trait with *h*^2^ = 0.3. We first randomly select 1 causal variant per chromosome and sample their effect sizes $\beta_{\ell }\sim N\left(0,\sigma_{i}^{2}{\left[p_{\ell }(1-p_{\ell })\right]}^{\alpha }\right)$. $\sigma_{i}^{2}$ is the frequency independent component of genetic variance, *p*_*ℓ*_ is allele frequency in the GWAS panel, and *α* is a scaling factor controlling the relationship between allele frequency and effect size. We set *α* = −0.4 and $\sigma_{g}^{2}=\sigma_{i}^{2}\sum_{\ell =1}^{200}{\left[2p_{\ell }\left(1-p_{\ell }\right)\right]}^{\alpha +1}=0.3$. To simulate a signal of true difference in polygenic score in the test panel, we calculate the frequency difference *p*_*D,ℓ*_ − *p*_*C,ℓ*_ at all 200 causal sites in the test panel and flip the sign of the effect sizes in the GWAS panel such that *p*_*D*_ −*p*_*C*_ > 0 and *β*_*ℓ*_ > 0 with probability *θ*. *θ* therefore controls the strength of the association with *θ* = 0.5 representing no expected association and *θ* = 1 representing the most extreme case where trait increasing alleles are always at a higher frequency in population D. We then draw the environmental component of the phenotype *e*_*i,k*_ ~ *N*(*μ*_*k*_, 1 − *h*^2^) where again *μ*_*k*_ = 0 for individuals in population A and *μ*_*k*_ = Δ_*AB*_ for individuals in population B.

#### 5.1.2 Computing ${\hat{F}}_{Gr}$

In order to increase the precision in our estimates of the genotype contrasts, and therefore of ${\hat{F}}_{Gr}$, we first standardize the test panel genotypes and test vector by an estimate of the test panel genetic similarity matrix. We therefore compute $r^{*}=X^{⊤}{\hat{F}}_{XX}^{-1}T$ where we first calculate ${\hat{F}}_{XX}$ using the plink2 [68] function --make-rel cov square and then invert ${\hat{F}}_{XX}$ using eigen() in R. Next, we multiply by *T* and use the plink2 function --glm to compute r*. Finally we compute ${\hat{F}}_{Gr}$ as stated in (20) using --sscore.

#### 5.1.3 GWAS and SNP ascertainment

For each set of phenotypes, we first carried out four separate marginal association GWASs using the regression equations below,

1. *y* = *β*_*ℓ*_*G*_*ℓ*_ + *ϵ*
2. $y=\beta_{\ell }G_{\ell }+\gamma {\hat{F}}_{Gr}+\epsilon$
3. *y* = *β*_*ℓ*_*G*_*ℓ*_ + *γ*ID + *ϵ*
4. *y* = *β*_*ℓ*_*G*_*ℓ*_ + *γ*_1_*PC*_1_ + … + *γ*_*k*_*PC*_*k*_ + *ϵ*

where *ID* contains the test axis of interest as measured directly in the GWAS panel (either population ID, latitude or the single deme of interest). This set-up allows us to compare our covariate ${\hat{F}}_{Gr}$ to a GWAS done with no correction and one done with perfect correction for the test of interest. Additionally, we compared our results to a model that includes some number of top genetic principal components in the GWAS panel. All GWASs were done using the plink2 [68] function --glm.

We then ascertain 200 SNPs to do our association test by picking the SNP with the lowest p-value per chromosome. Additionally, for heritable trait simulations, we know the identity of the underlying causal sites and can choose to use those sites (Figure 2). For each set of sites, either those chosen by p-value or causal sites, we use R to re-estimate effect sizes jointly using the lm() under the four models above where we instead include all 200 SNPs along with the covariates.

#### 5.1.4 Polygenic Score Association Test

For each replicate we first compute the test statistic $\hat{q}=\frac{{\hat{\beta }}^{⊤}r^{*}}{\sqrt{N{\hat{V}}_{A}^{PGS}}}$ by multiplying the set of ascertained effect size estimates by r* for corresponding site, and calculating $V_{A}^{PGS}=4\sum_{\ell =1}^{S}{\hat{\beta }}_{\ell }p_{\ell }\left(1-p_{\ell }\right)$ where *p*_*ℓ*_ is the allele frequency in the test panel.

To evaluate the significance of our association statistic, we compute p-values using an empirical null approach. Specifically, we obtain psuedosamples of $\hat{q}$ by randomizing the sign of the effect sizes and recomputing $\hat{q}$. We then obtain a p-value from this sign-flipping null as,

(33) $$P=2*\frac{\sum_{i}^{N}I\left(\left|{\hat{q}}_{i}\right|>\left|{\hat{q}}_{obs}\right|\right)}{K},$$

where *K* is the number of psuedosamples, here set to 1,000, and *I*() is an indicator function that equals 1 if the psuedosample $\left|{\hat{q}}_{i}\right|$ is greater than the observed $\left|{\hat{q}}_{obs}\right|$. Finally within a set of 100 evolutionary replicates for each model and confounder combination we compute the positive rate as the proportion of p-values <= 0.05.

## Supplementary Material

1
