# Testing for differences in polygenic scores in the presence of confounding

**DOI:** 10.1101/2023.03.12.532301

**Published:** 2023-08-22

**Authors:** Jennifer Blanc, Jeremy J. Berg

**Affiliations:** 1 Department of Human Genetics, University of Chicago, Chicago, IL, USA

## Abstract

Polygenic scores have become an important tool in human genetics, enabling the prediction of individuals’ phenotypes from their genotypes. Understanding how the pattern of differences in polygenic score predictions across individuals intersects with variation in ancestry can provide insights into the evolutionary forces acting on the trait in question, and is important for understanding health disparities. However, because most polygenic scores are computed using effect estimates from population samples, they are susceptible to confounding by both genetic and environmental effects that are correlated with ancestry. The extent to which this confounding drives patterns in the distribution of polygenic scores depends on patterns of population structure in both the original estimation panel and in the prediction/test panel. Here, we use theory from population and statistical genetics, together with simulations, to study the procedure of testing for an association between polygenic scores and axes of ancestry variation in the presence of confounding. We use a general model of genetic relatedness to describe how confounding in the estimation panel biases the distribution of polygenic scores in a way that depends on the degree of overlap in population structure between panels. We then show how this confounding can bias tests for associations between polygenic scores and important axes of ancestry variation in the test panel. Finally, we use the understanding gained from this analysis to develop a method that uses patterns of genetic similarity between the two panels to guard against these biases, and show that this method can provide better protection against confounding than the standard PCA-based approach.

## Author Summary

Complex traits are influenced by both genetics and the environment. Human geneticists increasingly use polygenic scores, calculated as the weighted sum of trait-associated alleles, to predict genetic effects on a phenotype. Differences in polygenic scores across groups would therefore seem to indicate differences in the genetic basis of the trait, which are of interest to researchers across disciplines. However, because polygenic scores are usually computed using effect sizes estimated using population samples, they are susceptible to confounding due to both the genetic background and the environment. Here, we use theory from population and statistical genetics, together with simulations, to study how environmental and background genetic effects can confound tests for association between polygenic scores and axes of ancestry variation. We then develop a simple method to protect these tests from confounding and show that our approach succeeds in situations that are difficult to protect with standard methods. Our work helps clarify how bias in the distribution of polygenic scores is produced and provides a tool to researchers wishing to protect their analyses from confounding.

## 1. Introduction

The calculation of polygenic scores [1] has become a routine procedure in many areas of human genetics. The promise of polygenic scores is that they provide a means for phenotypic prediction from genotype data alone. By measuring the association between a genetic variant and phenotype in a genome wide association study (GWAS), we obtain an estimate of its effect on the phenotype, averaged over the environments experienced by the individuals in that panel. These effect estimates can then be combined into polygenic scores in a distinct prediction panel by taking a sum of the genotypes of individuals in that panel, weighted by the estimated effects. Under the relatively strict assumptions that genetic and environmental effects combine additively, that variation in the phenotype is not correlated with variation in ancestry the GWAS panel, and that the prediction panel individuals experience a similar distribution of environments to the GWAS panel individuals, these scores can be viewed as an estimate of each individual’s expected phenotype, given their genotypes at the included sites. If these assumptions are met, polygenic scores would seem to provide a means of separating out at least some of the genetic effects on a given phenotype.

However, this promise of polygenic scores is also one of their main pitfalls. The effects of individual variants are typically estimated from population samples in which the environments individuals experience vary as a function of their social, cultural, economic, and political contexts. Differences in these factors are often correlated with differences in ancestry within population samples, and these ancestry-environment correlations induce systematic biases in the estimated effects of individual variants. Similar biases can also arise if genetic effects on the phenotype vary as a function of ancestry within the GWAS panel. Ancestry stratification is a long recognized problem in the GWAS study design [2], and many steps have been taken to guard against its effects. These include bias avoidance approaches, like the sampling of GWAS panels that are relatively homogeneous with respect to ancestry, and statistical bias correction approaches, such as the inclusion of genetic principal components as covariates [3], linear mixed models [4, 5], and LD score regression [6]. While these approaches have largely been successful in minimizing the number of false positive single variant associations [7], effect size estimates can still exhibit slight stratification biases without significantly altering the false discovery rates for individual associations, and these biases can be compounded when aggregating across loci, ultimately leading to confounded predictions in which the ancestry associated effects are mistaken for genetic effects.

Separation of direct genetic effects from correlations between ancestry and environment or genetic background is important to all applications of polygenic scores. Empirically, polygenic scores exhibit geographic clustering even in relatively homogeneous samples and after strict control for population stratification [8, 9, 10, 11]. It is natural to ask if these observed differences reflect a real difference in the average genetic effect on the trait. From a population biology perspective, these patterns may be signals of natural selection [12] or phenotype biased migration [9]. Medically, it is interesting to know if polygenic score differences or gradients represent real underlying gradients in the average genetic effect [13], whether those gradients are caused by non-neutral evolutionary mechanisms or not. However, observed patterns of polygenic scores may also be driven by residual bias in effect size estimates, and stratification biases remain a persistent issue.

This issue has been particularly apparent in the detection of directional selection acting on complex traits. Polygenic scores are an ideal tool for this task, as studying the distribution of scores among individuals who differ in ancestry allows us to aggregate the small changes in allele frequency induced by selection on a polygenic trait into a detectable signal [14, 15, 16, 17]. Several research groups have developed and applied methods to detect these signals [18, 12, 19, 20, 21, 22, 23, 24]. However, these efforts have been met with challenges, as several papers reported signals of recent directional selection on height in Europe using effects obtained from GWAS meta-analyses [25, 26, 18, 12, 27, 28, 29, 20, 30, 31, 19, only for these signals to weaken substantially or disappear entirely when re-evaluated using effects estimated in the larger and more genetically homogeneous UK Biobank [32, 33, 22, 23. Further analysis suggested that much of the original signal could be attributed to spurious correlations between effect size estimates and patterns of frequency variation, presumably induced by uncorrected ancestry stratification in the original GWAS [32, 33].

Recently, in the context of selection tests, Chen et al. 34] proposed a strategy to mitigate the impact of stratification by carefully choosing the GWAS panel so that even if residual stratification biases in effect size estimates exist, they will be unlikely to confound the test (see also [35] for examples of this approach). They reasoned that because polygenic selection tests ask whether polygenic scores are associated with a particular axis of population structure in a given test panel, and because the bias induced by stratification in effect sizes depends on patterns of population structure in the GWAS panel [27], then one should be able to guard against bias in polygenic selection tests by choosing GWAS and test panels where the patterns of population structure within the two panels are not expected to overlap.

However, this approach comes at a cost of reduced power: polygenic scores are generally less accurate when the effect sizes used to compute them are ported to genetically divergent samples [36, 37, 38, 39, 40]. Less accurate polygenic scores are then less able to capture evolution of the mean polygenic score, all else equal [39]. These decays in polygenic score accuracy also pose a significant challenge to their use in medicine, as scores that are predictive for some and not for others may exacerbate health inequities [41]. Thus, realizing the potential of polygenic scores in both basic science and medical applications will require the use of large and genetically diverse GWAS panels. Successfully deploying polygenic scores developed from these diverse panels will require that we have a precise understanding of how bias is produced in polygenic score predictions, and the development of methods to protect against this bias.

In this paper, we develop a general model for bias in polygenic score predictions and propose a simple method to correct for bias in tests for an association between polygenic scores and axes of ancestry variation. We model the covariance of genotypes in a GWAS and test panel in terms of an underlying population genetic model, and write down expressions for the bias in the distribution of polygenic scores as a function of the underlying model. We then show how bias in the association between polygenic scores and a specific axis of ancestry variation in the test panel depends on the extent to which potential confounders in the GWAS lie along a specific axis of ancestry variation in the GWAS panel. Next, we demonstrate that including this particular axis of ancestry variation as a covariate in the GWAS is sufficient to protect against bias in the association between polygenic scores and the chosen axis of ancestry variation in the test panel. We show how to interpret the standard PCA-based approach to controlling for stratification as an effort to capture this particular axis and control for it, and discuss reasons why this approach may fail. Finally, we propose a more direct approach that uses the test panel genotypes to estimate the necessary covariate, and we show using simulations that our approach can succeed in setting where PCA fails.

## 2. Model

### 2.1 Genotypes

To model the distribution of genotypes in both panels, we assume that each individual’s expected genotype at each site can be modelled as a linear combination of contributions from a potentially large number of ancestral populations, which are themselves related via an arbitrary demographic model. Natural selection, genetic drift, and random sampling each independently contribute to the distribution of genotypes across panels, and we make the approximation that these three effects can be combined linearly. We begin by first developing the population level model, before extending it to individuals.

#### 2.1.1. Population model

We assume that for each site ℓ, the vector of allele frequencies across K populations can be decomposed as

(1) $$p_{\ell }=\lambda_{\ell }+\Delta p_{\ell ,D}+\Delta p_{\ell ,S}$$

where $\lambda_{\ell }$ is the allele frequency in the original ancestral population, while the deviations $\Delta p_{\ell ,D}$ and $\Delta p_{\ell ,S}$ capture variation in allele frequency across populations due to genetic drift and natural selection respectively. We approximate the effect of drift via a multivariate Normal model, such that $\Delta p_{\ell ,D}\sim ;MVN\;\left(0,\lambda_{\ell }\left(1-\lambda_{\ell }\right)F_{pop}\right)$, where $F_{pop}$ describes the covariance structure imposed by the population model [42, 43, 44, 45]. Selection induces an additional deviation of $\Delta p_{\ell ,S}=\alpha \beta_{\ell }\lambda_{\ell }\;\left(1-\lambda_{\ell }\right)A_{pop}$, where $\beta_{\ell }$ is site ℓ’s effect on the phenotype of interest, α is the strength of selection on that phenotype, and $A_{pop}$ is a vector recording the extent to which each population inherits from the ancestral population in which selection occurred.

#### 2.1.2. Sampling individuals for GWAS and test panels

Next, we consider taking two samples, one to compose the test panel and one to compose the GWAS panel. Individuals in each panel are created as mixtures of the underlying populations. There are N test panel individuals and the deviation of their genotypes from the expected genotype in the ancestral population $\left(2\lambda_{\ell }\right)$ is

(2) $$X_{\ell }=X_{\ell ,D}+X_{\ell ,S}+X_{\ell ,B},$$

where $X_{\ell ,D}=2W_{X}\Delta p_{\ell ,D},X_{\ell ,S}=2W_{X}\Delta p_{\ell ,S}$. The matrix $W_{X}$ has dimensions N×K, with rows specifying the fraction of ancestry that each of the N test panel individuals inherit from each of the K populations. We can think of the quantity $2\lambda_{\ell }+X_{\ell ,D}+X_{\ell ,S}$ as giving a set of expected genotypes given the evolutionary history of the population, while $X_{\ell ,B}$ contains the binomial sampling deviations across individuals given these expected genotypes.

Similarly, for the M GWAS panels individuals, the deviation of their genotypes can be decomposed as

(3) $$G_{\ell }=G_{\ell ,D}+G_{\ell ,S}+G_{\ell ,B},$$

where $G_{\ell ,D}=2W_{G}\Delta p_{\ell ,D}$, $G_{\ell ,S}=2W_{G}\Delta p_{\ell ,S}$, $W_{G}$ is an M×K matrix with rows specifying the amount of ancestry that each GWAS panel individual inherits from each of the K populations and $G_{\ell ,B}$ captures the binomial sampling variance given the expected genotypes of the GWAS panel individuals (similar to above, $2\lambda_{\ell }+G_{\ell ,D}+G_{\ell ,S}$ specifies this set of individual specific expected genotypes).

#### 2.1.3. Individual level model

Individuals in the two panels can draw ancestry from the same populations, or from related populations, which induces the joint covariance structure

(4) $$Var\left(\left[\begin{array}{l} X_{\ell ,D}\\ G_{\ell ,D} \end{array}\right]\right)=4\lambda_{\ell }\left(1-\lambda_{\ell }\right)F$$

where the matrix

(5) $$F=\left[\begin{array}{ll} F_{XX} & F_{XG}\\ F_{GX} & F_{GG} \end{array}\right]$$

contains the within and between panel relatedness coefficients. These are in turn related to the population level covariance matrix as $F_{XX}=W_{X}F_{pop}W_{X}^{⊤}$, $F_{GG}=W_{G}F_{pop}W_{G}^{⊤}$, and $F_{GX}=F_{XG}^{⊤}=W_{G}F_{pop}W_{X}^{⊤}$. Similarly, the genotypic deviations due to selection can be written at the individual level as $X_{\ell ,S}=2\alpha \beta_{\ell }\lambda_{\ell }\left(1-\lambda_{\ell }\right)A_{X}$ and $G_{\ell ,S}=2\alpha \beta_{\ell }\lambda_{\ell }\left(1-\lambda_{\ell }\right)A_{G}$, where $A_{X}=W_{X}A_{pop}$ and $A_{G}=W_{G}A_{pop}$ describe the extent to which individuals in each of the panels inherit from the selection event. Finally, $X_{\ell ,B}$, $G_{\ell ,B}$, $X_{\ell ,D}$ and $G_{\ell ,D}$ all have mean 0, so the expected genotypes are $E\left[X_{\ell }\right]=X_{\ell ,S}=2\alpha \beta_{\ell }\lambda_{\ell }\left(1-\lambda_{\ell }\right)A_{X}$ and $E\left[G_{\ell }\right]=G_{\ell ,S}=2\alpha \beta_{\ell }\lambda_{\ell }\left(1-\lambda_{\ell }\right)A_{G}$.

### 2.2 Phenotypes

We assume that individuals in the GWAS panel are phenotyped and that the trait includes a contribution from S causal variants, which make additive genetic contributions, as well as an independent environmental effect. The vector of mean-centered phenotypes for the M individuals in the GWAS panel can then be written

(6) $$y=\sum_{\ell }^{S}\beta_{\ell }G_{\ell }+e\;\;=u+e$$

where $u=\sum_{\ell }^{S}\;\beta_{\ell }G_{\ell }$ is the combined genetic effect of all S causal variants, and e represents the combination of all environmental effects.

We assume that the environmental effect on each individual is an independent Normally distributed random variable with variance $\sigma_{e}^{2}$, but that the expected environmental can differ in some arbitrary but unknown way across individuals. We write the distribution of environmental effects as $e\sim ;MVN\;\left(c,\sigma_{e}^{2}I\right)$, where c is the vector of expected environmental effects.

Similar to our decomposition in eq. 3, the genetic effect, u, can be broken down into the contributions from drift, selection, and sampling such that $u=u_{D}+u_{S}+u_{B}$. Here $u_{S}=\sum_{\ell }^{S}\;\beta_{\ell }G_{\ell ,S}$ is the vector of expected values of the genetic contributions to the phenotype, given the ancestries of the individuals in the GWAS panels. Both $u_{D}$ and $u_{B}$ have expectation zero and $u_{D}$ has a correlation structure determined by relatedness matrix $F_{GG}$ (i.e $u_{D}\sim ;MVN\left(0,\;2V_{A}F_{GG}\right)$), where $V_{A}=2\sum_{\ell }^{S}\;\beta_{\ell }^{2}\lambda_{\ell }\left(1-\lambda_{\ell }\right)$ is the additive genetic variance in the original ancestral population. In contrast, the entries of $u_{B}$ are uncorrelated with each other. Therefore $E[u]=u_{S}$ and $u_{S}+c$ gives the vector of individuals’ expected phenotypes, given their ancestry and socio-environmental contexts. We assume that these are not known.

### 2.3. Polygenic scores

We consider a vector of mean centered polygenic scores, computed in the test panel. If the causal effects were known, then the contribution of the S causal sites included in the polygenic score would be

(7) $$Z=\sum_{\ell }^{S}\beta_{\ell }X_{\ell }.$$

Under our genotype model, Z is a vector of random variables with the randomness coming from $X_{\ell ,D}+X_{\ell ,B}$, neutral variation in the test panel genotypes due drift and binomial sampling, respectively. Therefore, the vector of expected polygenic scores for the N individuals in the test panel is

(8) $$\begin{array}{l} E[Z]=\sum_{\ell }^{S}\beta_{\ell }E\left[X_{\ell }\right]\\ \;=\sum_{\ell }^{S}\beta_{\ell }X_{\ell ,S}\\ \;=\alpha V_{A}A_{X}. \end{array}$$

### 2.4 Polygenic score association tests

We want to test the hypothesis that the polygenic scores are associated with some test vector, T, more than is expected due to drift alone. We assume that T is measured only in the test panel, and might represent an eco-geographic variable of interest (e.g latitude [12] or an encoding of whether one lives in a particular geographic region or not [9, 46], the fraction of an individual’s genome assigned to a particular “ancestry group” [18, 20], or one of the top genetic principal components of the test panel genotype matrix [21]). We do not necessarily assume that T is equal to $A_{X}$, the axis along which selection has actually perturbed the polygenic scores.

To test for association of polygenic scores with the test vector, we consider the linear model

(9) Z=qT+ε

where ε is i.i.d. Normal across individuals. A more powerful test is available by modeling the correlation structure among individuals, but the simpler i.i.d. model is sufficient for our purposes (see section S6). We assume that the test vector, T, is scaled to have a variance of one, so the slope is given by

(10) $$q=\frac{1}{N}Z^{⊤}T,$$

and its expectation by,

(11) $$E[q]\;=\frac{1}{N}E[Z{]}^{⊤}T\;\;=\frac{1}{N}\alpha V_{A}A_{X}^{⊤}T$$

Under the null model, α=0, so E[q]=0, reflecting the fact that genetic drift is directionless (though we may also have E[q]=0 if the test vector is not aligned with the axis along which selection has perturbed the polygenic scores, i.e. if $\left.A_{X}^{⊤}T=0\right)$. We compare this null hypothesis to an alternative in which α≠0 (assuming also that $A_{X}^{⊤}T\neq 0$), reflecting the possibility that the polygenic score may have either a positive or negative association with the test vector. We refer to this as a “polygenic score association test”.

We can also re-frame this test as a statement about the association between the effect sizes and a set of genotype contrasts, $r_{\ell }=X_{\ell }^{⊤}T$, which measure the association between the test vector and the genotypes at each site [12]. Similar to the decomposition of the test panel genotypes above, we can decompose the genotype contrasts as

(12) $$r_{\ell }=r_{\ell ,D}+r_{\ell ,S}+r_{\ell ,B},$$

which capture contributions from drift, selection, and sampling, respectively. Writing β and r for the vectors of effect sizes and genotype contrasts across loci, we can write the test statistic as

(13) $$q=\frac{1}{N}\beta^{⊤}r.$$

The equivalence between eq. 10 and eq. 13 will be useful for illuminating several of our results.

Thus far we have assumed that causal effects are known and therefore the only source of randomness in q comes from the genotypes in the test panel. However, in practice effects sizes need to be estimated (see below) and we instead consider polygenic scores computed using marginal effect size estimates, $\overset{ˆ}{Z}=\sum_{\ell }^{S}\;{\overset{ˆ}{\beta }}_{\ell }X_{\ell }$, and use the test statistic

(14) $$\begin{array}{l} \overset{ˆ}{q}\;=\frac{1}{N}{\overset{ˆ}{Z}}^{⊤}T\\ \;=\frac{1}{N}{\overset{ˆ}{\beta }}^{⊤}r, \end{array}$$

which will allow us to characterize the bias, $E[\overset{ˆ}{q}-q]$, in our polygenic score association test.

## 3. Results

Now, given these modeling assumptions, we describe how the relationship between the GWAS and test panels impacts the distribution of polygenic scores and the association between the polygenic scores and the test vector. We first consider the case where no attempt is made to correct for population structure. Motivated by these results, we propose a new correction procedure to remove bias associated with the test vector, and consider several details associated with the implementation of our method.

### 3.1. Estimating marginal effects

Conditional on the genetic and environmental effects, and genotypes at the focal site, the marginal effect size estimate is given by

(15) $${\overset{ˆ}{\beta }}_{\ell }|G_{\ell },u,e\;=\frac{y^{⊤}G_{\ell }}{G_{\ell }^{⊤}G_{\ell }}\;\;=\beta_{\ell }+\frac{u_{-\ell }^{⊤}G_{\ell }}{G_{\ell }^{⊤}G_{\ell }}+\frac{e^{⊤}G_{\ell }}{G_{\ell }^{⊤}G_{\ell }}$$

where we have decomposed the genetic effect into the causal contribution from the focal site and the contribution from the background, i.e. $u=\beta_{\ell }G_{\ell }+u_{-\ell }$. This allows us to further decompose the marginal association in 15 into the causal effect, the association between the focal site and the background genetic contribution from all other sites, and the association with the environment. Under the null hypothesis (i.e. α=0), variation in $G_{\ell }$ is uncorrelated with both $u_{-\ell }$ and e, so an allele’s expected marginal effect is unbiased over evolutionary replicates (see also Robinson et al. (2015) [27] supplement eq. 2.3). Under the alternative hypothesis (α≠0), the expected marginal effect is biased away from zero due to the association between the focal site and expected genetic background and may be biased in either direction depending on the vector of expected environmental effects (see section S1).

### 3.2. Stratification bias induces ancestry associated bias in polygenic scores

The deviation of an allele’s estimated effect size from its expectation depends on $G_{\ell ,D}+G_{\ell ,B}$, the neutral evolutionary and sampling variation around expected genotypes in the GWAS panel, respectively. Because $G_{\ell ,D}$ is correlated with $X_{\ell ,D}$ (deviations due to drift in test panel genotypes) due to shared ancestry, the estimated effect sizes can become correlated with the pattern of genotypic variation in the test panel for reasons that have nothing to do with the actual genetic effect of the variant. This leads to a bias in the expectation of the polygenic scores, and in turn a potential bias in polygenic score association tests. The vector of expected polygenic scores in the test panel is

(16) $$E[\overset{ˆ}{Z}{]}^{⊤}\approx E[Z]+\frac{S}{M}\left(\mu_{S}^{⊤}+c^{⊤}\right){\tilde{F}}_{GX}$$

(see section S2) where $E[Z]=\alpha V_{A}A_{X}$ (see eq. 8), $\mu_{S}=\alpha V_{A}A_{G}$ is the vector expected genetic backgrounds, c is the vector of expected environmental effects, and

(17) $$\begin{array}{l} {\tilde{F}}_{GX}\;=E\left[\frac{G_{\ell ,D}X_{\ell ,D}^{⊤}}{{\left(G_{\ell ,D}+G_{\ell ,B}\right)}^{⊤}\left(G_{\ell ,D}+G_{\ell ,B}\right)/M}\right]\\ \;\approx \frac{F_{GX}}{1\;+\;\overset{-}{F_{G}}}. \end{array}$$

Here $\overset{-}{F_{G}}=\frac{1}{M}\sum_{m=1}^{M}\;F_{mm}$ is the average level of self relatedness in the GWAS panel and ${\tilde{F}}_{GX}$ is the expected cross-panel genetic relatedness matrix computed on standardized genotypes, which is approximately equal to $\frac{F_{GX}}{\left(1+\overset{-}{F_{G}}\right)}$ if $\overset{-}{F_{G}}$ is small.

This result provides explicit theoretical justification for the procedure of choosing GWAS and test panels that do not overlap in population structure as a means to avoiding bias in polygenic score analyses [1, 34, 35], as $E[\overset{ˆ}{Z}-Z]=0$ if ${\tilde{F}}_{XG}=0$. Conditional on a given set of genotypes observed in the GWAS panel, stratification may still bias individual effects, (i.e. $E\left[{\overset{ˆ}{\beta }}_{\ell }|G_{\ell }\right]\approx \beta_{\ell }+\frac{\mu_{S,-\ell }^{⊤}G_{\ell }}{G_{\ell }^{⊤}G_{\ell }}+\frac{c^{⊤}G_{\ell }}{G_{\ell }^{⊤}G_{\ell }}$) but these residual biases are indistinguishable from noise from the perspective of the polygenic scores, as they are uncorrelated with all axes of population structure present in the test panel.

### 3.3. Bias in polygenic scores leads to biased polygenic score associations

The bias in the polygenic score association test statistic $\left(\overset{ˆ}{q}\right)$ follows straightforwardly from the bias in the polygenic scores,

(18) $$E[\overset{ˆ}{q}-q]\approx \frac{S}{NM}\left(\mu_{S}^{⊤}+c^{⊤}\right){\tilde{F}}_{GX}T.$$

Therefore, we expect the polygenic score association test to be biased when the test vector (T) aligns with the vector of expected phenotypes $\left(\mu_{S}+c\right)$ in a space defined by the cross panel genetic similarity matrix $\left({\tilde{F}}_{XG}\right)$. The conditions for an unbiased polygenic score association test are therefore narrower than the conditions needed to ensure unbiased polygenic scores in general. Rather than requiring that ${\tilde{F}}_{XG}=0$, we need only to ensure that certain linear combination of the entries of ${\tilde{F}}_{XG}$ are equal to zero, i.e. that ${\tilde{F}}_{GX}T=0$.

We can gain further intuition by recalling that testing for an association between the polygenic scores and the test vector is equivalent to testing for an association between the effect size estimates and the genotype contrasts (see eq. 13). This allows us to rewrite the bias in $\overset{ˆ}{q}$ as

(19) $$E[\overset{ˆ}{q}-q]\;=\frac{S}{NM}E\left[\left({\overset{ˆ}{\beta }}^{⊤}-\beta^{⊤}\right)r\right]\;\;\approx \frac{S}{NM}\left(\mu_{S}^{⊤}+c^{⊤}\right){\tilde{F}}_{Gr}$$

where

(20) $$\begin{array}{l} {\tilde{F}}_{Gr}\;=E\left[\frac{G_{\ell ,D}r_{\ell ,D}^{⊤}}{{\left(G_{\ell ,D}+G_{\ell ,B}\right)}^{⊤}\left(G_{\ell ,D}+G_{\ell ,B}\right)/M}\right]\\ \;={\tilde{F}}_{GX}T, \end{array}$$

(21) $$\approx \frac{W_{G}F_{pop}T_{pop}}{1\;+\;\overset{-}{F_{G}}},$$

where $F_{pop}T_{pop}$ is a vector of length K, and $T_{pop}=W_{X}^{⊤}T$ expresses the test vector in terms of contrasts between the underlying K populations.

We gain two main insights here. First, eq. 19 expresses the bias entirely in terms of vectors that belong to the GWAS panel: for each GWAS panel individual m, ${\tilde{F}}_{Gr,m}$ measures the covariance between individual m‘s genotype and the genotype contrasts of the test, standardized at each site by the variance of genotypes across individuals in the GWAS panel (eq. 20). Thus, $\overset{ˆ}{q}$ is biased when the vector of expected phenotypes $\left(\mu_{S}+c\right)$ aligns with this vector of standardized covariances $\left({\tilde{F}}_{Gr}\right)$. Confounders which are orthogonal to this axis do not generate bias in the association test, even if they bias the polygenic scores along other axes. Second, eq. 21 shows how the axis captured by ${\tilde{F}}_{Gr}$ in the GWAS panel is related to the underlying populations in our model, and to the pattern of population structure among them. The vector $T_{pop}=W_{X}^{⊤}T$ has length K and captures the axis of the test in terms of the underlying populations, while $F_{pop}T_{pop}$ similarly has length K, and captures the extent to which genetic drift along the path to each population is associated with the axis of population structure identified by the test vector. $F_{pop}T_{pop}$ therefore describes the axis of confounding in terms of populations. Multiplying this vector by $W_{G}$ then rotates this axis into the individual space of the GWAS panel to give ${\tilde{F}}_{Gr}$.

### 3.4. Controlling for stratification bias in polygenic association tests

#### 3.4.1. A simple fixed-effects procedure to remove stratification bias

Given the above results, how can we ensure that patterns we observe in the distribution of polygenic scores are not the result of stratification bias? As discussed above, a conservative solution is to prevent bias by choosing a GWAS panel that does not have any overlap in population structure with the test panel, but this is not ideal due to the well documented portability issues that plague polygenic scores [36, 47, 40], and because it limits which GWAS datasets can be used to test a given hypothesis. Another obvious solution is to include the vectors of expected genetic and environmental effects, $u_{S}$ and c respectively, as covariates in the GWAS. Doing so would remove all ancestry associated bias from the estimated effects, and thus ensure that any polygenic score association test carried out using these effects would be unbiased. However, $u_{S}$ and c are typically not measurable, so this is generally not an option.

Alternately, our analysis above suggests that including ${\tilde{F}}_{Gr}$ as a covariate in the GWAS model will result in an unbiased test no matter what the pattern of confounding is. In this case, the OLS estimated marginal effect at site ℓ, conditional on $G_{\ell }$, u and e, is

(22) $${\overset{ˆ}{\beta }}_{\ell }^{\prime }|G_{\ell },\;u,\;e=\beta_{\ell }+\frac{u_{-\ell }^{⊤}PG_{\ell }}{G_{\ell }^{⊤}PG_{\ell }}+\frac{e^{⊤}PG_{\ell }}{G_{\ell }^{⊤}PG_{\ell }}$$

where $P=\left(I-\frac{1}{∥{\tilde{F}}_{Gr}∥}{\tilde{F}}_{Gr}{\tilde{F}}_{Gr}^{⊤}\right)$ prevents variation along the axis specified by ${\tilde{F}}_{Gr}$ from being used in the estimation of effects, and renders them uncorrelated with the genotype contrasts r under the null. If there is confounding along other shared axes of ancestry variation, the polygenic scores may still be biased along other axes, as

(23) $$E[\overset{ˆ}{Z}{]}^{⊤}\approx E[Z]+\frac{S}{M}\left(\mu_{S}^{⊤}+c^{⊤}\right){\tilde{F}}_{GX}^{⊥{\tilde{F}}_{Gr}}$$

where

(24) $${\tilde{F}}_{GX}^{⊥{\tilde{F}}_{Gr}}\approx P{\tilde{F}}_{GX}$$

captures cross panel relatedness along all axes of variation other than that specified by ${\tilde{F}}_{Gr}$. However, controlling for variation aligned with ${\tilde{F}}_{Gr}$ ensures that ${\tilde{F}}_{GX}^{⊥{\tilde{F}}_{Gr}}T=0$, and it follows that

(25) $$\begin{array}{l} E[\overset{ˆ}{q}-q]\;\approx \frac{S}{NM}\left(\mu_{S}^{⊤}+c^{⊤}\right){\tilde{F}}_{GX}^{⊥{\tilde{F}}_{Gr}}T\\ \;\approx 0 \end{array}$$

and the polygenic score association test is unbiased (see S4 and S5).

#### 3.4.2. Relationship to PCA

A standard approach to controlling for population stratification in polygenic scores is to include the top J principle components of the GWAS panel genotype matrix as covariates in the GWAS, for some suitably large value of J [3]. How does our approach relate to the this standard approach?

**Figure 1:**
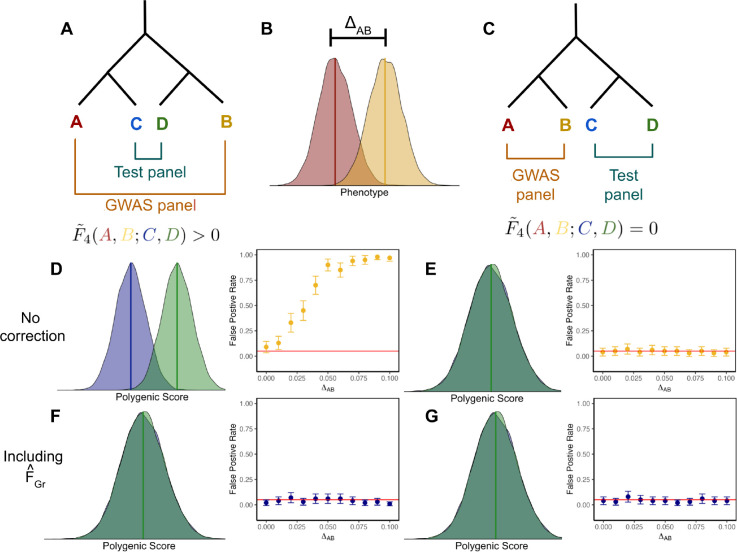
Schematic of two different panel configurations. The effect of stratification depends on the overlapping structure between the GWAS and test panels. (A, C) Two different topologies used to create the GWAS and test panels. (B) Stratification was modeled in the GWAS panel by drawing an individual’s phenotype y~;N(0,1) and adding $\Delta_{AB}$ if they originated from population B. (D) When there is overlapping structure between GWAS and test panels, there is an expected mean difference between polygenic scores in populations C and D. Additionally, the false positive rate for $\overset{ˆ}{q}$ increases with the magnitude of stratification in the GWAS. (E) However, when there is no overlapping structure between panels, there is no expected difference in mean polygenic scores between C and D and the false positive rate remains near 5% regardless of the magnitude of stratification. (F, G) Including ${\overset{ˆ}{F}}_{Gr}$ as a covariate in the GWAS controls for stratification, keeping the false positive rate at nominal levels regardless of $\Delta_{AB}$ or the overlapping structure between GWAS and test panels. False positive rates were calculated as the number of simulations with p<0.05 out of 100 simulations for each $\Delta_{AB}$.

In our model, $F_{GG}$ contains the within panel relatedness for the individuals in the GWAS panel, where the entries themselves are a linear combination of the population level covariance matrix, $F_{pop}$, the structure of which is determined by the demographic model. If we could take the eigendecomposition of $F_{GG}$ directly, then the number of PCs that correspond to structure is entirely dependent on the population model. For example, in section 3.5.1 we simulate under a 4 population sequential split model (Figure 1), in which case there are three PCs that reflect real underlying structure. In section 3.5.2 we use a symmetric equilibrium migration model on a six-by-six lattice grid (Figure 3), in which case there are 35 PCs reflecting underlying population structure. Including these PCs is sufficient to remove all ancestry-associated bias in effect sizes estimates and render polygenic scores uncorrelated with any axis of ancestry variation under the null hypothesis.

To see how our approach relates to the PCA correction approach, we can write ${\tilde{F}}_{Gr}$ as a linear combination of GWAS panel PCs,

(26) $${\tilde{F}}_{Gr}=\sum_{i}\;\eta_{i}U_{i}$$

where $U_{i}$ is the *i*^*th*^ PC of $F_{GG}$ and the weights are given by $\eta_{i}=Cov\left(U_{i},{\tilde{F}}_{Gr}\right)$. Estimating the marginal associations with ${\tilde{F}}_{Gr}$ as a covariate can therefore be understood as fitting a model in which all PCs of the GWAS panel genotype matrix are included as covariates, but the relative magnitude of the contributions from different PCs are fixed, and we estimate only a single slope that scales the contributions from all of the PCs jointly, i.e.

(27) $$y=G_{\ell }\beta_{\ell }+\left(\sum_{i}\eta_{i}U_{i}\right)\omega +e.$$

As a corollary, if we perform a polygenic score association test using GWAS effect size estimates in which the top J PCs of $F_{GG}$ are included as covariates, the necessary and sufficient condition for the included PCs to protect against bias from unmeasured confounders in a particular polygenic score association test is that ${\tilde{F}}_{Gr}$ is captured by those J top PCs, i.e. that $\eta_{i}\approx 0$ for i>J.

In practice, however, we do not directly observe $F_{GG}$ (and therefore cannot directly observe the PCs of the underlying data generating process, U). Rather, we observe a finite sample, and must estimate these quantities from the sample genotype data [3]. The accuracy of these “sample” PCs, $\overset{ˆ}{U}$, as compared to U, depend on the level of structure in the GWAS panel, as well as the number of individuals and SNPs. Specifically, as shown first by Patterson et al. (2006) in the context of genetics [48], PCA exhibits a phase change behavior in which a given principal component will only be detectable if the corresponding eigenvalue is greater than a threshold value that depends on the ratio of the number of SNPs to the number of individuals. Perhaps less well appreciated however is the fact that even when the corresponding eigenvalue exceeds this threshold, the angle between the sample PC and the true PC of the underlying population model may still be substantially less than one, particularly if the relevant eigenvalue does not far exceed the detection threshold [49, 50]. Thus, when PCA is applied to smaller GWAS samples, either of these two related phenomena may make it difficult to accurately approximate ${\tilde{F}}_{Gr}$ as a linear combination of the top sample PCs, leading to a failure to fully account for stratification bias in polygenic score association tests.

A second interpretation of the PC correction approach is that it operates on a hypothesis that the major axes of confounding in a given GWAS panel (i.e. $\mu_{S}+c$ in our notation) can be captured by the included PCs [51]. If this condition is met, effect size estimates are unbiased with respect to all axes of ancestry variation and therefore any association test that utilizes them will be unbiased as well. Combining this interpretation with results from above, PCA should successfully eliminate bias in polygenic score association tests if the J sample PCs included in the GWAS either capture the confounding effects on the phenotype, eliminating all effect size bias, or capture ${\tilde{F}}_{Gr}$, ensuring that effect size bias relevant to the test is removed.

#### 3.4.3. Implementing the bias correction

Given these difficulties with the PCA approach, we propose an alternative, which is to obtain a direct estimate of ${\tilde{F}}_{Gr}$ using the relevant test panel genotype contrasts. Given the test panel genotype contrasts and GWAS panel genotypes from L sites genome-wide we obtain an estimate, ${\overset{ˆ}{F}}_{Gr}$, as

(28) $${\hat{F}}_{Gr}=\frac{1}{L}\sum_{\ell =1}^{L}\frac{G_{\ell }r_{\ell }}{G_{\ell }^{⊤}G_{\ell }/M}.$$

We then estimate marginal effects in the GWAS under the model

(29) $$y=G_{\ell }\beta_{\ell }+{\overset{ˆ}{F}}_{Gr}\omega +\varepsilon ,$$

and ascertain SNPs for inclusion in the polygenic scores via standard methods.

#### 7.4.4. Predicting GWAS panel genotypes

Above, we derived our proposed method in the context of a population genetic model. The form of eq. 28 suggests another useful interpretation, that of controlling for a prediction of the GWAS panel genotypes. To see this, suppose first that we standardize the GWAS panel genotypes by their variance so that ${\tilde{G}}_{\ell }=\frac{G_{\ell }}{\sqrt{G_{\ell }^{⊤}G_{\ell }/M}}$ and we similarly standardize the test panel contrasts so that ${\tilde{r}}_{\ell }=H\frac{r_{\ell }}{\sqrt{G_{\ell }^{⊤}G_{\ell }/M}}$, where H is a constant shared across loci, and chosen so that ${\tilde{r}}^{⊤}\tilde{r}=L$. Now, consider the matrix regression model

(30) $$\tilde{G}=\rho {\tilde{r}}^{⊤}+\varepsilon .$$

In this model, $\tilde{G}$ is an M×L matrix containing the standardized genotypes $\left({\tilde{G}}_{\ell }\right)$ as columns, ρ is a vector of length M containing one regression coefficient per individual in the GWAS panel, $\tilde{r}$ is the length L vector of standardized genotype contrasts, and ε is an M×L matrix of residuals. The OLS estimate of ρ is

(31) $$\hat{\rho }=\frac{1}{L}\sum_{\ell =1}^{L}\frac{G_{\ell }r_{\ell }}{G_{\ell }^{⊤}G_{\ell }/M},$$

identical to the expression for ${\overset{ˆ}{F}}_{Gr}$ in eq. 28 (i.e. $\overset{ˆ}{\rho }={\overset{ˆ}{F}}_{Gr}$). Thus, for each site ℓ, a linear prediction of ${\tilde{G}}_{\ell }$ given ${\tilde{r}}_{\ell }$ is equal to the product of $\overset{ˆ}{\rho }$ (i.e. ${\overset{ˆ}{F}}_{Gr}$) and ${\tilde{r}}_{\ell }$:

(32) $$E\left[{\tilde{G}}_{\ell }|{\tilde{r}}_{\ell }\right]=\overset{ˆ}{\rho }{\tilde{r}}_{\ell }.$$

Therefore, we can intuitively interpret our bias correction method as including a prediction of the GWAS panel genotypes given the test panel genotype contrast as a covariate when estimating the effect at site ℓ.

This formulation also helps to highlight why our approach can succeed in settings where PCA fails (a point we demonstrate further in simulations below). Both our predictor $\left(\tilde{r}\right)$ and our responses $\left({\tilde{G}}_{\ell }\right)$ are scaled to have a variance of one, so the entries of ${\overset{ˆ}{F}}_{Gr}$ vary around their true values with a standard deviation of approximately $L^{-1/2}$. The error in ${\overset{ˆ}{F}}_{Gr}$ therefore does not depend on the size of the GWAS panel, in contrast to PCA. Including ${\overset{ˆ}{F}}_{Gr}$ as a covariate when estimating effects should therefore provide protection against false associations between polygenic scores and ancestry so long as the entries of ${\tilde{F}}_{Gr}$ vary on a scale that is significantly greater than the scale of this estimation error, i.e. if $\sqrt{\frac{1}{M}\sum_{m}\;{\tilde{F}}_{Gr,m}^{2}}\gg L^{-1/2}$. Intuitively, it is easier to protect against stratification bias along a given axis of population structure if we specify that axis directly (even if we do so in a different panel) than if we use an unsupervised approach such as PCA to discover and estimate several axes of population structure and hope that the relevant axis is captured by these estimates.

#### 3.4.5. Downward bias with true signal

Our approach proposes to use the test panel genotype data twice: once when controlling for stratification in the GWAS panel, and a second time when testing for an association between the polygenic scores and the test vector. Shouldn’t this remove the signal we are trying to detect? While the answer is yes, at least for naive applications, the effect will be small so long as the number of SNPs used to compute the correction is large relative to the number included in the polygenic score.

To see why, we can rewrite the regression eq. 29 in terms of a sum of the contribution from our focal site and all other sites as

(33) $$y=G_{\ell }\beta_{\ell }+\left(\frac{L\;-\;1}{L}{\overset{ˆ}{F}}_{Gr,\ell }+\frac{1}{L}{\tilde{G}}_{\ell }{\tilde{r}}_{\ell }\right)\omega +e$$

where ${\overset{ˆ}{F}}_{Gr,-\ell }$ is the estimate of ${\tilde{F}}_{Gr}$ that one would obtain using all sites other than site ℓ. Thus, because our ${\overset{ˆ}{F}}_{Gr}$ includes a contribution from the focal site, controlling for it induces a slight bias in the estimated effect size, the sign of which depends on the sign of ${\tilde{r}}_{\ell }$. If ${\tilde{r}}_{\ell }$ is positive, ${\overset{ˆ}{\beta }}_{\ell }$ has a slight negative bias, whereas if ${\tilde{r}}_{\ell }$ is negative, the bias will be positive. Similar effects are noted elsewhere in the statistical genetics literature, for example in correcting for PCs of gene expression data [52], and linear mixed models [53], where it has been termed “proximal contamination”. Assuming that the variance of ${\tilde{r}}_{\ell }$ across sites included in the score is similar to those used to compute the correction, the product $r_{\ell }{\overset{ˆ}{\beta }}_{\ell }$ will be biased toward 0 by a factor of approximately $\left(1-\frac{1}{L}\right)$ for each site, owing to the fact that the focal site contributes approximately $\frac{1}{L}$ of our estimate ${\overset{ˆ}{F}}_{Gr}$. Our test statistic is a sum over contributions of $r_{\ell }{\overset{ˆ}{\beta }}_{\ell }$ from S independent sites, so including ${\overset{ˆ}{F}}_{Gr}$ as a covariate when estimating effect sizes induces a downward bias of approximately $\left(1-\frac{S}{L}\right)$, i.e.

(34) $$E[\overset{ˆ}{q}|q]\approx q\left(1-\frac{S}{L}\right).$$

Notably, controlling for PCs of the GWAS panel genotype matrix will induce a similar effect if the axis of structure corresponding to the test is also present in the GWAS panel and gets included in the GWAS model as a covariate. In either case, the downward bias should be small so long as sites used to compute the polygenic score are only a small subset of those used to estimate ${\overset{ˆ}{F}}_{Gr}$. While this picture is somewhat complicated by the existence of linkage disequilibrium in real populations, for human population samples imputed to common reference panels, the effective number of SNPs is typically on the order of at least half a million [54], suggesting that even for a polygenic score that included, for example, 10,000 SNPs, the downward bias should be no more than 2%. An important caveat is that some methods for computing polygenic scores allow for all or at least a substantial fraction of all SNPs genome wide to make non-zero contributions to the polygenic score. Further concern about downward biases in applications could likely be ameliorated via the “leave one chromosome out” scheme commonly implemented in the context of linear mixed models [53, 5] or via iterative approaches that first aim to ascertain SNPs using a genome-wide estimate of ${\overset{ˆ}{F}}_{Gr}$ before re-estimating effects using an estimate of ${\overset{ˆ}{F}}_{Gr}$ computed from sites not in strong LD with any of the ascertained sites. Understanding the behavior of tests for polygenic score-ancestry associations in the context of these methods will require careful attention to the methods’ assumptions.

### 3.5. Applications: Theory and Simulations

In this section, using both theory and simulations, we consider a number of concrete examples with varying degree of alignment between the axis of stratification and axis of population structure relevant to the polygenic association test, demonstrating how these biases play out in practice, and how our proposed correction reduces false positives to nominal levels.

#### 3.5.1. Toy Model

We first consider a toy model with four populations (labeled A, B, C and D), which are related to one another by an evenly balanced population phylogeny (Figure 1). The GWAS panel is composed of an equal mixture of individuals from populations A and B, and we test for a difference in mean polygenic score between populations C and D under two different topologies, one where A and C are sister to one another (Figure 1A), and another where A and B are sister (Figure 1 C).

For simplicity, we consider a purely environmental phenotype (i.e. $h^{2}=0$) with a difference in mean between populations A and B equal to $\Delta_{AB}$, so that $e_{i}=\frac{\Delta_{AB}}{2}+\varepsilon_{i}$ if individual i belongs to population A, and $e_{i}=-\frac{\Delta_{AB}}{2}+\varepsilon_{i}$ if they belong to population B, where $\varepsilon_{i}\sim ;N(0,\;1)$ (Figure 1B). Following from eq. 15, the marginal effect size estimate for site ℓ is

(35) $${\overset{ˆ}{\beta }}_{\ell }|G_{\ell },e\;=\frac{G_{\ell }^{⊤}e}{G_{\ell }^{⊤}G_{\ell }}\;\;=\frac{1}{2}\frac{\Delta_{AB}\left({\overset{ˆ}{p}}_{A,\ell }\;-\;{\overset{ˆ}{p}}_{B,\ell }\right)}{G_{\ell }^{⊤}G_{\ell }/M}+\frac{G_{\ell }^{⊤}\varepsilon }{G_{\ell }^{⊤}G_{\ell }}$$

where ${\overset{ˆ}{p}}_{A,\ell }$ and ${\overset{ˆ}{p}}_{B,\ell }$ are the observed sample allele frequencies for population A and B at site ℓ (see also equation 2.3 in the supplement of [27]).

Then, using these effect sizes to test for a difference in polygenic score between populations C and D, the bias in our association test statistic is,

(36) $$E[\hat{q}-q]=\Delta_{AB}\sum_{\ell =1}^{S}E\left[\frac{\left({\hat{p}}_{A,\ell }\;-\;{\hat{p}}_{B,\ell }\right)\left({\hat{p}}_{C,\ell }\;-\;{\hat{p}}_{D,\ell }\right)}{G_{\ell }^{⊤}G_{\ell }/M}\right]\;\;=\Delta_{AB}S{\tilde{F}}_{4}(A,B;C,D)$$

where ${\tilde{F}}_{4}(A,B;C,D)$ is a version of Patterson’s $F_{4}$ statistic [55, 56], standardized by the genotypic variance in the GWAS panel, which measures the amount of genetic drift common to population A and B that is also shared by populations C and D. Writing the bias in terms of this modified $F_{4}$ statistic helps illustrate the role of cross panel population structure in driving stratification bias in polygenic scores. The effect estimate at site ℓ is a linear function of ${\overset{ˆ}{p}}_{A,\ell }-{\overset{ˆ}{p}}_{B,\ell }$, so the test will be biased if ${\overset{ˆ}{p}}_{A,\ell }-{\overset{ˆ}{p}}_{B,\ell }$ is correlated with ${\overset{ˆ}{p}}_{C,\ell }-{\overset{ˆ}{p}}_{D,\ell }$. This is true for the demographic model in Figure 1A, where shared drift on the internal branch generates such a correlation, yielding a positive value for ${\tilde{F}}_{4}(A,B;C,D)$, but not for the model in Figure 1B, where there is no shared internal branch, ${\tilde{F}}_{4}(A,B;C,D)=0$.

To test this prediction, we simulated 100 replicates of four populations related by this topology where the $F_{ST}$ between pairs of populations on the same side of the tree is approximately 0.005 and approximately 0.01 for populations on opposite sides. We then simulated purely environmental phenotypes in the GWAS panel populations (A and B), with a difference in mean phenotype (as outlined above) conducted a GWAS, ascertained SNPs, and then used these SNPs to construct polygenic scores and test for a difference in mean polygenic scores between populations C and D. To assess whether the difference was significantly different from zero, we compare to an empirical null distribution constructed by permuting the signs of the of the effect estimates across sites (see Methods). The results are consistent with our theoretical expectations: the test is biased for the topology with ${\tilde{F}}_{4}(A,B;C,D)>0$ (Figure 1D), but unbiased when ${\tilde{F}}_{4}(A,B;C,D)=0$ (Figure 1E).

Given the population model, ${\tilde{F}}_{XG}=0$ for the unconfounded topology, making ${\tilde{F}}_{Gr}$ is a vector of zeros. For the confounded topology, the structure in ${\tilde{F}}_{XG}$ reflects the deepest split in the phylogeny and is aligned with T. ${\tilde{F}}_{Gr}$ is therefore an indicator of which GWAS panel individuals are on which side of the deepest split. In our simulations we estimate ${\overset{ˆ}{F}}_{Gr}$, as in eq. 28, using all genome-wide SNPs with a frequency of greater than 5% in both the test and GWAS panels. The resulting vector is, as expected, tightly correlated with population label for the topology with ${\tilde{F}}_{4}(A,B;C,D)>0$, but reflects random noise for the case when ${\tilde{F}}_{4}(A,B;C,D)=0$ (Figure S2). When we re-run the polygenic score association test using effects estimated with ${\overset{ˆ}{F}}_{Gr}$ included as a covariate in the GWAS, the bias in $\overset{ˆ}{q}$ is removed (Figure S1) and false positive rates are restored to nominal levels for the confounded topology (Figure 1F). For the unconfounded topology, the test is already unbiased, so the correction has no effect (Figure 1G).

Next, we wanted to confirm that including ${\overset{ˆ}{F}}_{Gr}$ does not regress out true signals of polygenic score divergence, consistent with our theoretical argument above. To do this, we modified our confounded topology simulations by adding causal loci to make the trait heritable, with $h^{2}=0.3$, and sampled the sign of the effect for these causal loci to generate a correlation between the effect and the frequency differences between populations C and D. This procedure generates an $A_{pop}$ with non-zero and opposite sign entries for populations C and D and is conceptually equivalent adding a selection event on the internal branch in the population phylogeny. We then compare the true positive rate across simulations under three different schemes for obtaining effect sizes: 1) estimating effects with no bias correction, 2) including ${\overset{ˆ}{F}}_{Gr}$ as a covariate, or 3) including known population ID in the GWAS. Correcting for population ID is included as a gold standard for this simple model, but would be unrealistic for real samples that do not conform to idealized population models. For each scheme, we consider both the case where the identity of the causal variants are known but the effects are estimated vs ascertaining the most significant variant per LD block using p-values. We compare all of these schemes to association tests performed using the true causal variants with their true causal effect sizes.

**Figure 2:**
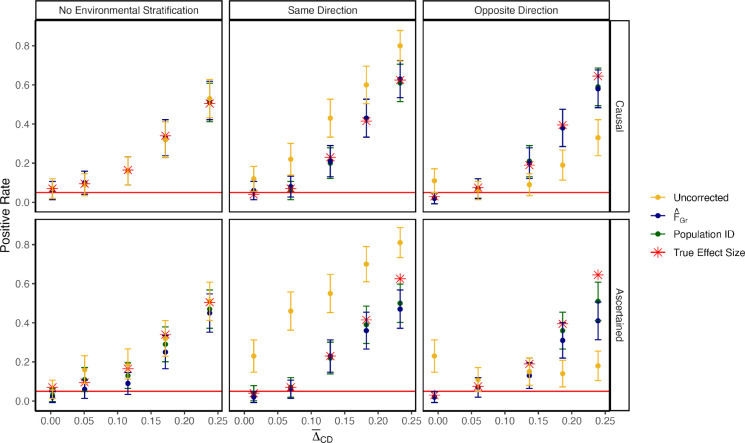
Including ${\overset{ˆ}{F}}_{Gr}$ as a covariate in the GWAS model does not regress out true signal. Here we generate a true difference in average polygenic scores by flipping the sign of a proportion of causal effect sizes to align with allele frequency contrasts, $p_{D,\ell }-p_{C,\ell }$, in the test panel. ${\overset{‾}{\Delta }}_{CD}$ is the true difference in polygenic scores averaged across 100 replicates. The top row is the positive rate in our association test using estimated effect sizes at causal sites. Regardless of the direction of the stratification effect, our correction procedure perfectly captures the real signal (computed using the true effect sizes). The bottom row is the positive rate when polygenic scores were computed using sites ascertained on p-value. While there is some loss of power due to imperfect tagging, our approach maintains power to detect true signal.

When the identities of the causal sites are known and there is no environmental stratification, all three schemes for estimating effects perform similarly, and equally as well as when the causal effects themselves are known exactly (Figure 2, top left). As expected, when the environmental stratification is in the same direction as the true mean difference, the uncorrected effect sizes reject the null at a higher rate than when the causal effects are known, while our method and population ID perform as well as having the causal effects (Figure 2, top middle). The same is true when the environmental effect is in the opposite direction as the true signal, except the uncorrected effects have reduced power, as expected (Figure 2, top right). When the identities of the causal variants are unknown and associated sites must be ascertained, we observe largely similar patterns, with our method and the population ID method having slightly reduced power relative to when the identities of the causal variants are known (Figure 2, bottom row). Our simulation results support our theoretical argument that including a genome-wide estimate of ${\overset{ˆ}{F}}_{Gr}$ will have a minimal effect on power to detect real signals as long as S<<L.

#### 3.5.2. Grid Simulations

To further explore our approach in more complex scenarios, we conducted another set of coalescent simulations under a symmetric two-way migration model on a six-by-six lattice grid, building off of a framework developed by Zaidi and Mathieson (2020) [57]. We sampled an equal number of individuals per deme to comprise both the GWAS and test panels and then simulate several different distributions of purely environmental phenotypes across the GWAS panel individuals. We consider three different scenarios for the distribution of phenotypes: one where the mean varies with latitude, one where it increases along the diagonal of the grid, and one in which only a single deme has an elevated mean, a scenario which previous work has shown to be difficult to correct for with standard tools [58, 57]. For each scenario, we estimate effect sizes, ascertain associated sites, and test for an association between polygenic score and latitude, longitude, or membership in the single confounded deme, depending on the example.

**Figure 3:**
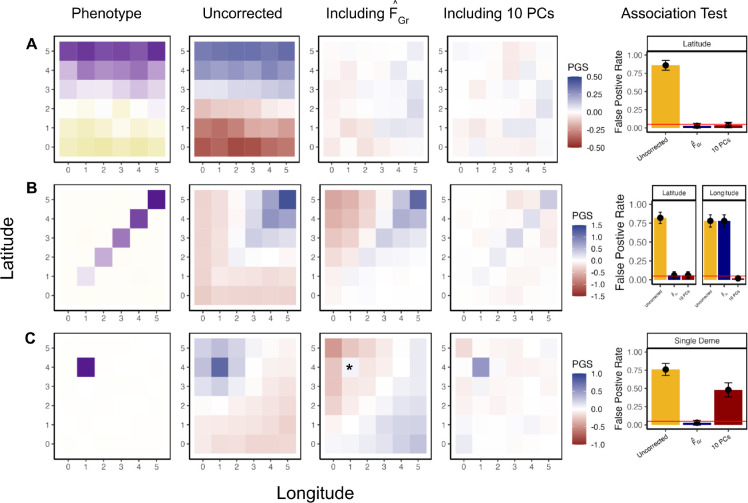
Including ${\overset{ˆ}{F}}_{Gr}$ as a covariate in the GWAS model controls for stratification bias specific to the association test of interest. GWAS and test panel individuals were simulated using a stepping-stone model with continuous migration. In the GWAS panel, the phenotype is non-heritable and stratified along either latitude (A), the diagonal (B), or in a single deme (C). When effect sizes are estimated in a GWAS with no correction for stratification, polygenic scores constructed in the test panel recapitulate the spatial distribution of the confounder (second column). Including ${\overset{ˆ}{F}}_{Gr}$ (test vector is latitude for A and B, belonging to * deme for C) in the GWAS model eliminates bias in polygenic scores along the test axis (third column) and the false positive rate for $\overset{ˆ}{q}$ across 100 replicates remains at nominal levels (fifth column). We also compare our approach to including the top 10 PCs (fourth column). For (A) the magnitude of confounding Δ is 0.2 standard deviations while Δ=1 for (B) and (C) (see Methods).

For the first example, the confounder, c, is a linear function of an individual’s position on the latitudinal axis (Figure 3A). When we estimate effect sizes with no correction for population structure, the spatial distribution of the resulting polygenic scores reflects the distribution of the environmental confounder. Consequently, an association test using latitude as the test vector is biased. However, when we compute ${\overset{ˆ}{F}}_{Gr}$ and include it as a covariate in the GWAS model, effect sizes are unbiased with respect to the latitudinal genotype contrasts in the test panel and the resulting association test is unbiased.

In the second example, we simulate confounding along the diagonal, resulting in uncorrected polygenic scores that are correlated with both latitude and longitude in the test panel and an association test that is biased along both axes (Figure 3B). When we compute ${\overset{ˆ}{F}}_{Gr}$ using latitude as the test vector, the resulting effect sizes are uncorrelated with latitudinal genotype contrasts, but remain susceptible to bias along other axes (e.g. longitude). This example highlights the targeted nature of this method, as using effect sizes from a GWAS including ${\overset{ˆ}{F}}_{Gr}$ does not remove all bias, but does make the association test using those effect sizes for the pre-specified test vector unbiased.

**Figure 4:**
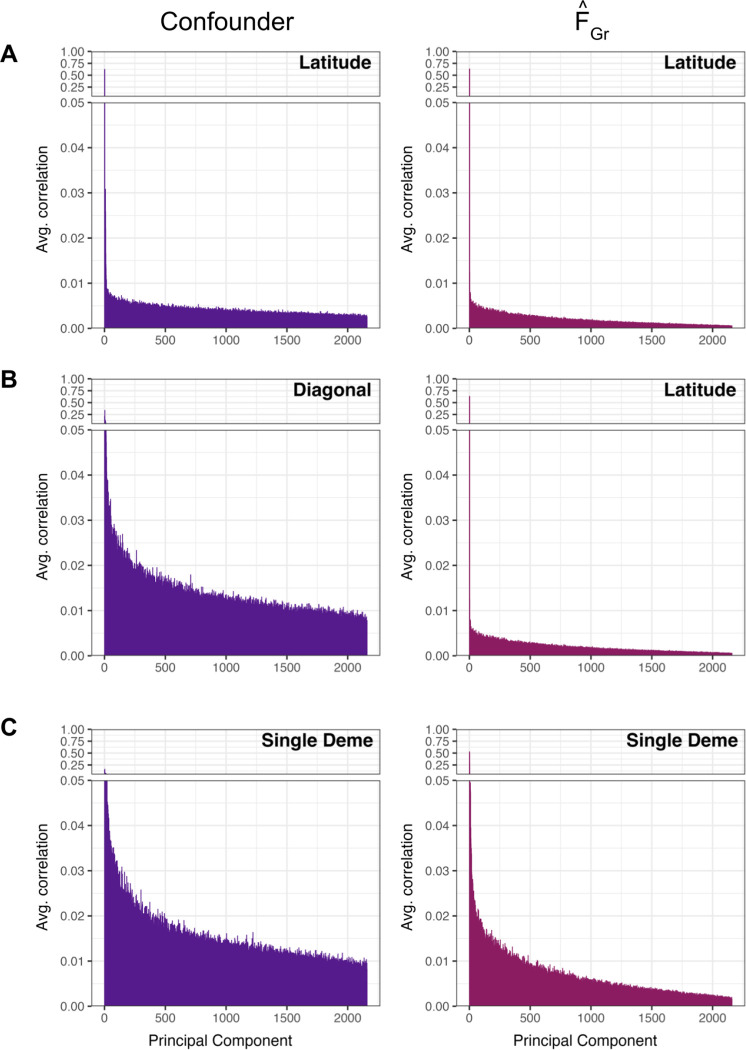
Different patterns of confounding and ${\overset{ˆ}{F}}_{Gr}$ load onto different GWAS panel principal components. For the three possible combinations of confounding and polygenic score association tests in Figure 3, we plot the average correlation across replicates between the confounder, ${\overset{ˆ}{F}}_{Gr}$, and the GWAS panel principal components. To better visualize differences, we cut the y-axis at 0.05 using 60] and enlarge the bottom portion of the plot. In (A) both the confounder and ${\overset{ˆ}{F}}_{Gr}$ represent variation along latitude and are well captured by the first two PCs. For (B) the confounder varies along the diagonal and these individual deme level differences are correlated with higher PCs. In contrast, the test vector is still latitude and ${\overset{ˆ}{F}}_{Gr}$ is again well captured by PCs 1 and 2 . Finally, in (C), both the confounder and the test vector represent membership in a single deme and therefore the confounder and ${\overset{ˆ}{F}}_{Gr}$ are correlated with higher PCs that either capture finer structure in the sample or represent random orthogonal vectors.

In the first two examples, the standard approach of including the top *J* = 10 principal components of the GWAS panel genotype matrix as covariates in the GWAS successfully control for confounding along the latitudinal axis, albeit for potentially different reasons. As outlined in Section 3.4.2, PCA is successful when either the confounder or ${\tilde{F}}_{Gr}$ falls in the span of the sample PCs included in the GWAS. In Figure 4 we compute the average correlation between all M-1 PCs and either the confounder or our estimate ${\overset{ˆ}{F}}_{Gr}$. For the first example, both the confounder and ${\overset{ˆ}{F}}_{Gr}$ reflect broad geographic structure and are easily captured by the first two PCs. In the second example, however, the confounder lies along a more complicated geographic axis, which is not fully captured by the top 10 PCs (Figure 4B, left column). As a result, the polygenic scores retain some amount of confounding along the diagonal (Figure 3B, fourth column). However, because of the simple relationship between geography and migration patterns in our simulations and the overlapping structure between the two panels, the axis of structure relevant for the latitudinal association test in the test panel is the latitudinal axis in the GWAS panel (Figure S5). This pattern is well described by the top 10 PCs (Figure 4B, right column), so PCA protects the test in this scenario despite the residual confounding. The same is true for the test of association with longitude.

In the third example, we simulate an increased environmental effect in a single deme, which induces a more complex spatial pattern in the uncorrected polygenic scores (Figure 3C). We then take the test vector to be an indicator for whether the test panel individuals were sampled from the deme with the environmental effect or not, and compute ${\overset{ˆ}{F}}_{Gr}$ using these contrasts. In this case, the spatial distribution of ${\overset{ˆ}{F}}_{Gr}$ in the GWAS panel is more complicated, reflecting the genetic distance of each deme from the focal deme of the test, given the equilibrium migration model (Figure S5). Including only ${\overset{ˆ}{F}}_{Gr}$ in the GWAS protects the test from confounding, but in doing so induces a residual pattern in the polygenic scores that is largely a mirror image of the original, uncorrected pattern of confounding. In contrast, including the top 10 PCs reduces much of the variation in the polygenic scores across the landscape, but is insufficient to protect the association test. Combining both approaches protects the test without inducing a residual pattern in the polygenic scores (Figure S6). We revisit this observation in the Section 4.

Here, PCA alone fails because neither the confounder nor ${\tilde{F}}_{Gr}$ are fully captured by the included PCs (Figure 4C). In fact, for this example we had to include 1000 PCs to reduce false positive rates to nominal level (Figure S3), and doing so regressed out 75% of all available genetic variation. This may initially be surprising: as there are 36 demes, we should need only 35 linear axes to explain any given pattern of variation across these demes. At least in principle, we could compute the expected pairwise coalescent times for individuals sampled from different demes, and use these to compute the expected genetic relationship matrix given the demographic model. Then, taking the eigendecomposition of this matrix would yield 35 axes with non-zero eigenvalues [59], and it would be possible to capture any confounder/protect any test by controlling for these 35 PCs. However, as we discussed above, PCs computed from the sample genotype data can be poor estimates of the PCs of the underlying population process, and below a certain threshold, may not be detectable at all [48, 49]. In this setting, adding additional sample PCs merely amounts to controlling for additional random orthogonal vectors. While a sufficiently large number of random vectors is ultimately able to capture the confounder/test well enough to control the false positive rate, this comes at the cost of also regressing out most of the variation we would like to use to estimate effect sizes, and so is not a viable option.

Finally, we tested our approach for each of these for scenarios over a range of magnitudes for the confounding effect. In our simulations, ${\overset{ˆ}{F}}_{Gr}$ protects nearly all association tests from confounding effects of up to two phenotypic standard deviations, whereas the top 10 principle components begin to fail in some scenarios for more modest strengths of the confounding effect (Figure S4).

## 4. Discussion

Interpreting patterns in the distribution of polygenic scores is difficult, especially when confounding cannot be ruled out. Because most well-powered GWAS are conducted on population samples where the relationship between genetic background, ancestry, and the environment is not well controlled, stratification bias remains a significant concern [32, 33, 40, 61]. Here, we characterize patterns of stratification bias in the distribution of polygenic scores as a function of the genetic similarity between GWAS and test panels, and show how test panel genotypes can be used to protect against stratification bias when testing hypotheses about the relationship between polygenic scores and ancestry. By using the test panel and GWAS panel genotypes jointly, this approach removes guesswork in choosing the number of top PCs to include in the GWAS and can succeed in situations where PCA fails.

Another advantage of our approach is related to the tradeoff between statistical power and robustness in polygenic selection analyses. Researchers motivated by the difficulties with stratification bias and height [32, 33] have taken conservative approaches meant to trade away statistical power in exchange for robustness to biases due to ancestry stratification. This includes the non-overlapping population structure study design [34, 35, 62] or using effect sizes from smaller family-based GWASs [35, 63], but also choices such as focusing only on genome wide significant hits, or throwing away all effect size information except for the sign [35, 18]. Our approach provides a stronger guarantee against bias due to ancestry stratification which will allow researchers to use less conservative methods for computing polygenic scores that do not trade away so much statistical power.

Our results are also relevant for analyses that use GWAS summary statistics and coalescent methods to test for signals of directional polygenic selection [19, 23, 24, 64]. One way of thinking about these coalescent approaches is to recognize that they use patterns of haplotype variation to estimate genotype contrasts between the sampled present day individuals and a set of unobserved ancestors, and then ask whether these estimated genotype contrasts correlate with effect size estimates for a trait of interest. Thus, if the relevant genotype contrasts are estimated using the coalescent methods before conducting the GWAS, then these estimated genotype contrasts could be used to immunize the coalescent analysis against stratification bias, similar to how we immunize specific polygenic score comparisons in this paper.

A related point is that for both the coalescent approaches as well as methods relying on direct comparison of polygenic scores, both the evolutionary hypothesis being tested and the degree of susceptibility to bias follow directly from the set of genotype contrasts used in the test. Some authors have suggested that certain coalescent methods of testing for polygenic selection are more robust to stratification bias than others [24, 64, but our results show that this cannot be true: two different methods that test the same evolutionary hypothesis using the same set of estimated effect sizes necessarily have the same susceptibility to stratification bias. Therefore, any observed differences in robustness to stratification bias among methods must come either from changing the evolutionary hypothesis being tested or from differences in ability to accurately estimate the relevant genotype contrasts (i.e. overall differences in statistical power).

We frame our model in terms of detecting an association between polygenic scores and an ancestry gradient of interest due selection acting on genotypes in the test panel. We focus on this scenario because there is a well-defined null hypothesis and residual stratification is known problem. However, none of our results about the source of bias rely on the signal being generated by selection. Other non-neutral process, such as assortative mating along ancestry gradients, phenotype dependent migration, or phenotype dependent ascertainment could generate signals similar to those generated by natural selection. In other settings, researchers may simply be interested in accurately estimating the association between a polygenic score and a given ancestry gradient, whether or not such an association could plausibly have been generated by neutral evolutionary processes or not. Our results apply in this scenario as well.

One disadvantage of our approach is that it requires that the test panel genotypes are available and the test axis specified before carrying out the GWAS. Thus, the benefits cannot immediately be extended to situations where the individual level data for both panels is not available. Tests for association between polygenic scores and axes of ancestry variation are closely related to bivariate LD score regression as applied to a combination of effect estimates for one trait and frequency/genotype contrasts from an independent dataset [65, 19, 32, an approach which does not require the individual level genotypes, but does require access to ancestry-matched LD scores. Previous work in the context of polygenic selection tests raised concerns about spurious inflation of the slope in this context, possibly due to background selection [32]. A more complete analysis of bivariate LD score regression applied in this context would be valuable.

We also note several additional limitations of our approach. First, our model assumes that all loci share the same underlying genetic relatedness matrix. For simple demographic histories like that of our toy model, this is unlikely to be an issue, but could be an issue for more complex demographic histories experienced by real human populations, as mutations of different ages experience the demographic history differently. One solution to this problem would be to bin variants by allele frequency or estimated age [66] and compute different corrections for different frequency/age bins similar to the suggestion by [57] to use principal components computed on rare variants to correct for stratification along axes of recent genetic divergence.

Another limitation is that in real human datasets with complex patterns of genetic variation and confounding, our approach alone will not be sufficient to mitigate all of the negative consequences of confounding. For example, while a GWAS conducted using only our covariate to control for stratification biases would result in effect size estimates that are uncorrelated with the target axis of population structure under the null, stratification along other axes would still function as additional noise in the effect size estimates. This would result in the ascertainment of tag SNPs that are less tightly coupled to their causal variants, and less accurate polygenic scores, reducing power to detect real signals. Therefore, control of population structure via inclusion of top PCs and/or linear mixed models, in addition to the inclusion of our projected test vector, is still desirable.

Finally, we note that the interpretation of results derived from applying our method will be limited by standard assumptions made in many polygenic score association tests and caution interpretation in light of these assumptions [67. Notably, we rely on common tag SNPs, focus on the additive genetic component of a trait, and ignore the potential impact of interactions, either among genetic variants, or between genetic variants and the environment. Further, GWAS can also be impacted by other forms of genetic confounding beyond the simple associations between ancestry and genetic background that we consider here, including that arising from indirect effects, assortative mating, and stabilizing selection [68]. Finally, the interpretation of associations between polygenic scores and axes of ancestry variation remains challenging in general, as differences in polygenic scores do not necessarily translate into differences in phenotype [13], and associations between polygenic scores and patterns of ancestry variation may have counterintuitive evolutionary explanations [69, 39, 38. Therefore, while our results provide a means to protect against stratification bias in polygenic score association tests, addressing a known problem in their implementation, continued care in the interpretation of these analyses is warranted.

## 5. Materials and Methods

### 5.1. Simulating genotypes

We used *msprime* [70] to simulate genotypes under 2 different models with 100 replicates per model. The first model, shown in Figure 1, has two population splits, 200 and 100 generations in past, for a total of 4 present day populations. We fix the population size for all present and past populations to 10,000 diploid individuals. Each replicate consists of 200 independent 1Mb chromosomes with per base mutation and recombination rates equal to 1 × 10^−8^. We then sample 20,000 individuals per population and create two configurations of GWAS and test panels based on the diagrams in Figure 1A and Figure 1C. We down sample the test panels to 2,000 individuals with an equal number from each of the two populations. Finally, we filter out variants with MAF<0.05 in either panel from both panels. When the populations in the GWAS and test panel are non-sister (i.e Figure 1A) the average within panel $F_{ST}\;[?]$ was 0.01, whereas in the configuration in Figure 1C the average $F_{ST}$ was 0.005 .

The second model, modified from [57, is a 6 × 6 stepping stone model where structure extends infinitely far back with a symmetric migration rate of *m* = 0.01. We fix the effective population size to 1,000 diploid individuals and simulate 200 chromosomes per individual. To reduce computational burden, we scale down to 10kb chromosomes with a per base mutation and recombination rates equal to 1 × 10^−7^. We then sample 20 individuals per deme to create a test panel (*N* = 720) and 60 individuals per deme to create the GWAS panel (M = 2160). Again we filter out SNPs with MAF < 0.05 in either panel.

#### 5.1.1. Simulating phenotypes

To study the effect of environmental stratification on association tests, we first simulated nongenetic phenotypes for an individual i in the GWAS panel as $y_{i}\sim ;N(0,\;1)$. In our discrete 4 population model (Figure 1) we then generate a phenotypic difference between populations by adding $\Delta_{AB}$ to $y_{i}$ for individuals in population B. In our grid simulations we generated three different phenotypic gradients where the largest phenotypic shift was always equal to Δ. To generate a latitudinal gradient (Figure 3A) we added $\frac{\Delta }{5}$ to $y_{i}$ for individuals in row 1, $2\frac{\Delta }{5}$ for individuals in row 2, etc. For Figure 3B we generated a gradient along the diagonal by adding $\frac{\Delta }{5}$ to the phenotype for individuals in deme (1,1), $2\frac{\Delta }{5}$ for individuals in deme (2,2), etc. For Figure 3C we shifted the phenotype of individuals in deme (1,4) by Δ. For all grid simulations we varied Δ from 0 to 2 standard deviations (Figure S4) .

To study the effect of controlling for stratification in cases where there is a true signal of association between polygenic scores and the test vector (Figure 2), we used our 4 population demographic model and followed the protocol outlined in [57] to simulate a neutral trait with $h^{2}=0.3$. We first randomly select 1 causal variant per chromosome and sample it’s effect sizes from $\beta_{\ell }\sim ;N\left(0,\sigma_{i}^{2}{\left[p_{\ell }\left(1-p_{\ell }\right)\right]}^{\alpha }\right)$, where $\sigma_{i}^{2}$ is a frequency independent scale of the variance in effect sizes, $p_{\ell }$ is allele frequency in the GWAS panel, and α is a scaling factor controlling the relationship between allele frequency and effect size. We set α=-0.4 and $\sigma_{g}^{2}=\sigma_{i}^{2}\sum_{\ell =1}^{200}\;{\left[2p_{\ell }\left(1-p_{\ell }\right)\right]}^{\alpha +1}=0.3$.

To simulate a signal of true difference in polygenic score in the test panel, we calculate the frequency difference $p_{D,\ell }-p_{C,\ell }$ at all 200 causal sites in the test panel and flip the sign of the effect sizes in the GWAS panel such that $p_{D}-p_{C}>0$ and $\beta_{\ell }>0$ with probability θ. θ therefore controls the strength of the association with θ=0.5 representing no expected association and θ=1 representing the most extreme case where trait increasing alleles are always at a higher frequency in population D. We use θ ranging from 0.5 – 0.62. To calculate ${\overset{‾}{\Delta }}_{CD}$, the true difference in polygenic score between test panel populations, we take 100 simulations with the same θ and average the difference $Z_{D}-Z_{C}$. $Z_{D}$ and $Z_{C}$ are the population average polygenic scores computed using the true causal effects in D and C, respectively. We then draw the environmental component of the phenotype $e_{i,k}\sim ;N\left(0,\;1-h^{2}\right)$ and generate an environmental confounder by adding $\Delta_{AB}$ to $e_{i,k}$ for individuals in population B.

#### 5.1.2. Computing ${\overset{ˆ}{F}}_{Gr}$

We computed a single covariate ${\overset{ˆ}{F}}_{Gr}$ for each polygenic association test. We first construct T as either population ID, latitude or the single deme of interest, depending on the simulation. Given this test vector, we compute $r=X^{⊤}T$ using the plink2 [71] function --glm. Finally we compute ${\overset{ˆ}{F}}_{Gr}$ (see eq. 28 using --sscore in plink2, taking care to standardize by the variance in the GWAS panel genotypes.

#### 5.1.3. GWAS and Polygenic Scores

For each set of phenotypes, we first carried out four separate marginal association GWASs using the regression equations below,

1. $y=\beta_{\ell }G_{\ell }+\epsilon$
2. $y=\beta_{\ell }G_{\ell }+\omega {\overset{ˆ}{F}}_{Gr}+\epsilon$
3. $y=\beta_{\ell }G_{\ell }+\omega ID+\epsilon$
4. $y=\beta_{\ell }G_{\ell }+\omega_{1}{PC}_{1}+\ldots +\omega_{k}{PC}_{k}+\epsilon$

where ID is an indicator of population membership for the discrete 4 population model. This set-up allowed us to compare our covariate ${\overset{ˆ}{F}}_{Gr}$ to a GWAS done with no correction and one done with perfect correction for the discrete model. Additionally, we compared our results to a model that includes some number of top genetic principal components in the GWAS panel. All GWASs were done using the plink2 [71] function --glm.

We then ascertain 200 SNPs to do our association test by picking the SNP with the lowest p-value per chromosome. Additionally, for heritable trait simulations, we know the identity of the underlying causal sites and can choose to use those sites (Figure 2). For each set of sites, either those chosen by p-value or causal sites, we use R to re-estimate effect sizes jointly using the lm() under the four models above, including all 200 SNPs along with the covariates.

Finally, we construct polygenic scores for the individuals in the test panel as ${\overset{ˆ}{Z}}_{i}=\sum_{\ell =1}^{200}\;{\overset{ˆ}{\beta }}_{\ell }X_{\ell }$ where ${\overset{ˆ}{\beta }}_{\ell }$ is the estimated effect size from the joint model and $X_{\ell }$ is the mean centered genotype value for the $\ell^{\text{th}}$ variant.

#### 5.1.4. Polygenic Score Association Test

For each replicate we first compute the test statistic $\overset{ˆ}{q}=\frac{1}{N}{\overset{ˆ}{Z}}^{⊤}T$ by multiplying the vector of polygenic scores for individuals in the test panel by the test vector.

To evaluate the significance of our association statistic, we compute p-values using an empirical null approach. Specifically, we obtain psuedosamples of $\overset{ˆ}{q}$ by randomizing the sign of the effect sizes and recomputing $\overset{ˆ}{q}$. We then obtain a p-value from this sign-flipping null as,

(37) $$P=2\;*\;\frac{\sum_{i}^{N}\;\;I\left(\left|{\overset{ˆ}{q}}_{i}\right|\;>\;\left|{\overset{ˆ}{q}}_{obs}\right|\right)}{K},$$

where K is the number of psuedosamples, here set to 1,000, and I() is an indicator function that equals 1 if the psuedosample $\left|{\overset{ˆ}{q}}_{i}\right|$ is greater than the observed $\left|{\overset{ˆ}{q}}_{obs}\right|$. Finally within a set of 100 evolutionary replicates for each model and confounder combination we compute the positive rate as the proportion of p-values < 0.05.

## Supplementary Material

Supplement 1

Supplement 2
