# Supplementary material for: Testing for differences in polygenic scores in the presence of confounding
Source: bioRxiv. 2024 Jun 26:2023.03.12.532301. Preprint. [Version 4] doi: 10.1101/2023.03.12.532301 (PMC10055004; doi:10.1101/2023.03.12.532301)
Supplement: Supplement 2 [file NIHPP2023.03.12.532301v4-supplement-2.pdf]

bioRxiv preprint doi: https://doi.org/10.1101/2023.03.12.532301; this version posted June 26, 2024. The copyright holder for this preprint (which was not certified by peer review) is the author/funder, who has granted bioRxiv a license to display the preprint in perpetuity. It is made available under aCC-BY 4.0 International license.

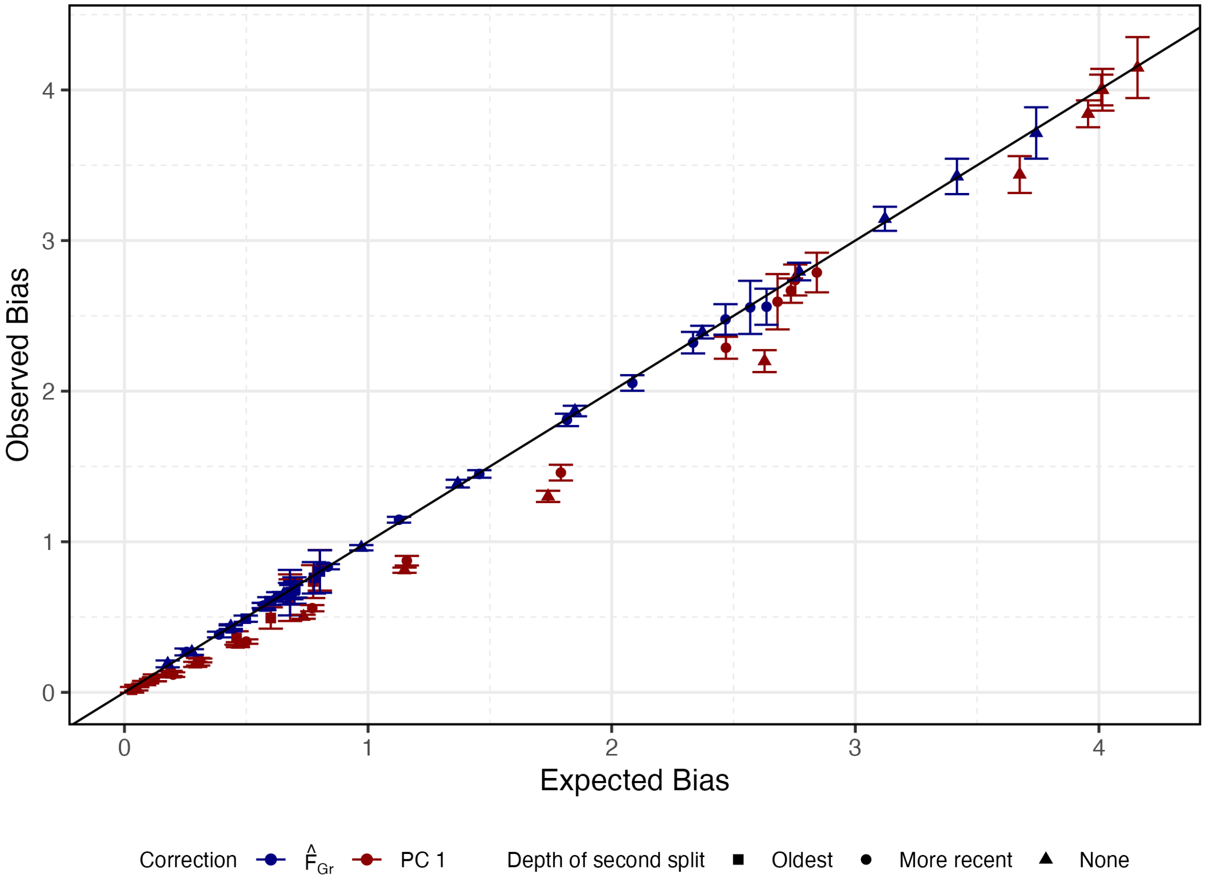

Figure S1: **Error in estimates of $\tilde{F}_{Gr}$ predicts bias in $\hat{q}$ across population models.** For all simulations in Figure 2 we compute the expected bias as $\mathbb{E}\,[\text{Error}] \times \mathbb{E}\,[\hat{q}_{nc}]$ where $\hat{q}_{nc}$ is the observed bias using effect sizes that were estimated with no correction. We then compare this expected bias to the observed bias when using that estimator as a covariate in the GWAS. The error in both $\hat{F}_{Gr}$ and sample PC 1 is highly predictive of the observed bias, though we observe that sample PC 1 exhibits a slight increase in bias reduction compared to the expected.

bioRxiv preprint doi: https://doi.org/10.1101/2023.03.12.532301; this version posted June 26, 2024. The copyright holder for this preprint (which was not certified by peer review) is the author/funder, who has granted bioRxiv a license to display the preprint in perpetuity. It is made available under aCC-BY 4.0 International license.

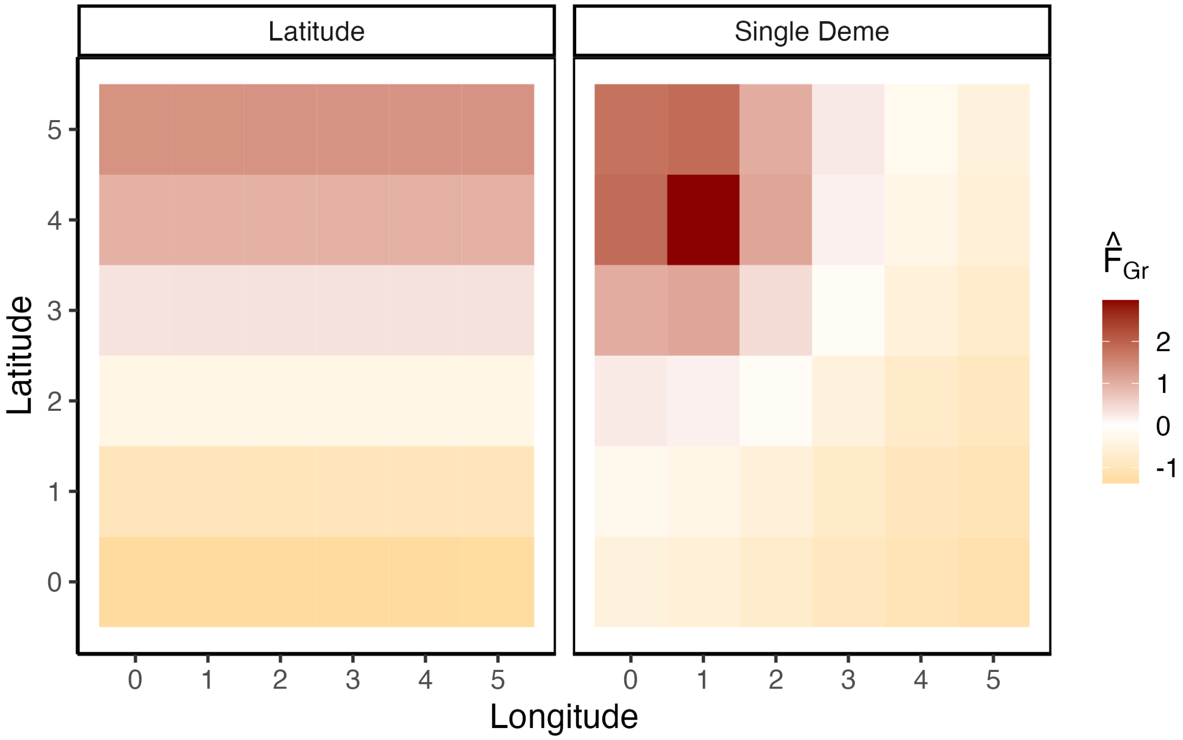

Figure S2: $\hat{F}_{Gr}$ **as observed in the GWAS panel.** For both of the test vectors used in the grid simulations we plotted the average $\hat{F}_{Gr}$ per deme across 100 replicates. For the latitudinal test vector, $\hat{F}_{Gr}$ simply recapitulates latitude, which is unsurprising given the symmetric migration model we use. For the single deme test vector, $\hat{F}_{Gr}$ largely reflects the distance to the focal test deme.

bioRxiv preprint doi: https://doi.org/10.1101/2023.03.12.532301; this version posted June 26, 2024. The copyright holder for this preprint (which was not certified by peer review) is the author/funder, who has granted bioRxiv a license to display the preprint in perpetuity. It is made available under aCC-BY 4.0 International license.

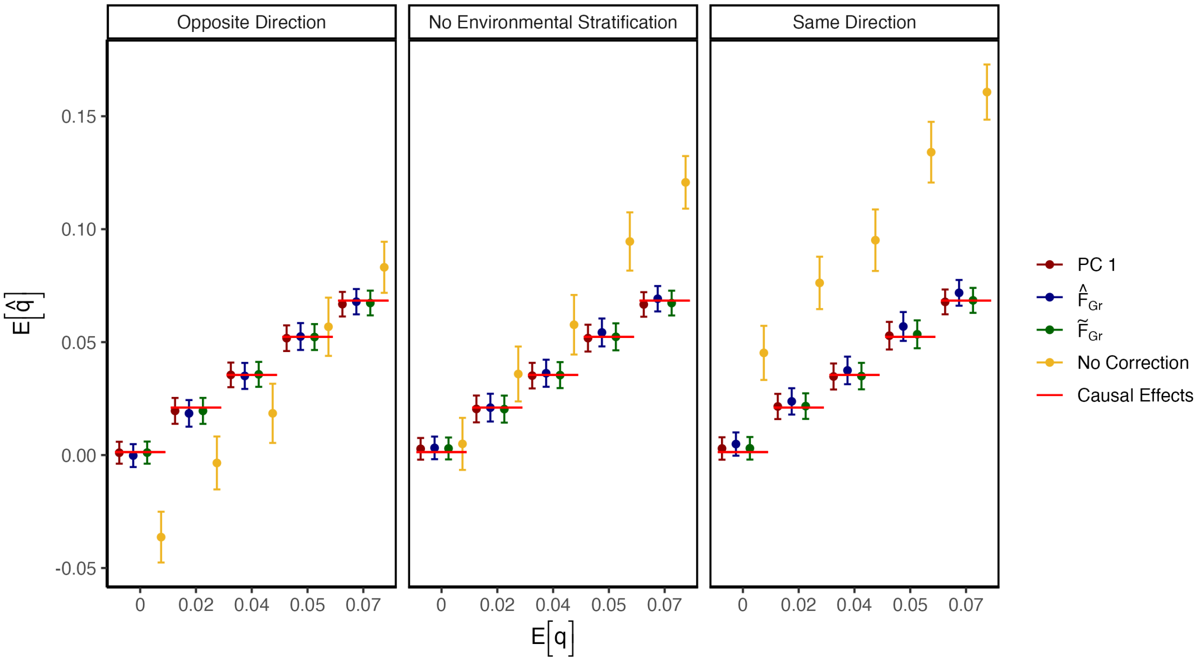

Figure S3: **Including $\tilde{F}_{Gr}$, $\hat{F}_{Gr}$, or PC 1 as a covariate in the GWAS model maintains power to detect true association signal.** GWAS and test panels were simulated in the overlapping structure configuration (see Figure 1A). Heritable phenotypes ($h^2 = 0.3$) were simulated with a true difference in polygenic scores by flipping the sign of a proportion of causal effects to align with allele frequency contrasts, $p_{D,\ell} - p_{C,\ell}$, in the test panel. When stratification is in the same direction as the true difference, $\hat{q}$ is upwardly biased, as it is when there is no environmental stratification, once genetic stratification is strong enough. When stratification is in the opposite direction, environmental and genetic stratification are opposed and the direction of bias depends on the strength of each. As expected, $\tilde{F}_{Gr}$ perfectly captures true association regardless of the direction of stratification. Estimators of $\tilde{F}_{Gr}$ (i.e $\hat{F}_{Gr}$ and PC 1) also capture true association, consistent with out theoretical arguments that downward bias is minimal when $S << L$.
