# Supplementary material for: Testing for differences in polygenic scores in the presence of confounding
Source: bioRxiv. 2023 Aug 22:2023.03.12.532301. Preprint. [Version 3] doi: 10.1101/2023.03.12.532301 (PMC10055004; doi:10.1101/2023.03.12.532301)
Supplement: Supplementary file 1 [file NIHPP2023.03.12.532301v3-supplement-2.pdf]

bioRxiv preprint doi: https://doi.org/10.1101/2023.03.12.532301; this version posted August 22, 2023. The copyright holder for this preprint (which was not certified by peer review) is the author/funder, who has granted bioRxiv a license to display the preprint in perpetuity. It is made available under aCC-BY 4.0 International license.

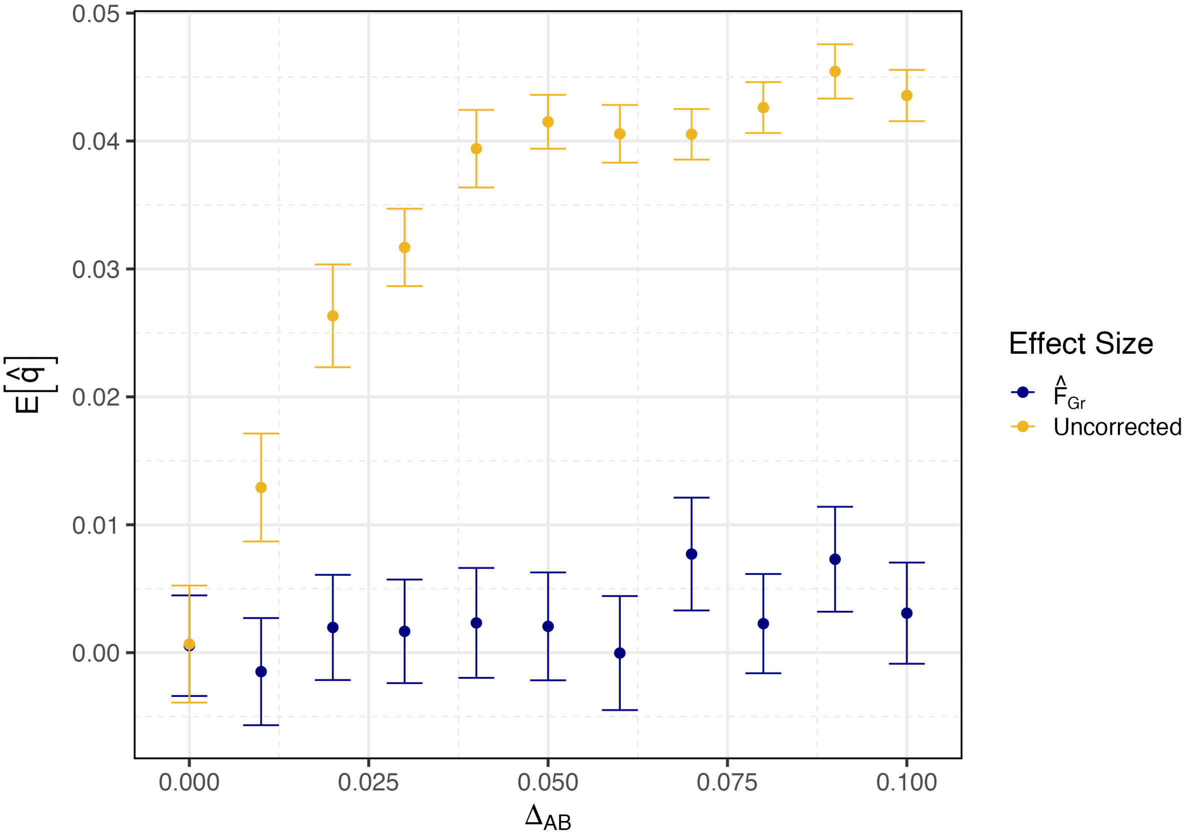

Figure S1: **Including $\hat{F}_{Gr}$ as a covariate removes bias in $\hat{q}$.** In our simulations of the 4 population toy model with overlapping topology (Figure 1A), increasing the magnitude of the confounder increases the average value of $\hat{q}$ when stratification is not controlled for. Including $\hat{F}_{Gr}$ as a covariate in the GWAS model reduces bias in the test statistic.

bioRxiv preprint doi: https://doi.org/10.1101/2023.03.12.532301; this version posted August 22, 2023. The copyright holder for this preprint (which was not certified by peer review) is the author/funder, who has granted bioRxiv a license to display the preprint in perpetuity. It is made available under aCC-BY 4.0 International license.

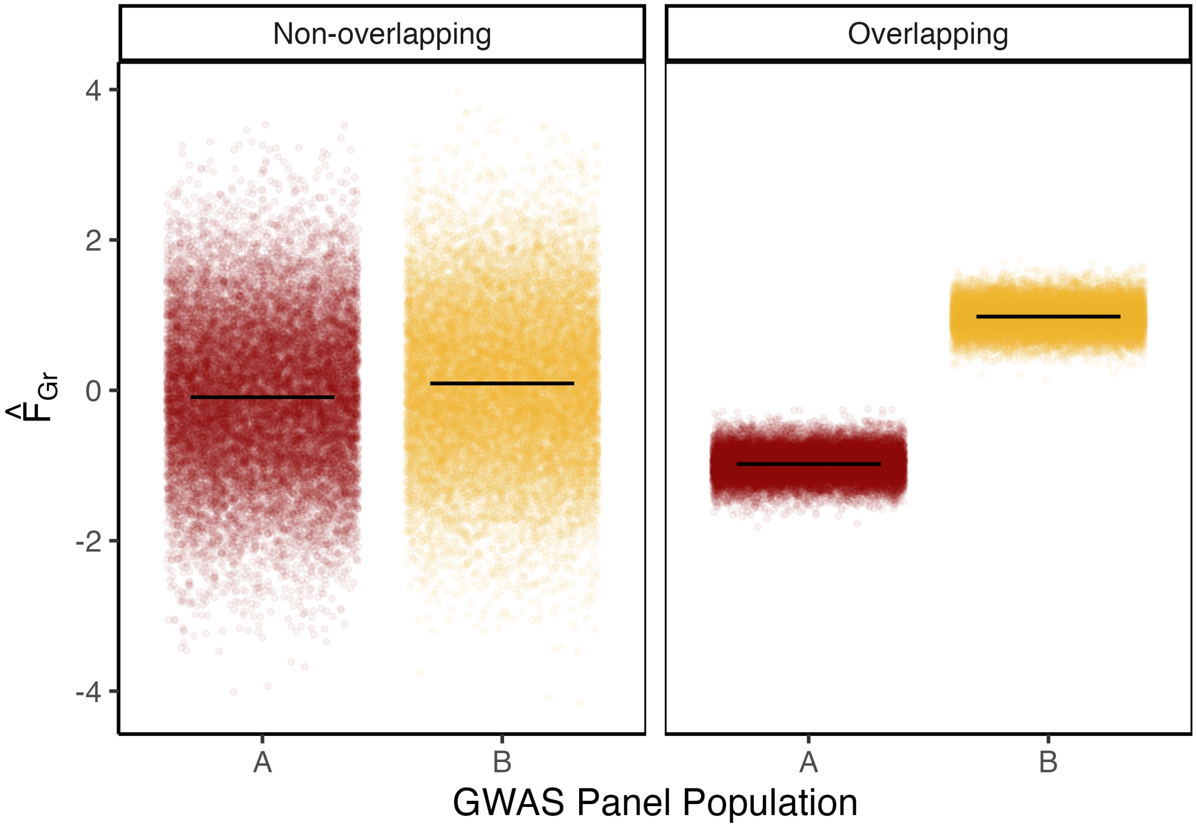

Figure S2: $\hat{F}_{Gr}$ **separates populations in the GWAS panel when test and GWAS panels have overlapping structure.** In the 4 population toy model, when the topology is non-overlapping (Figure 1C), $\hat{F}_{Gr}$, shown here for a single replicate, is purely noise and does not reflect population membership, due to the fact the genotype contrasts in the test panel are uncorrelated with structure in the GWAS panel. When the topology is overlapping (Figure 1A), $\hat{F}_{Gr}$ separates individuals in the GWAS panel based on population membership.

bioRxiv preprint doi: https://doi.org/10.1101/2023.03.12.532301; this version posted August 22, 2023. The copyright holder for this preprint (which was not certified by peer review) is the author/funder, who has granted bioRxiv a license to display the preprint in perpetuity. It is made available under aCC-BY 4.0 International license.

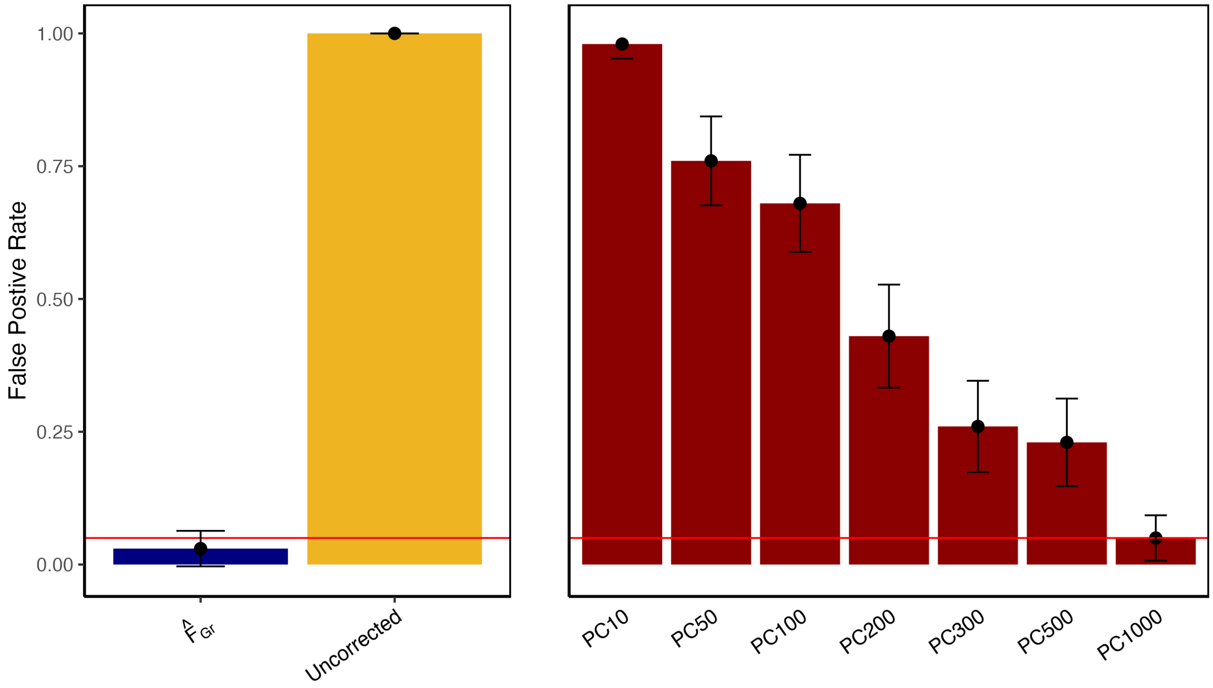

Figure S3: $\hat{F}_{Gr}$ **controls for stratification for a single deme association test.** Here we simulate a 2 standard deviation confounder for a single deme in the GWAS panel (Figure 3C) and conduct an association test for the difference in polygenic score between the same single deme and the rest of the grid in the test panel. Including $\hat{F}_{Gr}$ as a covariate in the GWAS model controls for stratification and the association test using those effect sizes is unbiased (left panel). In the right panel we vary the number of principal components of the GWAS panel included as covariates. It takes 1,000 PCs to return the false positive rate to nominal levels.

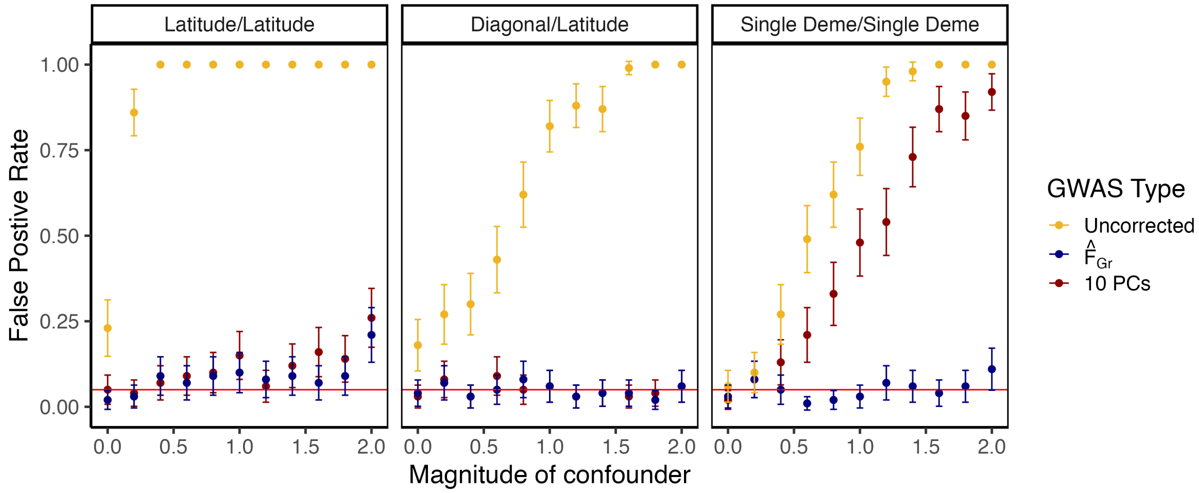

Figure S4: $\hat{F}_{Gr}$ **controls for stratification for a range confounding effects.** For the three simulation scenarios in Figure 3 we plot the false positive rate for the given association test for a range of confounding effects where the magnitude of the confounder is the difference between the most extreme phenotypic shifts in units of standard deviations (see Methods). For all but the strongest magnitude of confounding in the Latitude/Latitude situation, our approach protects the test from stratification bias. In two confounder/test scenarios (left and right columns) the standard approach of including the top 10 GWAS panel principal components begins to fail with large enough confounding.

bioRxiv preprint doi: https://doi.org/10.1101/2023.03.12.532301; this version posted August 22, 2023. The copyright holder for this preprint (which was not certified by peer review) is the author/funder, who has granted bioRxiv a license to display the preprint in perpetuity. It is made available under aCC-BY 4.0 International license.

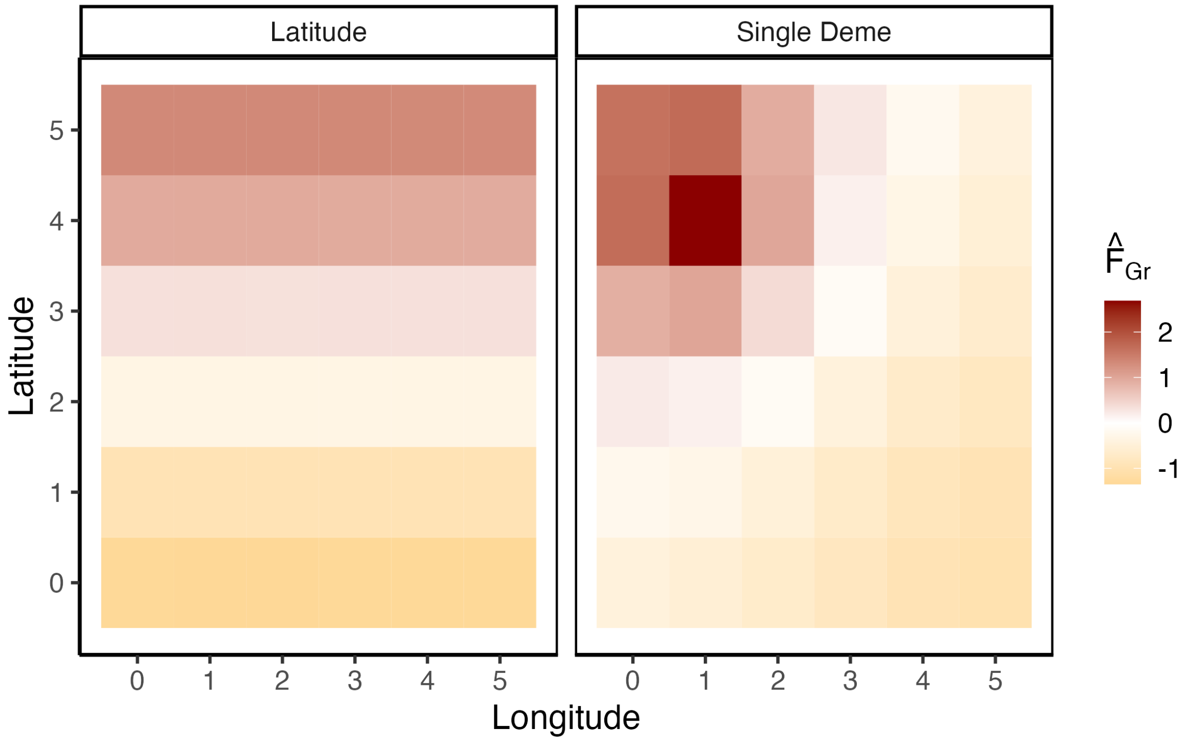

Figure S5: $\hat{F}_{Gr}$ **predicts T if measured in the GWAS panel.** For both of the test vectors in Figure 3 we plotted the average $\hat{F}_{Gr}$ per deme across 100 replicates. Because the test and GWAS panel are sampled from the same demographic model and have completely overlapping structure, $\hat{F}_{Gr}$ reflects the spatial pattern of T.

bioRxiv preprint doi: https://doi.org/10.1101/2023.03.12.532301; this version posted August 22, 2023. The copyright holder for this preprint (which was not certified by peer review) is the author/funder, who has granted bioRxiv a license to display the preprint in perpetuity. It is made available under aCC-BY 4.0 International license.

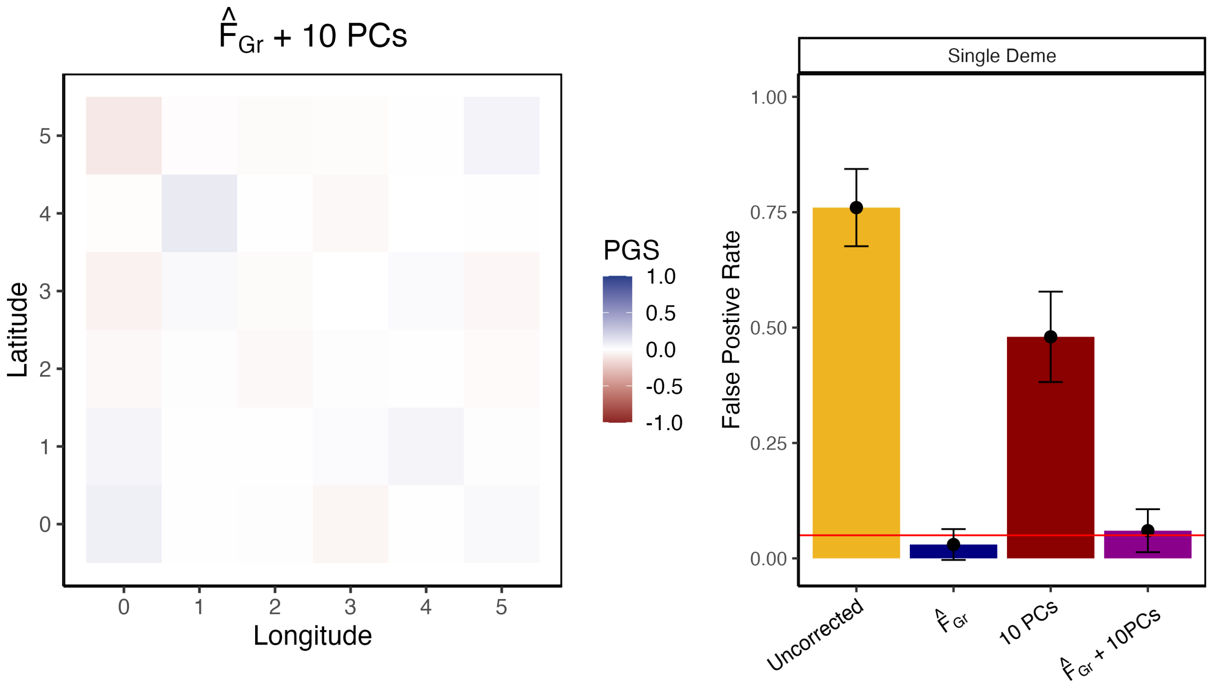

Figure S6: **Including $\hat{F}_{Gr}$ and 10 PCs protects the test and removes spatial patterns in the PGSs.** For the single deme confounder and test in Figure 3C we included both $\hat{F}_{Gr}$ and the top 10 PCs as covariates in the GWAS model. When only $\hat{F}_{Gr}$ is included the test is protected, but a residual pattern in the polygenic scores induced (Figure 3C, third column). Including both $\hat{F}_{Gr}$ and the top 10 PCs continues to protect the test (right) and removes the residual pattern (left).
