# Supplementary material for: Testing for differences in polygenic scores in the presence of confounding
Source: bioRxiv. 2023 Aug 22:2023.03.12.532301. Preprint. [Version 3] doi: 10.1101/2023.03.12.532301 (PMC10055004; doi:10.1101/2023.03.12.532301)
Supplement: Supplementary file 2 [file media-1.pdf]

Supplementary Information for "Testing for differences in polygenic scores in the presence of confounding"

Jennifer Blanc[1*], Jeremy J. Berg[1]

[1]Department of Human Genetics, University of Chicago, Chicago, IL, USA
[*]To whom correspondence should be addressed: jgblanc@uchicago.edu, jjberg@uchicago.edu

August 16, 2023

Contents

S1 Expectation of marginal effects **2**

S2 Expectation of polygenic scores using marginal effects **3**

S3 Polygenic score association test bias using marginal effects **4**

S4 Expectation of polygenic scores while controlling for $\tilde{\mathbf{F}}_{Gr}$ **5**

S5 Polygenic score association test bias controlling for $\tilde{\mathbf{F}}_{Gr}$ **6**

S6 Modeling the correlation structure among test panel individuals **7**

S1 Expectation of marginal effects

Conditional on the GWAS panel genotypes and the genetic and environmental components of the phenotype, the marginal association at site ℓ is given by

$$\hat{\beta}_\ell \mid G_\ell, u, e = \frac{y^\top G_\ell}{G_\ell^\top G_\ell} \tag{S1}$$

$$= \beta_\ell + \frac{u_{-\ell}^\top G_\ell}{G_\ell^\top G_\ell} + \frac{e^\top G_\ell}{G_\ell^\top G_\ell} \tag{S2}$$

(equation (15) in the main text). Thus, taking expectation over the randomness in the GWAS panel genotypes, the total genetic effect, and the environmental effect on the phenotype, the expected effect size estimate at site ℓ is

$$\mathbb{E}\left[\hat{\beta}_\ell\right] = \beta_\ell + \mathbb{E}\left[\frac{u_{-\ell}^\top G_\ell}{G_\ell^\top G_\ell}\right] + \mathbb{E}\left[\frac{e^\top G_\ell}{G_\ell^\top G_\ell}\right] \tag{S3}$$

$$= \beta_\ell + \mathbb{E}\left[u_{-\ell}^\top\right]\mathbb{E}\left[\frac{G_\ell}{G_\ell^\top G_\ell}\right] + \mathbb{E}\left[e^\top\right]\mathbb{E}\left[\frac{G_\ell}{G_\ell^\top G_\ell}\right] \tag{S4}$$

$$= \beta_\ell + u_{S,-\ell}^\top \mathbb{E}\left[\frac{G_\ell}{G_\ell^\top G_\ell}\right] + c^\top \mathbb{E}\left[\frac{G_\ell}{G_\ell^\top G_\ell}\right] \tag{S5}$$

$$\approx \beta_\ell + u_{S,-\ell}^\top \frac{G_{\ell,S}}{4\lambda_\ell(1-\lambda_\ell)(1+\overline{F}_G)} + c^\top \frac{G_{\ell,S}}{4\lambda_\ell(1-\lambda_\ell)(1+\overline{F}_G)} \tag{S6}$$

$$\approx \beta_\ell + \alpha\beta_\ell A_G^\top V_{A,-\ell}^{PGS} \frac{\alpha A_G}{2(1+\overline{F}_G)} + c^\top \frac{\alpha A_G}{2(1+\overline{F}_G)} \tag{S7}$$

$$\approx \beta_\ell + \frac{\alpha^2 \beta_\ell V_{A,-\ell}^{PGS}}{2(1+\overline{F}_G)} + \frac{\alpha c^\top A_G}{2(1+\overline{F}_G)} \tag{S8}$$

$$\approx \beta_\ell \left(1 + \frac{1}{2}\frac{\alpha^2 V_{A,-\ell}^{PGS}}{1+\overline{F}_G}\right) + \frac{1}{2}\frac{\alpha c^\top A_G}{1+\overline{F}_G} \tag{S9}$$

where $1 + \overline{F_G} = 1 + \frac{1}{M}\sum_{m=1}^{M} f_{mm}$ is the average level of self relatedness in the GWAS panel. The approximation at line (S6) comes from approximating the expectation of the ratio as the ratio of expectations. Therefore, consistent with results from [1], the expected marginal effect estimate for a given site is expected to be equal to the causal effect if the null holds (i.e. if $\alpha = 0$). In contrast, under our alternative model, the focal site and the genetic background have a positive covariance as they are both influenced by selection. As a result, the expected marginal effect is biased away from zero (i.e. further in the direction of the causal effect), and may be biased in either direction depending on whether the environmental confounder is positively or negatively associated with the axis along which selection has perturbed the focal allele. For the following analyses concerning bias in the distribution of polygenic scores and in polygenic score association tests (see section S2) we assume that the above biases in marginal effect estimates induced by selection are small relative to those induced by shared drift between the GWAS and test panels [2, 3, 4, 5, 6].

S2 Expectation of polygenic scores using marginal effects

Given the marginal effect estimates, the vector of N polygenic scores in the test panel is given by,

$$\hat{Z}^\top \mid \mathbf{G}, \mathbf{X}, u, e = \sum_{\ell=1}^{S} \hat{\beta}_\ell X_\ell^\top \tag{S10}$$

$$= \sum_{\ell=1}^{S} \beta_\ell X_\ell + \sum_{\ell=1}^{S} \frac{u_{-\ell}^\top G_\ell}{G_\ell^\top G_\ell} X_\ell^\top + \frac{e^\top G_\ell}{G_\ell^\top G_\ell} X_\ell^\top \tag{S11}$$

so the expected polygenic scores can be written as

$$\mathbb{E}\left[\hat{Z}^\top\right] = \sum_{\ell=1}^{S} \mathbb{E}\left[\beta_\ell X_\ell\right] + \sum_{\ell=1}^{S} \mathbb{E}\left[\frac{u_{-\ell}^\top G_\ell}{G_\ell^\top G_\ell} X_\ell^\top\right] + \sum_{\ell=1}^{S} \mathbb{E}\left[\frac{e^\top G_\ell}{G_\ell^\top G_\ell} X_\ell^\top\right]. \tag{S12}$$

The first sum is the expected "true" polygenic scores given the non-neutral effects on the test panel genotypes ($\sum_{\ell=1}^{S} \mathbb{E}\left[\beta_\ell X_\ell\right] = \sum_{\ell=1}^{S} \beta_\ell X_{\ell,S} = \mathbb{E}[Z]^\top = \alpha V_A A_X$) while the second sum captures contributions to the polygenic scores due to associations between the genotypes at individual focal sites and the genetic component of the phenotype from all other sites ($u_{-\ell} = \sum_{\ell' \neq \ell} \beta_{\ell'} G_{\ell'}$). The third captures contributions due to associations between the genotypes and the environmental component. We consider each of the second two in turn, beginning with the environment, which is more straightforward.

For each site ℓ, we can write

$$\mathbb{E}\left[\frac{e^\top G_\ell}{G_\ell^\top G_\ell} X_\ell^\top\right] = \mathbb{E}\left[e^\top\right] \mathbb{E}\left[\frac{G_\ell X_\ell^\top}{G_\ell^\top G_\ell}\right] \tag{S13}$$

$$= \frac{c^\top}{M} \mathbb{E}\left[\frac{G_\ell X_\ell^\top}{G_\ell^\top G_\ell / M}\right] \tag{S14}$$

$$\approx \frac{c^\top}{M} \mathbb{E}\left[\frac{G_{\ell,D} X_{\ell,D}^\top}{(G_{\ell,D}+G_{\ell,B})^\top (G_{\ell,D}+G_{\ell,B}) / M}\right] = \frac{1}{M} c^\top \tilde{\mathbf{F}}_{GX} \tag{S15}$$

$$\approx \frac{c^\top}{M} \frac{\mathbb{E}\left[G_{\ell,D} X_{\ell,D}^\top\right]}{\mathbb{E}\left[(G_{\ell,D}+G_{\ell,B})^\top (G_{\ell,D}+G_{\ell,B}) / M\right]} = \frac{1}{M} \frac{c^\top \mathbf{F}_{GX}}{\left(1 + \overline{F_G}\right)}. \tag{S16}$$

The approximation in line (S15) arises from assuming that $G_{\ell,S}$ is small compared to $G_{\ell,D}$, and can therefore be ignored. Alternately, (S15) is exact under the null that $\alpha = 0$. In line (S16), we make a ratio of expectations approximation. $\tilde{\mathbf{F}}_{GX}$ is the expected kinship matrix computed on standardized genotypes, which is approximately equal to $\frac{\mathbf{F}_{GX}}{(1+\overline{F_G})}$, with the approximation being better if $\overline{F_G}$ is small. Summing across sites, we have

$$\sum_{\ell=1}^{S} \mathbb{E}\left[\frac{e^\top G_\ell}{G_\ell^\top G_\ell} X_\ell^\top\right] \approx \frac{S}{M} c^\top \tilde{\mathbf{F}}_{GX}. \tag{S17}$$

where S is the number of causal sites.

For the contributions to the polygenic score arising from associations between genotypes and the genetic component of the phenotype, we have

$$\begin{aligned}
\mathbb{E}\left[\frac{u_{-\ell}^\top G_\ell}{G_\ell^\top G_\ell} X_\ell^\top\right] &= \mathbb{E}\left[u_{-\ell}^\top\right]\mathbb{E}\left[\frac{G_\ell X_\ell^\top}{G_\ell^\top G_\ell}\right] && \text{(S18)}\\
&= \frac{u_{S,-\ell}^\top}{M}\mathbb{E}\left[\frac{G_\ell X_\ell^\top}{G_\ell^\top G_\ell / M}\right] && \text{(S19)}\\
&\approx \frac{u_{S,-\ell}^\top}{M}\mathbb{E}\left[\frac{G_{\ell,D} X_{\ell,D}^\top}{(G_{\ell,D}+G_{\ell,B})^\top (G_{\ell,D}+G_{\ell,B})/M}\right] = \frac{1}{M} u_{S,-\ell}^\top \tilde{\mathbf{F}}_{GX} && \text{(S20)}\\
&\approx \frac{u_{S,-\ell}^\top}{M}\frac{\mathbb{E}\left[G_{\ell,D} X_{\ell,D}^\top\right]}{\mathbb{E}\left[(G_{\ell,D}+G_{\ell,B})^\top (G_{\ell,D}+G_{\ell,B})/M\right]} = \frac{1}{M}\frac{u_{S,-\ell}^\top \mathbf{F}_{GX}}{\left(1+\overline{F_G}\right)}, && \text{(S21)}
\end{aligned}$$

where $u_{S,-\ell} = \sum_{\ell' \neq \ell} \beta_{\ell'} G_{S,\ell'} = u_S - \beta_\ell G_{\ell,S}$. All sites in our model are unlinked, allowing us to treat $u_{-\ell}$ as independent from G_ℓ.

Summing across sites, we have

$$\begin{aligned}
\sum_{\ell=1}^{S}\mathbb{E}\left[\frac{u_{-\ell}^\top G_\ell}{G_\ell^\top G_\ell} X_\ell^\top\right] &\approx \frac{1}{M}\sum_{\ell=1}^{S} u_{S,-\ell}^\top \tilde{\mathbf{F}}_{GX} && \text{(S22)}\\
&\approx \frac{1}{M}\sum_{\ell=1}^{S}\left(u_S^\top - \beta_\ell G_{\ell,S}^\top\right)\tilde{\mathbf{F}}_{GX} && \text{(S23)}\\
&\approx \frac{1}{M}\left(S u_S^\top - u_S^\top\right)\tilde{\mathbf{F}}_{GX} && \text{(S24)}\\
&\approx \frac{S-1}{M} u_S^\top \tilde{\mathbf{F}}_{GX} && \text{(S25)}\\
&\approx \frac{S}{M} u_S^\top \tilde{\mathbf{F}}_{GX}. && \text{(S26)}
\end{aligned}$$

Putting genetic and environmental contributions together, the expected polygenic scores are approximately

$$\begin{aligned}
\mathbb{E}\left[\hat{Z}^\top\right] &= \sum_{\ell=1}^{S}\mathbb{E}\left[\beta_\ell X_\ell\right] + \sum_{\ell=1}^{S}\mathbb{E}\left[\frac{u^\top G_\ell}{G_\ell^\top G_\ell} X_\ell^\top\right] + \sum_{\ell=1}^{S}\mathbb{E}\left[\frac{e^\top G_\ell}{G_\ell^\top G_\ell} X_\ell^\top\right] && \text{(S27)}\\
&\approx \mathbb{E}\left[Z\right]^\top + \frac{S}{M}\left(u_S^\top + c^\top\right)\tilde{\mathbf{F}}_{GX}. && \text{(S28)}
\end{aligned}$$

S3 Polygenic score association test bias using marginal effects

The expected value of our test statistic, $\hat{q}$, follows straightforwardly from the expected values of the polygenic scores. We have

$$\begin{aligned}
\mathbb{E}\left[\hat{q}\right] &= \frac{1}{N}\mathbb{E}\left[\hat{Z}^\top\right] T && \text{(S29)}\\
&\approx \mathbb{E}\left[q\right] + \frac{S}{NM}\left(\mu_S^\top + c^\top\right)\tilde{\mathbf{F}}_{GX} T = \mathbb{E}\left[q\right] + \frac{S}{NM}\left(\mu_S^\top + c^\top\right)\tilde{\mathbf{F}}_{Gr}. && \text{(S30)}
\end{aligned}$$

The bias in $\hat{q}$ is therefore approximately equal to

$$\mathbb{E}\left[\hat{q}-q\right] \approx \mathbb{E}[\hat{q}] - \mathbb{E}\left[q\right] \tag{S31}$$

$$\approx \frac{S}{NM}\left(\mu_S^\top + c^\top\right)\tilde{\mathbf{F}}_{GX}T = \frac{S}{NM}\left(\mu_S^\top + c^\top\right)\tilde{\mathbf{F}}_{Gr}. \tag{S32}$$

S4 Expectation of polygenic scores while controlling for $\tilde{\mathbf{F}}_{Gr}$

Now, controlling for $\tilde{F}_{Gr}$, the marginal effects are

$$\hat{\beta}'_\ell \mid G_\ell, u, e = \beta_\ell + \frac{u_{-\ell}^\top \mathbf{P} G_\ell}{G_\ell^\top \mathbf{P} G_\ell} + \frac{e^\top \mathbf{P} G_\ell}{G_\ell^\top \mathbf{P} G_\ell} \tag{S33}$$

where $\mathbf{P} = \left(\mathbf{I} - \frac{1}{\|\tilde{F}_{Gr}\|}\tilde{F}_{Gr}\tilde{F}_{Gr}^\top\right)$

Thus, conditional on the variables in the GWAS panel, the polygenic scores are

$$Z^\top \mid \mathbf{G}, u, e = \sum_{\ell=1}^{S} \hat{\beta}'_\ell X_\ell^\top \tag{S34}$$

$$= \sum_{\ell=1}^{S} \beta_\ell X_\ell + \sum_{\ell=1}^{S} \frac{u^\top \mathbf{P} G_\ell}{G_\ell^\top \mathbf{P} G_\ell} X_\ell^\top + \sum_{\ell=1}^{S} \frac{e^\top \mathbf{P} G_\ell}{G_\ell^\top \mathbf{P} G_\ell} X_\ell^\top \tag{S35}$$

so the expected polygenic scores can be written as

$$\mathbb{E}\left[Z^\top\right] = \sum_{\ell=1}^{S} \mathbb{E}\left[\beta_\ell X_\ell\right] + \sum_{\ell=1}^{S} \mathbb{E}\left[\frac{u^\top \mathbf{P} G_\ell}{G_\ell^\top \mathbf{P} G_\ell} X_\ell^\top\right] + \sum_{\ell=1}^{S} \mathbb{E}\left[\frac{e^\top \mathbf{P} G_\ell}{G_\ell^\top \mathbf{P} G_\ell} X_\ell^\top\right]. \tag{S36}$$

Following the same steps as above (see S13, S18, and S22), we take the expectation of each of the above terms separately. The first term is again the expected "true" polygenic scores ($\sum_{\ell=1}^{S} \mathbb{E}\left[\beta_\ell X_\ell\right] = \sum_{\ell=1}^{S} \beta_\ell X_{\ell,S} = \mathbb{E}[Z]^\top$). Then we consider the association between the environment and the residual genotypes,

$$\mathbb{E}\left[\frac{e^\top \mathbf{P} G_\ell}{G_\ell^\top \mathbf{P} G_\ell} X_\ell^\top\right] = \mathbb{E}\left[e^\top\right] \mathbf{P} \mathbb{E}\left[\frac{G_\ell X_\ell^\top}{G_\ell^\top \mathbf{P} G_\ell}\right] \tag{S37}$$

$$\approx \frac{c^\top}{M} \mathbf{P} \mathbb{E}\left[\frac{G_{\ell,D} X_{\ell,D}^\top}{(G_{\ell,D}+G_{\ell,B})^\top \mathbf{P} (G_{\ell,D}+G_{\ell,B})/M}\right] \tag{S38}$$

$$\approx \frac{c^\top}{M} \mathbf{P} \tilde{\mathbf{F}}_{GX} \tag{S39}$$

$$\approx \frac{c^\top}{M}\left(\tilde{\mathbf{F}}_{GX} - \frac{1}{\|\tilde{F}_{Gr}\|}\tilde{F}_{Gr}\tilde{F}_{Gr}^\top \tilde{\mathbf{F}}_{GX}\right) \tag{S40}$$

$$\approx \frac{c^\top}{M} \tilde{\mathbf{F}}_{GX}^{\perp \tilde{F}_{Gr}} \tag{S41}$$

where we make an additional approximation by ignoring the $\mathbf{P}$ in the denominator, which is reasonable so long as $\tilde{F}_{Gr}$ explains only a small fraction of the variance in the GWAS panel genotypes. Summing of the S causal sites in the polygenic score,

$$\sum_{\ell=1}^{S} \mathbb{E}\left[\frac{e^\top \mathbf{P} G_\ell}{G_\ell^\top \mathbf{P} G_\ell} X_\ell\right] \approx \frac{S}{M} c^\top \tilde{\mathbf{F}}_{GX}^{\perp \tilde{F}_{Gr}} \tag{S42}$$

Turning to the contribution to the polygenic score arising from the association between focal site and the residual genetic background, the expectation is

$$\mathbb{E}\left[\frac{u_{-\ell}^\top \mathbf{P} G_\ell}{G_\ell^\top \mathbf{P} G_\ell} X_\ell^\top\right] = \mathbb{E}\left[u_{-\ell}^\top\right] \mathbf{P} \mathbb{E}\left[\frac{G_\ell X_\ell^\top}{G_\ell^\top \mathbf{P} G_\ell}\right] \tag{S43}$$

$$\approx \frac{u_{S,-\ell}^\top}{M} \mathbf{P} \mathbb{E}\left[\frac{G_{\ell,D} X_{\ell,D}^\top}{(G_{\ell,D}+G_{\ell,B})^\top \mathbf{P} (G_{\ell,D}+G_{\ell,B})/M}\right] \tag{S44}$$

$$\approx \frac{u_{S,-\ell}^\top}{M} \left(\tilde{\mathbf{F}}_{GX} - \frac{1}{\|\tilde{F}_{Gr}\|} \tilde{F}_{Gr} \tilde{F}_{Gr}^\top \tilde{\mathbf{F}}_{GX}\right) \tag{S45}$$

$$\approx \frac{u_{S,-\ell}^\top}{M} \tilde{\mathbf{F}}_{GX}^{\perp \tilde{F}_{Gr}} \tag{S46}$$

where we again make the approximation of ignoring $\mathbf{P}$ in the denominator. Summing across the S sites,

$$\sum_{\ell=1}^{S} \mathbb{E}\left[\frac{u^\top \mathbf{P} G_\ell}{G_\ell^\top \mathbf{P} G_\ell} X_\ell\right] \approx \frac{1}{M} \sum_{\ell=1}^{S} u_{S,-\ell}^\top \tilde{\mathbf{F}}_{GX}^{\perp \tilde{F}_{Gr}} \tag{S47}$$

$$\approx \frac{S}{M} u_S^\top \tilde{\mathbf{F}}_{GX}^{\perp \tilde{F}_{Gr}} \tag{S48}$$

Putting the true polygenic scores and the residual genetic and environmental contributions together, the expected polygenic scores after controlling for $\tilde{F}_{Gr}$ are approximately,

$$\mathbb{E}\left[\hat{Z}\right] \approx \mathbb{E}[Z] + \frac{S}{M}\left(\mu_S^\top + c^\top\right) \tilde{\mathbf{F}}_{GX}^{\perp \tilde{F}_{Gr}}. \tag{S49}$$

S5 Polygenic score association test bias controlling for $\tilde{\mathbf{F}}_{Gr}$

Following from the distribution of polygenic scores after controlling for $\tilde{\mathbf{F}}_{Gr}$, the expected association test statistic is,

$$\mathbb{E}[\hat{q}] \approx \mathbb{E}[q] + \frac{S}{M}\left(\mu_S^\top + c^\top\right) \tilde{\mathbf{F}}_{GX}^{\perp \tilde{F}_{Gr}} T \tag{S50}$$

$$\approx \mathbb{E}[q] + \frac{S}{M}\left(\mu_S^\top + c^\top\right)\left(\tilde{\mathbf{F}}_{GX} - \frac{1}{\|\tilde{F}_{Gr}\|} \tilde{F}_{Gr} \tilde{F}_{Gr}^\top \tilde{\mathbf{F}}_{GX}\right) T \tag{S51}$$

$$\approx \mathbb{E}[q] + \frac{S}{M}\left(\mu_S^\top + c^\top\right)\left(\tilde{F}_{Gr} - \frac{1}{\|\tilde{F}_{Gr}\|} \tilde{F}_{Gr} \tilde{F}_{Gr}^\top \tilde{F}_{Gr}\right) \tag{S52}$$

$$\approx \mathbb{E}[q] + \frac{S}{M}\left(\mu_S^\top + c^\top\right)\left(\tilde{F}_{Gr} - \tilde{F}_{Gr}\right) \tag{S53}$$

$$\approx \mathbb{E}[q] \tag{S54}$$

such that the expected bias is

$$\mathbb{E}\left[\hat{q}-q\right]\approx 0. \tag{S55}$$

Here we highlight a reasonable assumption we make regarding our ability to detect true signal when controlling for $\tilde{F}_{Gr}$. As discussed in the main text, including $\tilde{F}_{Gr}$ as a covariate removes any correlation between $\hat{\beta}$ and r under the null hypothesis where $\alpha = 0$. Notably, under the alternative hypothesis, our ability to detect signal is preserved as we use variation along other axes in the GWAS panel to estimate effect sizes which in turn can still be correlated with r_S in the test panel. We therefore rely on the assumption that for site ℓ there is enough remaining variation in G_ℓ after regressing out $\tilde{F}_{Gr}$ to estimate $\hat{\beta}_\ell$. If all variation in G_ℓ lies along $\tilde{F}_{Gr}$, $y^\top \mathbf{P} G_\ell \approx 0$ and we will be unable to estimate $\hat{\beta}_\ell$. For example, if $\tilde{F}_{Gr}$ represented individuals on opposite sides of a population split this would only apply to sites with fixed differences. This is unlikely to be a concern in practice as F_{ST} is low in human populations [7] and most variants will not be perfectly correlated with a single axis of structure. Therefore, it is reasonable to assume that it will still be possible to accurately estimate β_ℓ, albeit with slightly larger standard error.

S6 Modeling the correlation structure among test panel individuals

In the main text, we test for the association between polygenic scores and the test vector using the linear model,

$$Z = qT + \varepsilon \tag{S56}$$

where ε is i.i.d Normal across individuals. If T is scaled to have a variance of 1, the slope is given by,

$$q = \frac{1}{N} Z^\top T \tag{S57}$$

which we use as our test statistic of interest.

However, a more powerful test is available by modeling the correlation structure among individuals in the test panel. Under our genotypic model the expected pattern of covariance of genotypes at site among individuals in the test panel is,

$$Var\left(X_\ell\right) = \mathbb{E}[X_\ell X_\ell^\top] = 4\lambda_\ell\left(1-\lambda_\ell\right)\left(\mathbf{F}^*_{XX}\right), \tag{S58}$$

where $\mathbf{F}^*_{XX} = \mathbf{F}_{XX} + \mathbf{B}_X$. $\mathbf{F}_{XX}$ contains the within panel relatedness coefficients and $\mathbf{B}_X$ is a diagonal matrix with entries specifying the amount of additional variance for each individual due to sampling.

Now, because the polygenic scores are sums across a large number of sites, under the null model the distribution of standardized polygenic scores is approximately multivariate Normal

$$\frac{Z}{\sqrt{2V_A}} \sim MVN\left(0, \mathbf{F}^*_{XX}\right) \tag{S59}$$

where $V_A = 2\sum_{\ell}^{S} \beta_\ell^2 \lambda_\ell (1-\lambda_\ell)$ is the additive genetic variance of the polygenic scores in the original ancestral population.

To test for evidence of an association, we compare this null model to an alternative in which polygenic scores are associated with T,

$$\frac{Z}{\sqrt{2V_A}} \sim MVN\left(T\gamma, \mathbf{F}^*_{XX}\right), \tag{S60}$$

where γ is the slope. Here we assume that T is mean centered and scaled such that $\mathbf{F}^{*-\frac{1}{2}}_{XX} T$ has a variance of one. With this scaling, the generalized least squares estimate of γ is given by $\hat{\gamma} = {T^\top \mathbf{F}^{*-1}_{XX} Z}/{(N\sqrt{2V_A})}$, where the $\mathbf{F}^{*-1}_{XX}$ term accounts for the evolutionary non-independence of the individuals in the panel. Under the null hypothesis, $\hat{\gamma} \sim N\left(0, \frac{1}{N}\right)$.

To remove this dependence on the sample size, we let the test statistic be $q_x = \sqrt{N}\hat{\gamma}$, so that

$$q_x = \frac{Z^\top \mathbf{F}^{*-1}_{XX} T}{\sqrt{2NV_A}} \tag{S61}$$

$$= \frac{1}{\sqrt{2NV_A}} \sum_{\ell=1}^{S} \beta_\ell X_\ell^\top \mathbf{F}^{*-1}_{XX} T \tag{S62}$$

$$= \frac{1}{\sqrt{2NV_A}} \beta r_x. \tag{S63}$$

Under the null $q_x \sim N(0,1)$. This test statistic is related to Berg and Coop's Q_X statistic [6, 8] in a straight-forward way. For a test of selection along a single axis of ancestry variation, $Q_X = q_x^2$. More generally, the Q_X statistic combines multiple tests along different axes of ancestry variation, accounting for non-independence among axes and providing the appropriate multiple testing penalty.

As discussed above, q_x is proportional to the generalized least squares estimate of γ whereas q in the main text is proportional to the ordinary least squares estimate. Therefore, q_x accounts for covariance and heteroscedasticity in test panel genotypes caused by within test panel population structure. This results in a more powerful test, in precisely the same way that linear mixed models increase the power of association tests in GWAS by accounting for covariance in the phenotypes due to genetic relatedness.

To apply our correction procedure while accounting for the non-independence among test panel individuals, we can compute $r_{x,\ell} = X_\ell^\top \hat{\mathbf{F}}^{*-1}_{XX} T$ and plug r_x into equation 28 in the main text instead of r_ℓ. This version of $\hat{F}_{Gr}$ can again be included as a covariate in the GWAS and those corrected effect sizes, in conjunction with r_x can then be used to compute q_x. Overall, we would expect this procedure to result in slightly stronger protection against stratification bias, as the $r_{x,\ell}$ will have a slightly lower variance than the r_ℓ, and thus lead to a more accurate estimated $\hat{F}_{Gr}$, though we have not systematically investigated this.

References

1. Robinson MR, Hemani G, Medina-Gomez C, Mezzavilla M, Esko T, Shakhbazov K, et al. Population genetic differentiation of height and body mass index across Europe. Nature Genetics. 2015;47(11):1357–1362. https://doi.org/10.1038/ng.3401.

2. Bulmer MG. The Effect of Selection on Genetic Variability. The American Naturalist. 1971;105(943):201–211. https://doi.org/10.1086/282718.

3. Latta R. Differentiation of Allelic Frequencies at Quantitative Trait Loci Affecting Locally Adaptive Traits. The American Naturalist. 1998;151(3):283–292. https://doi.org/10.1086/286119.

4. Le Corre V, Kremer A. Genetic Variability at Neutral Markers, Quantitative Trait Loci and Trait in a Subdivided Population Under Selection. Genetics. 2003;164(3):1205–1219. https://doi.org/10.1093/genetics/164.3.1205.

5. Kremer A, Le Corre V. Decoupling of differentiation between traits and their underlying genes in response to divergent selection. Heredity. 2012;108(4):375–385. https://doi.org/10.1038/hdy.2011.81.

6. Berg JJ, Coop G. A Population Genetic Signal of Polygenic Adaptation. PLoS Genetics. 2014;10(8):e1004412. https://doi.org/10.1371/journal.pgen.1004412.

7. Consortium GP, et al. A map of human genome variation from population scale sequencing. Nature. 2010;467(7319):1061.

8. Mathieson I, Lazaridis I, Rohland N, Mallick S, Patterson N, Roodenberg SA, et al. Genome-wide patterns of selection in 230 ancient Eurasians. Nature. 2015;528(7583):499–503.
