# Supplementary material for: Testing for differences in polygenic scores in the presence of confounding
Source: bioRxiv. 2023 Mar 23:2023.03.12.532301. Preprint. [Version 2] doi: 10.1101/2023.03.12.532301 (PMC10055004; doi:10.1101/2023.03.12.532301)
Supplement: Supplementary file 1 [file NIHPP2023.03.12.532301V2-supplement-1.pdf]

bioRxiv preprint doi: https://doi.org/10.1101/2023.03.12.532301; this version posted March 23, 2023. The copyright holder for this preprint (which was not certified by peer review) is the author/funder, who has granted bioRxiv a license to display the preprint in perpetuity. It is made available under aCC-BY 4.0 International license.

66. Bhatia G, Patterson N, Sankararaman S, Price AL. Estimating and interpreting FST: the impact of rare variants. Genome research. 2013;23(9):1514–1521.

67. Zaidi AA, Mathieson I. Demographic history mediates the effect of stratification on polygenic scores. Elife. 2020;9:e61548.

68. Chang CC, Chow CC, Tellier LC, Vattikuti S, Purcell SM, Lee JJ. Second-generation PLINK: rising to the challenge of larger and richer datasets. Gigascience. 2015;4(1):s13742–015.

# 6 Supplementary Material

## 6.1 Expectation of polygenic scores using marginal effects

Conditional on the GWAS panel genotypes and the genetic and environmental components of the phenotype, the marginal association at site $\ell$ is given by

$$\begin{aligned}\hat{\beta}_\ell \mid G_\ell, u, e &= \frac{y^\top G_\ell}{G_\ell^\top G_\ell} \\ &= \frac{u^\top G_\ell}{G_\ell^\top G_\ell} + \frac{e^\top G_\ell}{G_\ell^\top G_\ell} \\ &= \sigma_\ell + \epsilon_\ell.\end{aligned} \tag{34}$$

(equation (10) in the main text). Thus, conditioning on all variables in the GWAS panel and the genotypes in the test panel, the polygenic scores are given by

$$Z^\top \mid \mathbf{G}, u, e = \sum_{\ell=1}^{S} \hat{\beta}_\ell X_\ell^\top \tag{35}$$

$$= \sum_{\ell=1}^{S} \sigma_\ell X_\ell^\top + \sum_{\ell=1}^{S} \epsilon_\ell X_\ell^\top \tag{36}$$

$$= \sum_{\ell=1}^{S} \frac{u^\top G_\ell}{G_\ell^\top G_\ell} X_\ell^\top + \sum_{\ell=1}^{S} \frac{e^\top G_\ell}{G_\ell^\top G_\ell} X_\ell^\top. \tag{37}$$

Now, taking the expectation over the joint distribution of genotypes in the GWAS and test panels, conditional $u$ and $e$, the expectation of the vector of polygenic scores is

$$\mathbb{E}_{\mathbf{G},\mathbf{X}}\left[Z^\top \mid u, e\right] \approx \sum_{\ell=1}^{S} u^\top \mathbb{E}\left[\frac{G_\ell X_\ell^\top}{G_\ell^\top G_\ell}\right] + \sum_{\ell=1}^{S} e^\top \mathbb{E}\left[\frac{G_\ell X_\ell^\top}{G_\ell^\top G_\ell}\right] \tag{38}$$

$$\approx S\left(u^\top \mathbb{E}\left[\frac{G_\ell X_\ell^\top}{G_\ell^\top G_\ell}\right] + e^\top \mathbb{E}\left[\frac{G_\ell X_\ell^\top}{G_\ell^\top G_\ell}\right]\right) \tag{39}$$

$$\approx \frac{S}{M-1}\left(u^\top \mathbb{E}\left[\frac{G_\ell X_\ell^\top}{G_\ell^\top G_\ell/(M-1)}\right] + e^\top \mathbb{E}\left[\frac{G_\ell X_\ell^\top}{G_\ell^\top G_\ell/(M-1)}\right]\right) \tag{40}$$

$$\approx \frac{S}{M-1}\left(u^\top + e^\top\right)\tilde{\mathbf{F}}_{GX} \tag{41}$$

bioRxiv preprint doi: https://doi.org/10.1101/2023.03.12.532301; this version posted March 23, 2023. The copyright holder for this preprint (which was not certified by peer review) is the author/funder, who has granted bioRxiv a license to display the preprint in perpetuity. It is made available under aCC-BY 4.0 International license.

where these relationships are approximate because we have ignored the fact that conditioning on the genetic component of the phenotype gives us some information about the distribution of individual causal variants. However, because each variant makes a small contribution to the trait, this effect will be small. Finally, taking the expectations over $u$ and $e$, we have

$$\mathbb{E}\left[Z^\top\right] \approx \frac{S}{M-1}\left(v^\top + c^\top\right)\tilde{\mathbf{F}}_{GX}. \tag{42}$$

## 6.2 Expectation of polygenic scores while controlling for $\tilde{\mathbf{F}}_{Gr}$

Now, controlling for $\tilde{\mathbf{F}}_{\mathbf{Gr}}$, the marginal effects are

$$\begin{aligned}\hat{\beta}'_\ell \mid G_\ell, u, e &= \frac{u^\top \mathbf{P} G_\ell}{G_\ell^\top \mathbf{P} G_\ell} + \frac{e^\top \mathbf{P} G_\ell}{G_\ell^\top \mathbf{P} G_\ell} \\ &= \sigma'_\ell + \epsilon'_\ell,\end{aligned} \tag{43}$$

where $\mathbf{P} = \left(\mathbf{I} - \frac{1}{\|\tilde{F}_{Gr}\|}\tilde{F}_{Gr}\tilde{F}_{Gr}^\top\right)$

Thus, conditional on the variables in the GWAS panel, the polygenic scores are

$$Z^\top \mid \mathbf{G}, u, e = \sum_{\ell=1}^{S} \hat{\beta}'_\ell X_\ell^\top \tag{44}$$

$$= \sum_{\ell=1}^{S} \sigma'_\ell X_\ell^\top + \sum_{\ell=1}^{S} \epsilon'_\ell X_\ell^\top \tag{45}$$

$$= \sum_{\ell=1}^{S} \frac{u^\top \mathbf{P} G_\ell X_\ell^\top}{G_\ell^\top \mathbf{P} G_\ell} + \sum_{\ell=1}^{S} \frac{e^\top \mathbf{P} G_\ell X_\ell^\top}{G_\ell^\top \mathbf{P} G_\ell}. \tag{46}$$

Taking the expectation over the the joint distribution of genotypes in the two panels, the expected polygenic scores conditional on $u$ and $e$ are

$$\mathbb{E}_{\mathbf{G},\mathbf{X}}\left[Z^\top \mid u, e\right] \approx \sum_{\ell=1}^{S} u^\top \mathbf{P}\mathbb{E}\left[\frac{G_\ell X_\ell^\top}{G_\ell^\top \mathbf{P} G_\ell}\right] + \sum_{\ell=1}^{S} e^\top \mathbf{P}\mathbb{E}\left[\frac{G_\ell X_\ell^\top}{G_\ell^\top \mathbf{P} G_\ell}\right] \tag{47}$$

$$\approx S\left(u^\top + e^\top\right)\mathbf{P}\mathbb{E}\left[\frac{G_\ell X_\ell^\top}{G_\ell^\top \mathbf{P} G_\ell}\right] \tag{48}$$

$$\approx \frac{S}{M-1}\left(u^\top + e^\top\right)\mathbf{P}\mathbb{E}\left[\frac{G_\ell X_\ell^\top}{G_\ell^\top G_\ell / M-1}\right]. \tag{49}$$

$$\approx \frac{S}{M-1}\left(u^\top + e^\top\right)\mathbf{P}\tilde{\mathbf{F}}_{GX} \tag{50}$$

$$\approx \frac{S}{M-1}\left(u^\top + e^\top\right)\left(\tilde{\mathbf{F}}_{GX} - \frac{1}{\|\tilde{F}_{Gr}\|}\tilde{F}_{Gr}\tilde{F}_{Gr}^\top\tilde{\mathbf{F}}_{GX}\right) \tag{51}$$

$$\approx \frac{S}{M-1}\left(u^\top + e^\top\right)\tilde{\mathbf{F}}_{GX}^{\perp \tilde{F}_{Gr}} \tag{52}$$

bioRxiv preprint doi: https://doi.org/10.1101/2023.03.12.532301; this version posted March 23, 2023. The copyright holder for this preprint (which was not certified by peer review) is the author/funder, who has granted bioRxiv a license to display the preprint in perpetuity. It is made available under aCC-BY 4.0 International license.

where we make an additional approximation by ignoring the $\mathbf{P}$ in the denominator, which is reasonable so long as $\tilde{F}_{Gr}$ explains only a small fraction of the variance in the GWAS panel genotypes. Taking the expectation over $u$ and $e$, we have

$$\mathbb{E}\left[Z^{\top}\right] \approx \frac{S}{M-1}\left(v^{\top}+c^{\top}\right)\tilde{\mathbf{F}}_{GX}^{\perp \tilde{F}_{Gr}}. \tag{53}$$

## 6.3 Expectation of $\hat{q}$ while controlling for $\tilde{\mathbf{F}}_{Gr}$

The expectation of $\hat{q}$ then becomes

$$\begin{aligned}
\mathbb{E}[\hat{q}] &\approx \frac{S}{(N-1)(M-1)}\left(v^{\top}+c^{\top}\right)\tilde{\mathbf{F}}_{GX}^{\perp \tilde{F}_{Gr}} T && (54)\\
&\approx \frac{S}{(N-1)(M-1)}\left(v^{\top}+c^{\top}\right)\left(\tilde{\mathbf{F}}_{GX}-\frac{1}{\|\tilde{F}_{Gr}\|}\tilde{F}_{Gr}\tilde{F}_{Gr}^{\top}\tilde{\mathbf{F}}_{GX}\right)T && (55)\\
&\approx \frac{S}{(N-1)(M-1)}\left(v^{\top}+c^{\top}\right)\left(\tilde{\mathbf{F}}_{GX}T-\frac{1}{\|\tilde{F}_{Gr}\|}\tilde{F}_{Gr}\tilde{F}_{Gr}^{\top}\tilde{\mathbf{F}}_{GX}T\right) && (56)\\
&\approx \frac{S}{(N-1)(M-1)}\left(v^{\top}+c^{\top}\right)\left(\tilde{F}_{Gr}-\frac{1}{\|\tilde{F}_{Gr}\|}\tilde{F}_{Gr}\tilde{F}_{Gr}^{\top}\tilde{F}_{Gr}\right) && (57)\\
&\approx \frac{S}{(N-1)(M-1)}\left(v^{\top}+c^{\top}\right)\left(\tilde{F}_{Gr}-\tilde{F}_{Gr}\right) && (58)\\
&\approx 0 && (59)
\end{aligned}$$

bioRxiv preprint doi: https://doi.org/10.1101/2023.03.12.532301; this version posted March 23, 2023. The copyright holder for this preprint (which was not certified by peer review) is the author/funder, who has granted bioRxiv a license to display the preprint in perpetuity. It is made available under aCC-BY 4.0 International license.

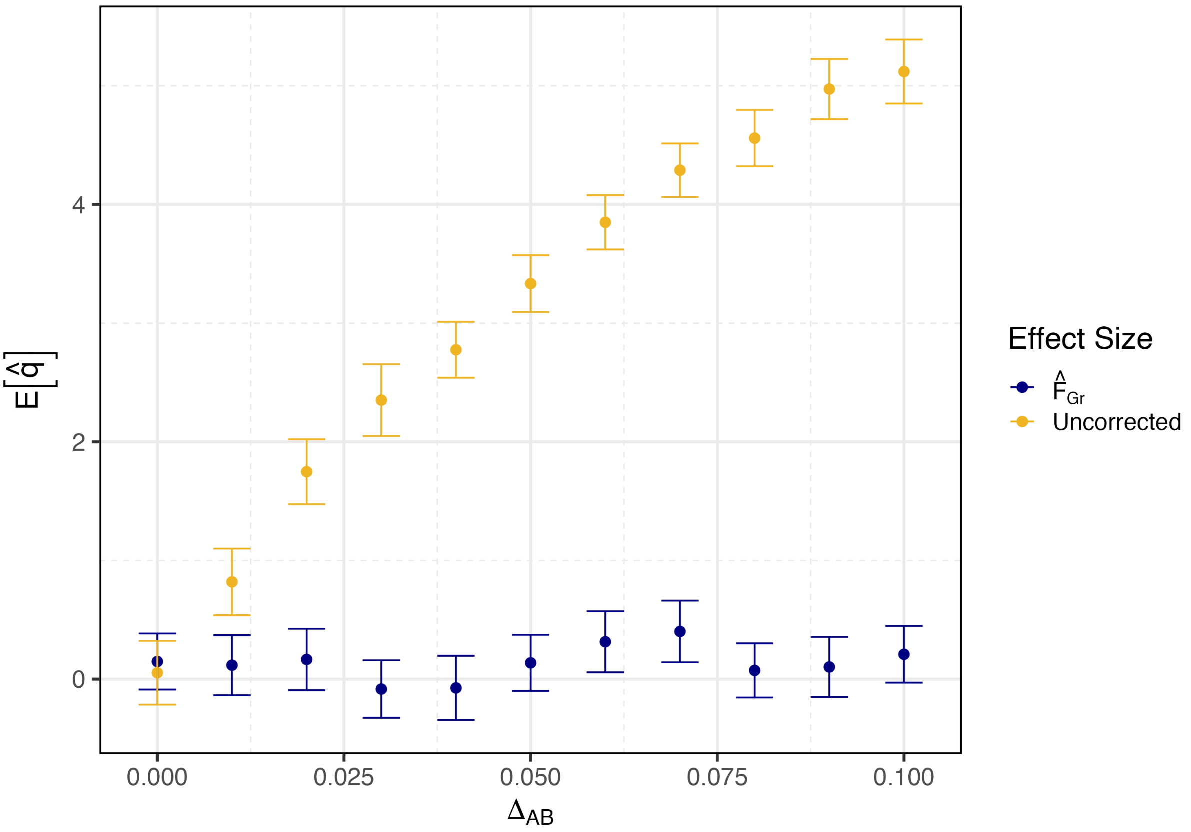


Figure S1: **Including $\hat{F}_{Gr}$ as a covariate removes bias in $\hat{q}$.** In our simulations of the 4 population toy model with overlapping topology (Figure 1A), increasing the magnitude of the expected mean difference in phenotypes for populations A and B, increases the expectation of $\hat{q}$ when effect sizes are uncorrected. When we include $\hat{F}_{Gr}$ as a covariate in the GWAS, $\mathbb{E}[\hat{q}]$ is unbiased.

bioRxiv preprint doi: https://doi.org/10.1101/2023.03.12.532301; this version posted March 23, 2023. The copyright holder for this preprint (which was not certified by peer review) is the author/funder, who has granted bioRxiv a license to display the preprint in perpetuity. It is made available under aCC-BY 4.0 International license.

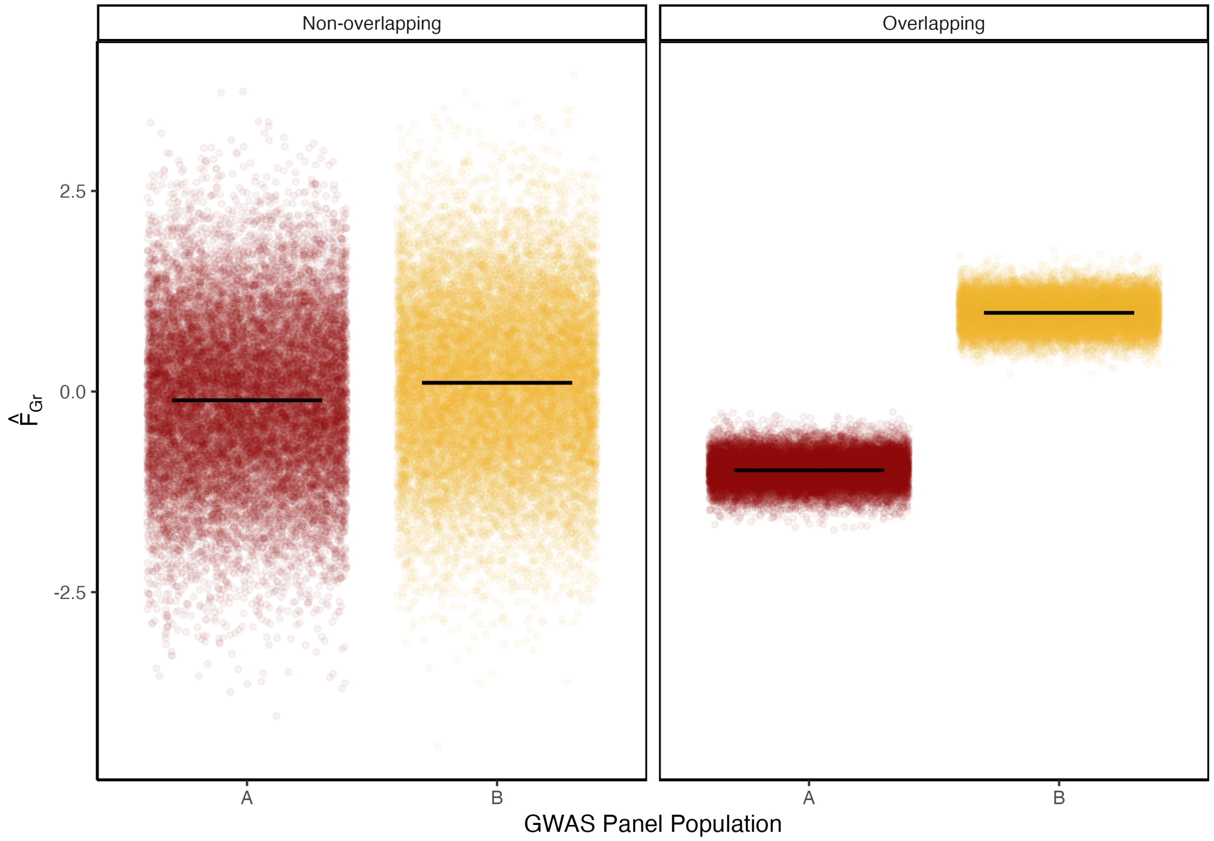


Figure S2: $\hat{F}_{Gr}$ **separates populations in the GWAS panel when test and GWAS panels have overlapping structure.** In the 4 population toy model, when the topology is non-overlapping (Figure 1C), $\hat{F}_{Gr}$, shown here for a single replicate, is purely noise and does not reflect population membership, due to the fact the genotype contrasts in the test panel are already uncorrelated with structure in the GWAS panel. When the topology is overlapping (Figure 1A), $\hat{F}_{Gr}$ separates individuals in the GWAS panel based on population membership.

bioRxiv preprint doi: https://doi.org/10.1101/2023.03.12.532301; this version posted March 23, 2023. The copyright holder for this preprint (which was not certified by peer review) is the author/funder, who has granted bioRxiv a license to display the preprint in perpetuity. It is made available under aCC-BY 4.0 International license.

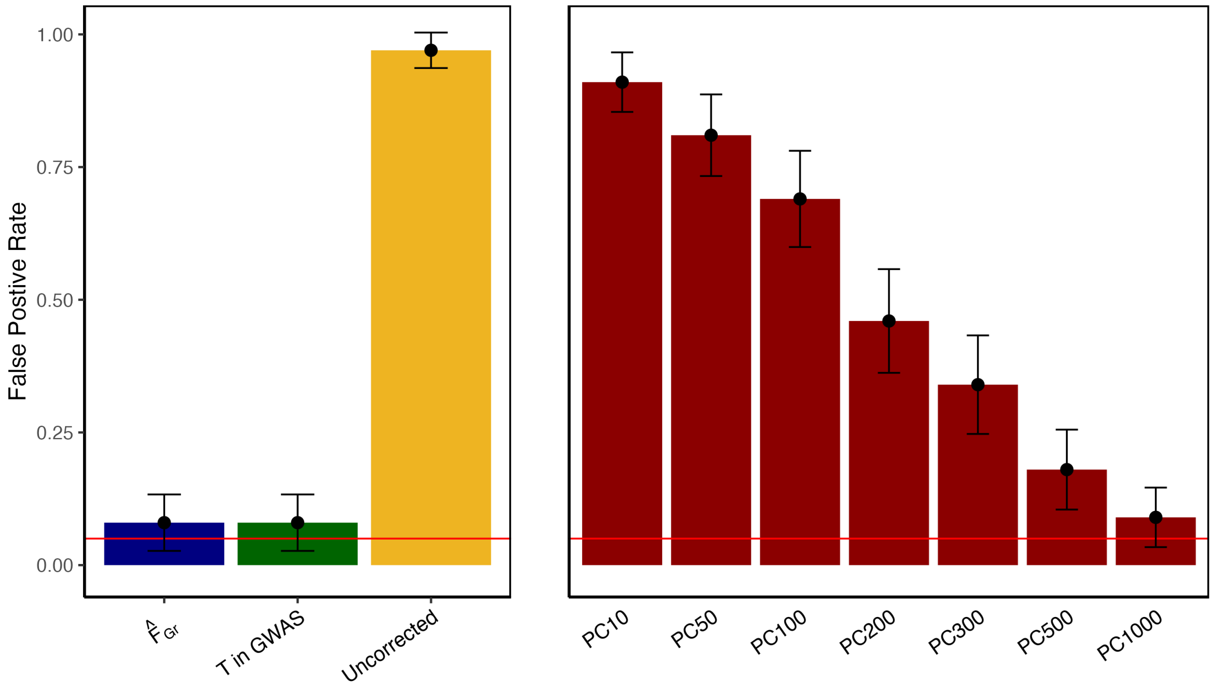


Figure S3: $\hat{F}_{Gr}$ **controls for stratification for a single deme association test.** Here we simulate a 2 standard deviation confounder in a single deme in the GWAS panel (Figure 3D) and conduct an association test for the difference in polygenic score between the same single deme and the rest of the grid in the test panel. Including $\hat{F}_{Gr}$ or the actual test vector, as defined in the GWAS individuals, as a covariate in the GWAS model, controls for stratification and the resulting test is unbiased. We then vary the number of principal components of the GWAS panel included as covariates, demonstrating that it takes 1,000 PCs to fully control for stratification.

bioRxiv preprint doi: https://doi.org/10.1101/2023.03.12.532301; this version posted March 23, 2023. The copyright holder for this preprint (which was not certified by peer review) is the author/funder, who has granted bioRxiv a license to display the preprint in perpetuity. It is made available under aCC-BY 4.0 International license.

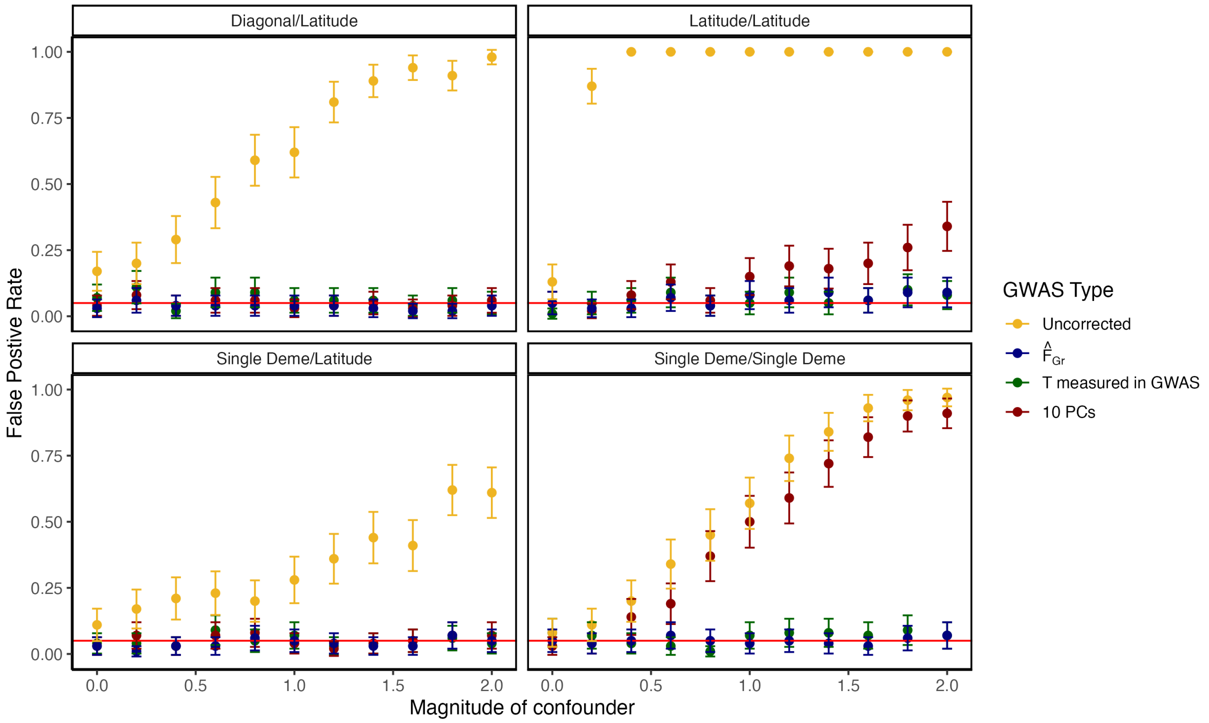


Figure S4: $\hat{F}_{Gr}$ **controls for stratification for a range confounding effects.** For the for simulation scenarios in Figure 3 we plot the false positive rate for the given association test for a range of confounding effects where the magnitude of the confounder is the difference between the most extreme phenotypic shifts in units of standard deviations. For all situations tested, our approach protects the test from stratification bias, mirroring the gold standard of measuring the test vector itself in GWAS panel individuals. In two confounder/test scenarios (right column) the standard approach of including the top 10 GWAS panel principal components begins to fail with large enough confounding.

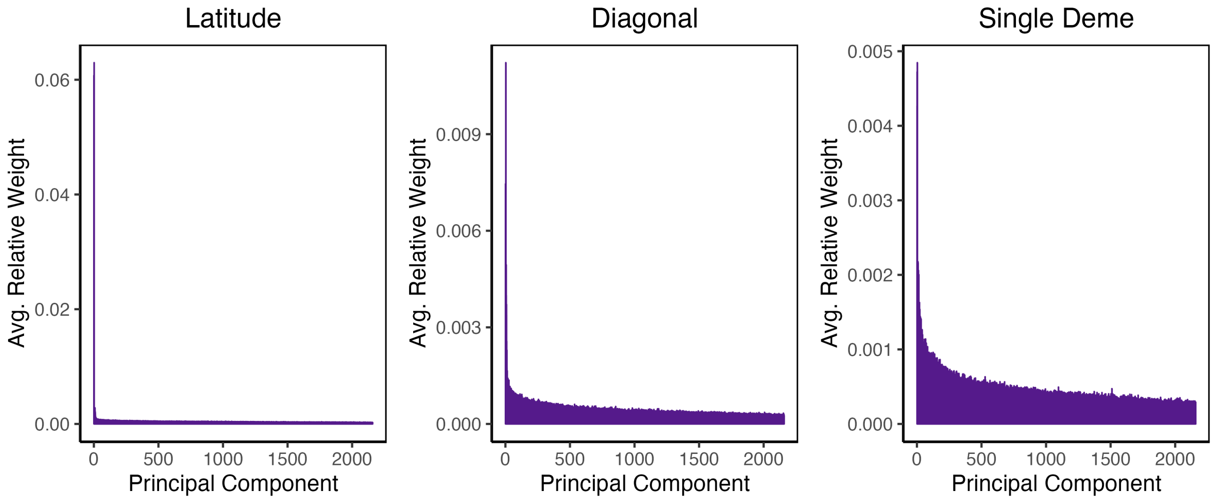


Figure S5: **Different confounding patterns load onto different GWAS principal components.** For the three possible confounding patterns tested in Figure 3 we plot the average relative loading of the confounder onto each of the $M-1$ GWAS panel principal components averaged across all 100 replicates. As the stratification pattern gets more complex, the confounder is more likely to load onto lower PCs, reflecting more subtle structure in the GWAS panel.
